# Tacrolimus Inhibits Hepatic Ferroptosis Through Modulating SIRT7-Dependent NRF2 Activation in Diabetes

**DOI:** 10.3390/antiox15050589

**Published:** 2026-05-06

**Authors:** Siqi Wang, Wenbin Liu, Feng Cui, Xiangji Guo, Jun Li

**Affiliations:** State Key Laboratory of Common Mechanism Research for Major Diseases, Department of Biochemistry & Molecular Biology, Institute of Basic Medical Sciences & School of Basic Medicine, Chinese Academy of Medical Sciences & Peking Union Medical College, Beijing 100005, China; b2022005038@student.pumc.edu.cn (S.W.); s2023005019@pumc.edu.cn (X.G.)

**Keywords:** ferroptosis, tacrolimus, NRF2, SIRT7, diabetes

## Abstract

Hepatic lipotoxicity in type 2 diabetes promotes oxidative stress and ferroptosis, driving progressive liver injury, for which effective targeted therapies remain lacking. Here, we identify tacrolimus (TAC), a clinically established immunosuppressant, as an unexpected suppressor of hepatic ferroptosis in db/db diabetic mice. TAC administration markedly alleviated liver injury, fibrosis, and inflammation, accompanied by reduced oxidative stress and ferroptosis signatures. Transcriptomic profiling revealed enrichment of glutathione metabolism pathways in livers of TAC-treated db/db diabetic mice. Mechanistically, TAC inhibited ferroptosis in primary hepatocytes by activating the NRF2 pathway, increasing NRF2 protein abundance and its nuclear translocation in an SIRT7 deacetylase activity-dependent manner. Together, these findings uncover a previously unrecognized role of TAC in repressing ferroptosis through the SIRT7–NRF2 axis, highlighting ferroptosis modulation by TAC as a potential therapeutic strategy for diabetic liver diseases.

## 1. Introduction

The global prevalence of type 2 diabetes (T2DM) continues to rise, accompanied by an increasing burden of complications, including hepatic steatosis, that significantly impact human health [1]. Diabetic liver disease is characterized by hepatic steatosis, inflammation, and fibrosis [2]. In patients with T2DM, chronic hyperglycemia and insulin resistance drive progressive liver injury through interconnected pathological mechanisms, including lipotoxicity, oxidative stress, and chronic inflammation [3]. In this context, oxidative stress not only results from metabolic dysfunction but also serves as a key driver of hepatocellular injury within this pathogenic cascade [4]. Furthermore, the fatty liver generates excessive reactive oxygen species (ROS) along with impaired antioxidant defenses, creating a vicious cycle that exacerbates metabolic dysfunction and cellular injury, ultimately leading to irreversible hepatic damage [5].

Recent studies have identified ferroptosis, an iron-dependent cell death pathway driven by excessive lipid peroxidation, as a critical contributor to diabetic liver disease [6]. Hepatocytes are particularly susceptible to ferroptosis under diabetic conditions, likely due to their high polyunsaturated fatty acid (PUFA) content and substantial iron stores, both of which are further exacerbated in T2DM [7,8]. In T2DM-associated non-alcoholic steatohepatitis (NASH) mice, hyperglycemia exacerbated hepatic iron overload and lipid peroxidation, leading to PUFA accumulation and subsequent ferroptosis [9]. Huang et al. found that hepatic iron overload-induced ferroptosis is a key risk factor for liver injury in T2DM, suggesting that inhibition of hepatic ferroptosis may represent a promising therapeutic strategy [10]. Consistently, liraglutide attenuated T2DM-associated non-alcoholic fatty liver disease (NAFLD) pathology by activating the AMPK/ACC pathway and suppressing hepatic ferroptosis [11].

Nuclear factor erythroid 2-related factor 2 (NRF2) is a central regulator of cellular defenses against ferroptosis, controlling the expression of genes involved in glutathione biosynthesis, lipid peroxide detoxification, and iron homeostasis [12,13]. Mounting evidence indicates that impaired NRF2 signaling contributes to diabetic liver disease by disrupting redox homeostasis and increasing ferroptosis susceptibility [14]. NRF2 deficiency exacerbates metabolic dysfunction by impairing antioxidant defenses, disturbing lipid metabolism, and inducing endoplasmic reticulum (ER) stress, thereby increasing susceptibility to diabetes [15]. Notably, activation of NRF2 has shown therapeutic potential in diabetic liver disease. For example, mild NRF2 activation by a protein–protein interaction inhibitor PHAR effectively mitigates diabetic liver disease by restoring NRF2 activity to a near-physiological level while avoiding the adverse effects associated with excessive activation by conventional KEAP1 inhibitors [16]. These findings highlight NRF2 as a promising therapeutic target for ferroptosis-associated liver injury in T2DM.

Tacrolimus (TAC), also known as FK506, is a clinically established calcineurin inhibitor widely used for immunosuppression following organ transplantation [17]. TAC suppresses immune responses by inhibiting T-cell activation and proliferation, as well as reducing the release of inflammatory mediators [18]. Beyond its immunosuppressive role, accumulating evidence suggests that TAC also modulates hepatic lipotoxicity and oxidative stress pathways. For instance, TAC ameliorated hepatitis C virus (HCV) core protein-induced hepatic steatosis, insulin resistance, and oxidative stress, while decreasing TNF-α levels and lipid accumulation [19]. Preclinical studies have shown that TAC protects against spinal cord ischemia–reperfusion injury by enhancing endogenous antioxidant activity and reducing oxidative stress markers [20]. Furthermore, TAC significantly reduced free radical species in the livers of rats with severe ischemia–reperfusion injury, thereby improving hepatic function and survival rates [21]. Despite these findings, the potential of TAC to ameliorate oxidative stress in hepatic injury associated with T2DM remains largely unexplored. Elucidating this mechanism may offer new insights into therapeutic strategies for diabetes-associated hepatic injury.

In this study, we demonstrate that TAC exerts hepatoprotective effects in T2DM mice by enhancing antioxidant defenses and inhibiting ferroptosis. Mechanistically, TAC promotes NRF2 transcription and its SIRT7-dependent deacetylation, leading to activation of NRF2 signaling, improved glutathione metabolism, and attenuation of ferroptosis. These findings reveal a previously unrecognized mechanism underlying the hepatoprotective effects of TAC via ferroptosis inhibition, highlighting its potential as a therapeutic strategy for diabetic liver disease.

## 2. Materials and Methods

### 2.1. Chemical Reagents and Antibodies

Tacrolimus monohydrate (HY-13756A) and palmitic acid (HY-N0830) were purchased from MCE (Monmouth Junction, NJ, USA).

The following antibodies were utilized for experimental procedures: HSP90 alpha/beta (sc-13119), TNF alpha (sc-12744), H3 (sc-377009) and GPX4 (sc-166570) were obtained from Santa Cruz Biotechnology (Dallas, TX, USA); H3K18Ac (A22566) and IL6 (A0286) were obtained from ABclonal technology (Wuhan, Hubei, China); Lamin B1 (12987-1-AP) was obtained from Proteintech (Rosemont, IL, USA); GAPDH (GB15004-100) was obtained from Servicebio (Wuhan, Hubei, China); IL6, biotin-conjugated (bs-6309R) was obtained from Bioss (Beijing, China); IL1β (abs115412) was obtained from Absin (Shanghai, China); IL1β, biotin-conjugated (13-7112-81) was obtained from Invitrogen (Carlsbad, CA, USA); SIRT7 (5360) and NRF2 (12721) were obtained from Cell Signaling Technology (Danvers, MA, USA); ACSL4 (F1153), Phospho-NF-κB p65 (Ser536) (F0155), NF-κB p65 (F0006), Phospho-IKB alpha (Ser 36) (F2237), IKB alpha (A5599), Phospho-p38 MAPK(Thr180/Tyr182) (F0159), p38 MAPK (F0171), Phospho-Smad2 (Ser255) (F1689) and Smad2 (F0309) were obtained from Selleck Chemicals (Houston, TX, USA).

### 2.2. Animal Experiments

#### 2.2.1. Study Design

The animal procedure was approved by the Animal Ethics Committee of Institute of Basic Medical Sciences, Chinese Academy of Medical Sciences (Beijing, China). Eight-week male mice were obtained from SPF biotechnology. This study is a randomized controlled animal experiment with a genotype-stratified, body weight-matched, completely randomized design, where the experimental unit is a single mouse. Body weight was set as an important monitored parameter throughout the study, given its close association with the metabolic phenotype of db/db mice. A total of four experimental groups were set based on genotype and intervention, with the final sample size included in the analysis as follows:

db/m control group: C57BKS-db/m male mice with matched baseline body weight, treated with an equal volume of vehicle (*n* = 8); db/m treatment group: C57BKS-db/m mice with matched baseline body weight, treated with TAC (*n* = 8); db/db control group: C57BKS-db/db spontaneous type 2 diabetes model mice with matched baseline body weight, treated with an equal volume of vehicle (*n* = 14); db/db treatment group: C57BKS-db/db spontaneous type 2 diabetes model mice with matched baseline body weight, treated with TAC (*n* = 12). After grouping, an independent-samples *t*-test was used to verify that there was no statistically significant difference in baseline mean body weight between the control and treatment groups within each stratum (*p* > 0.05), confirming successful body weight matching. Vehicle or TAC was injected intraperitoneally three times per week for four weeks. Mice were housed in standard plastic cages with free access to food and water, under controlled environmental conditions (24 ± 1 °C) with 12 h light/dark cycles. To avoid additional environmental confounding, cage positions of all mice were rotated every two weeks to ensure uniform distribution of each group on the animal rack. The order of body weight measurement was also determined according to a random number table to eliminate systematic errors caused by operation order.

A total of 42 mice were used in the final analysis. The sample size of the total enrolled animals was determined based on the well-validated settings of the same db/db mouse model in peer-reviewed published studies [22,23]. This setting fully covers the needs of multi-index detection at different experimental endpoints, ensures sufficient statistical test power for all analyses, and strictly complies with the Reduction requirement of the 3R principles for laboratory animal welfare.

This study adopted a single-blind design for allocation concealment and outcome assessment. The random sequence, grouping table, and drug coding were generated by a researcher who did not participate in animal feeding, experimental operation, or data analysis. The vehicle and TAC were prepared as reagents with identical appearance, volume, and administration frequency with blind codes. The operators performing animal feeding, administration, and body weight measurement were blinded to the grouping information throughout the experiment. All body weight data recording, histopathological section reading, biochemical index detection, and data statistical analysis were completed by researchers who were completely blinded to the grouping information. The grouping information was unblinded only after all experimental data collection and statistical analysis were completed.

#### 2.2.2. Pre-Set Experimental Endpoint and Sample Allocation Scheme

All mice were subjected to the same drug administration procedure. At the end of the experimental cycle, the enrolled mice samples in each group were pre-allocated to several detection cohorts according to the experimental design established a priori: Cohort 1 (db/m mice: *n* = 3, db/db mice: *n* = 6): pre-set for Sirius Red staining; Cohort 2 (db/db mice: *n* = 8): pre-set for qPCR and GSH detection, and three of them were used for RNA sequencing; Cohort 3 (db/db mice: *n* = 10): pre-set for malondialdehyde (MDA) detection; Cohort 4 (six mice per group): pre-set for dihydroethidium (DHE) staining; Cohort 5 (db/db mice: *n* = 5): pre-set for Prussian Blue iron staining; Cohort 6 (db/db mice: *n* = 6–9): pre-set for insulin detection; Cohort 7 (db/db mice: *n* = 6–7): pre-set for Western blotting. Other experiments included all mice. This pre-set allocation scheme was designed to avoid repeated freeze–thaw cycles of samples and ensure the quality of biological samples for each detection and was established before the start of the experiment.

#### 2.2.3. Inclusion and Exclusion Criteria

The a priori inclusion criteria were: mice with a complete administration cycle. The a priori exclusion criteria were: mice that died during administration, and samples with hemolysis, degradation or contamination during collection/processing.

For all quantitative detection data, outliers were identified and excluded using the ROUT method (Q = 1%) in GraphPad Prism 8.0 software. This pre-set method was used to strictly control the impact of extreme values caused by accidental operational errors on the statistical results, with a false discovery rate controlled at 1%. For each independent analysis, the exact number of valid experimental units (final *n* value) in each group is clearly reported in the corresponding figure legend, and all exclusions are traceable in the original data.

### 2.3. Cell Isolation and Culture

Mouse primary hepatocytes were isolated from the livers of C57BL/6J mice via a perfusion-based digestion method using collagenase solution (Servicebio, GC305015). To remove tissue fragments, the homogenate was passed through a 70 µm cell strainer, followed by low-speed centrifugation at 50× *g* rcf for 2 min. The cell pellet was resuspended in DMEM basic medium (Gibco, Waltham, MA, USA, C11885500) with 10% fetal bovine serum (FBS, Gibco, 16140071) and 1% penicillin/streptomycin (P/S) mixture (Solarbio, Beijing, China, P1400). The cell suspension was then layered onto Percoll (Santa Cruz, sc-500790A) and centrifuged at 1300× *g* rpm for 5 min to enrich primary hepatocytes. Isolated primary hepatocytes were cultured in the same medium and maintained in a humidified incubator at 37 °C with 5% CO_2_.

The basic medium of AML12 cells and HEK293T cells was DMEM (VivaCell, Shanghai, China, C3113) supplemented with 10% FBS and 1% P/S. The culture conditions were the same as those for hepatocytes.

### 2.4. Lentiviral Preparations

Short hairpin RNA (shRNA) plasmids targeting SIRT7 (pLKO.1-SIRT7-shRNA) and NRF2 (pLKO.1-NRF2-shRNA) were purchased from RuiBiotech Co., Ltd. (Beijing, China). One day prior to transfection, HEK293T cells were seeded into six-well culture plates at an appropriate density. On the transfection day, cells were transfected with a plasmid mixture consisting of 0.25 µg pMD2.G, 0.75 µg psPAX2, and 1 µg target transfer plasmid, along with 4 µL ExFect Transfection Reagent (Vazyme, Nanjing, Jiangsu, China, T101-01), following the manufacturer’s recommended protocol. The transfection was performed in DMEM medium supplemented with 10% FBS without antibiotics. After 4–12 h of incubation, the transfection medium was replaced with DMEM with 10% FBS and 1% P/S mixture. Viral supernatants were collected after 48 h, filtered through a 0.45 µm syringe filter (PALL), and cryopreserved at −80 °C until subsequent transduction experiments.

### 2.5. Histological Staining

Sirius Red staining was conducted by Wuhan Servicebio Technology Company following a standard protocol. Dihydroethidium (DHE, HY-D0079) was purchased from MCE. The staining procedure was performed as previously described [24]. The Prussian Blue Iron Stain Kit (Ferric Iron, Enhance With DAB, G1428) was obtained from Solarbio and the staining procedure was performed following the manufacturer’s protocol. H&E staining and immunohistochemical staining were conducted by Laike Biotechnology Company (Beijing, China) following standard protocols.

For Oil Red O staining, frozen sections (5 μm) were fixed with 4% polyformaldehyde for 20 min and stained with Oil Red O solution for 30 min at room temperature in the dark. The stained sections were washed with 60% isopropanol followed by PBS and counterstained with hematoxylin for 5 min. Images were captured using an inverted microscope.

### 2.6. Elisa

Mouse INS (Insulin) ELISA Kit (E-EL-M1382) was purchased from Elabscience, and the insulin content was measured following the manufacturer’s instructions.

For IL-6 and IL-1β levels, the antibody was diluted to 2 μg/mL with coating buffer (NaHCO_3_ 5.61 g in 500 mL water, pH 9.5). Each well received 100 μL and shaken overnight at 4 °C. The wells were blocked in 5% BSA for 1 h at room temperature. Standards and samples were added and incubated for 2 h at room temperature. Biotinylated antibodies were added and incubated for 1 h at room temperature. The well plate was cleaned with PBST between each of the above steps. HRP antibody was added and incubated for 1 h at room temperature in the dark. ECL chromogenic solution was added, and the luminescence was read using a microplate reader.

### 2.7. ALT, AST, TBA, GSH and MDA Contents

Alanine Aminotransferase (ALT/GPT) Activity Assay Kit (E-BC-K235-M), Aspartate Aminotransferase (AST/GOT) Activity Assay Kit (E-BC-K236-M), and Total Bile Acid (TBA) Colorimetric Assay Kit (E-BC-K181-M) were purchased from Elabscience (Wuhan, China). Lipid Peroxidation MDA Assay Kit (S0131M) and GSH and GSSG Assay Kit (S0053) were purchased from Beyotime Biotechnology (Shanghai, China). The contents were measured according to the manufacturer’s instructions.

### 2.8. Immunoprecipitation and Western Blotting

For immunoprecipitation assays, cellular proteins were lysed using Triton X-100 lysis buffer to generate total cell lysates. The resulting lysates were subjected to immunoprecipitation using the corresponding primary antibody together with Protein A/G Magnetic Beads (MCE, HY K0202). After incubation and washing, bead-bound proteins were eluted with SDS sample loading buffer. For Western blotting analysis, eluted proteins were separated by SDS-PAGE and then immunoblotted with primary and secondary antibodies.

### 2.9. C11-BODIPY Staining

Intracellular lipid peroxidation was detected using the Lipid Peroxidation Assay Kit with BDPY 581/591 C11 (Beyotime Biotechnology, S0043) following the manufacturer’s protocol. Briefly, cells were incubated with freshly prepared BDPY 581/591 C11 staining working solution (2 μM final concentration, diluted 1:1000 from the 2 mM stock solution with PBS) at 37 °C for 20 min in the dark. Following incubation, the staining solution was completely removed, and cells were washed twice with PBS. Fluorescence images were acquired using an inverted fluorescence microscope.

### 2.10. Site-Directed Mutagenesis of Mouse SIRT7 (H188Y)

The histidine-to-tyrosine substitution at residue 188 (H188Y) of mouse SIRT7 was generated by PCR-based site-directed mutagenesis. The mutagenic primers were designed to introduce the CAT-to-TAC point mutation (encoding H188Y) with flanking homologous sequences, and their sequences were as follows: forward primer 5′-cccgccatctcagagctcTACgggaatatgtatatt-3′ and reverse primer 5′-tggcggtagagtctcgagATGcccttatacatataaccc-3′ (mutated nucleotides are shown in uppercase). The mouse SIRT7 H188Y mutant was generated using high-fidelity DNA polymerase and the indicated mutagenic primers. The wild-type template was removed by DpnI digestion, and mutated plasmids were transformed into DH5α *E. coli*. Positive clones were confirmed by Sanger sequencing covering the entire coding sequence.

### 2.11. QPCR and RNA Sequencing

Total RNA isolation was performed with TRIzol reagent (Vazyme, R401-01) following the manufacturer’s standard protocol. Subsequently, cDNA was reverse-transcribed using the GoScript Reverse Transcription System (Vazyme, R333-01). Quantitative PCR (qPCR) assay was performed using Taq Pro Universal SYBR qPCR Master Mix (Vazyme, Q712-02). The primer pairs used are provided in Table 1. Transcriptome sequencing was conducted by Novogene (Beijing, China).

### 2.12. Statistical Analysis

Statistical analyses were performed using GraphPad Prism 8.0 Software, with experimental data expressed as the mean ± standard error of the mean (SEM). Comparisons between two groups were performed using Student’s *t*-test. One-way analysis of variance (ANOVA) was applied for multiple comparisons relative to the control group. Two-way ANOVA was used to evaluate the interaction effects among multiple variables. Statistical significance is defined as follows: n.s. = not significant, * *p* < 0.05, ** *p* < 0.01, *** *p* < 0.001, and **** *p* < 0.0001.

## 3. Results

### 3.1. TAC Ameliorates Hepatic Injury in Type 2 Diabetic Mice

**Figure 1:**
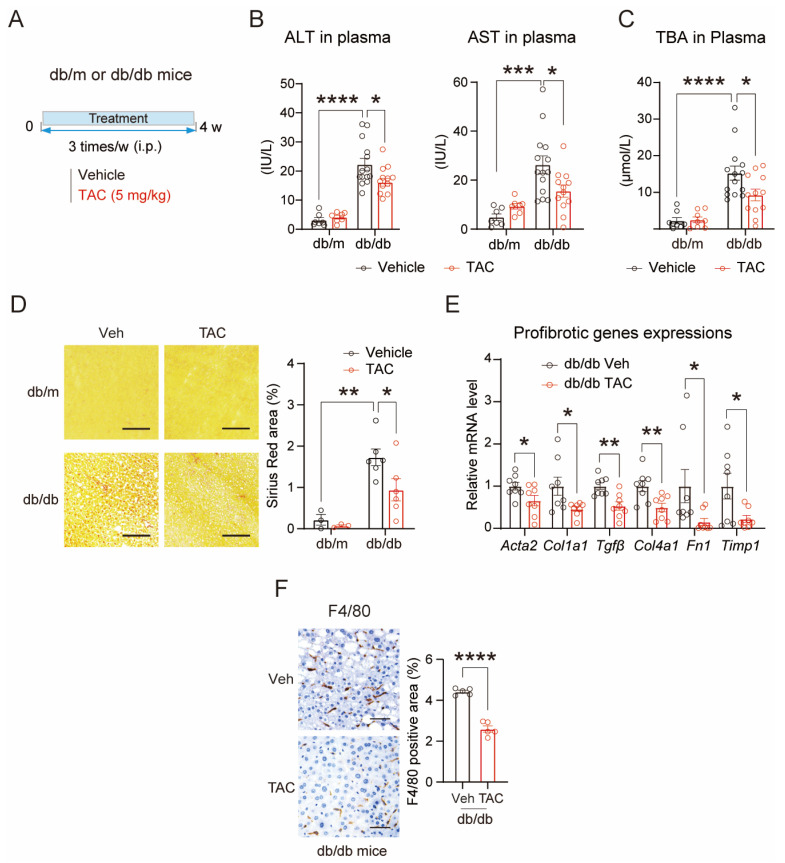
TAC attenuates hepatic injury in diabetic mice. (**A**) Experimental design for the therapeutic model of diabetic mice with TAC treatment. Db/db diabetic mice and their control db/m mice (eight weeks old) were subjected to intraperitoneal (i.p.) injections of vehicle or TAC (5 mg/kg, three times per week) for four weeks (db/m, *n* = 8/group; db/db, *n* = 12–14/group). (**B**) ALT and AST activities in the plasma of TAC-treated db/m and db/db mice (db/m: *n* = 7; db/db: *n* = 12–14). (**C**) Detection of TBA levels in the plasma of db/m and db/db mice treated with TAC (db/m: *n* = 8; db/db: *n* = 12–14). (**D**) Representative images of Sirius Red staining of db/m and db/db mice treated with TAC (db/m: *n* = 3; db/db: *n* = 6). The quantification of collagen deposition is shown on the right. Scale bar, 50 μm. (**E**) Relative expression of fibrosis-related genes in the livers of TAC-treated db/db mice (*n* = 8 for each group), tested by qPCR. (**F**) Representative images of immunohistochemical staining of F4/80 in db/db mice liver sections (*n* = 5 for each group). The quantification is shown in the right panel. Scale bar, 50 μm. Data are shown as mean ± SEM. *p* values are calculated using two-tailed unpaired Student’s *t*-test (**E**,**F**) and one-way ANOVA (**B**–**D**); * *p* < 0.05, ** *p* < 0.01, *** *p* < 0.001, and **** *p* < 0.0001.

To evaluate the therapeutic potential of TAC in diabetic mouse models, we performed a four-week administration in db/m mice and db/db mice. Eight-week-old db/db mice and their lean controls, db/m mice, were grouped according to body weights and treated with TAC or vehicle for four weeks (Figure 1A). TAC significantly reduced plasma markers of hepatic damage, including alanine aminotransferase (ALT), aspartate aminotransferase (AST) and total bile acid (TBA) levels (Figure 1B,C). Four weeks of TAC treatment significantly reduced body weight, with liver weights showing a downward trend (Supplementary Figure S1A,B). Consistent with the changes in body and liver weights, hepatic fat deposition in TAC-treated db/db mice was significantly reduced, as evidenced by H&E and Oil Red O staining (Supplementary Figure S1C,D). Chronic liver injury in db/db mice was associated with pronounced fibrosis, as visualized by Sirius Red staining. Histological analysis revealed that TAC attenuated hepatic fibrosis, with Sirius Red staining showing a marked reduction in collagen deposition (Figure 1D), accompanied by downregulated expression of fibrosis-related genes and macrophage infiltration (Figure 1E,F). Notably, TAC suppressed hepatic inflammation, as evidenced by downregulation of the hepatic NF-κB signaling cascade and the TGF-β/SMAD2 pathway, decreased hepatic expression of pro-inflammatory genes, and reduced circulating IL-6 and IL-1β in db/db mice (Supplementary Figure S1E–H), without affecting plasma insulin levels (Supplementary Figure S1I). Collectively, these findings demonstrate that TAC ameliorates hepatic injury in diabetic mice through the alleviation of fibrosis and inflammation induced by hepatic lipid accumulation.

### 3.2. Transcriptomic Profiling Highlights Glutathione Metabolic Activation with TAC Treatment

**Figure 2:**
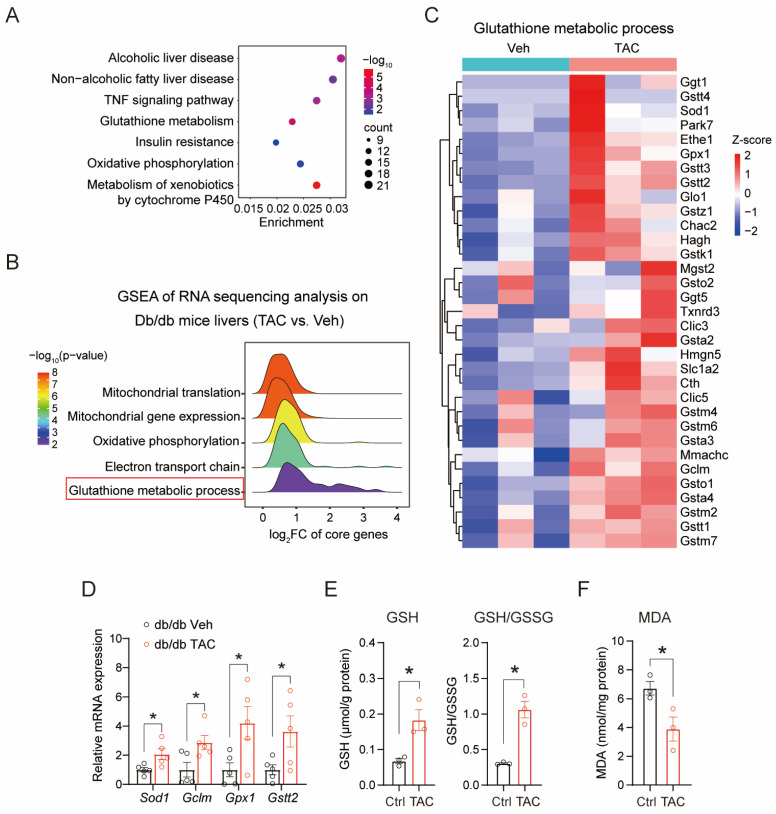
TAC enhances glutathione metabolism and reduces oxidative stress in liver. (**A**) KEGG pathway enrichment analysis of genes in the livers of TAC-treated db/db mice from RNA-seq datasets. (**B**) GSEA of the glutathione metabolic process from RNA-seq datasets of TAC-treated db/db mouse livers. (**C**) Heatmap of genes in the GSEA gene list “GOBP_GLUTATHIONE_METABOLIC_PROCESS”. (**D**) QPCR validation of four selected glutathione metabolism-related genes (*Sod1*, *Gclm*, *Gpx1* and *Gstt2*) in db/db mice livers treated with or without TAC (*n* = five for each group). (**E**) Measurement of GSH, GSSG, and GSH/GSSG levels in primary hepatocytes treated with TAC (20 μM) for 24 h (*n* = 3 biological replicates). Hepatocytes were pre-treated with PA (0.5 mM, 24 h) to induce hepatic lipotoxicity. (**F**) Malondialdehyde (MDA) levels in primary hepatocytes treated with TAC (20 μM) for 24 h (*n* = 3 biological replicates). Hepatocytes were pre-treated with PA (0.5 mM, 24 h) to induce hepatic lipotoxicity. Data are shown as mean ± SEM. *p* values are calculated using two-tailed unpaired Student’s *t*-test (**D**–**F**); * *p* < 0.05.

To elucidate the hepatoprotective mechanisms of TAC in diabetic mice, we conducted transcriptome profiling of hepatic tissues from vehicle- and TAC-treated db/db mice. Principal component analysis (PCA) revealed distinct transcriptional profiles between TAC-treated and control groups (Supplementary Figure S2A). Differential expression analysis identified 1568 differentially expressed genes (DEGs), with 39.8% upregulated and 60.2% downregulated (Supplementary Figure S2B). In accordance with the canonical immunosuppressive mechanism, TAC inhibited the calcineurin–NFAT pathway in the liver, as demonstrated by significantly reduced mRNA expression of the NFAT downstream target genes *Il3*, *Il4* and *Il5* (Supplementary Figure S2C). Kyoto Encyclopedia of Genes and Genomes (KEGG) pathway enrichment of these DEGs highlighted predominant involvement in metabolic regulatory networks, particularly in glutathione metabolism (Figure 2A). Given the central role of glutathione in antioxidant defense, we performed gene set enrichment analysis (GSEA) to further explore this pathway. The glutathione metabolic process gene set exhibited positive enrichment in the livers of TAC-treated db/db mice (Figure 2B). To visualize these changes, we generated a heatmap of genes involved in glutathione metabolism and found TAC upregulated the glutathione biosynthesis and detoxification pathways (Figure 2C). The changes in key glutathione biosynthesis-associated genes, including *Sod1*, *Gclm*, *Gpx1*, *Gstt2,* were validated by qPCR (Figure 2D). These transcriptional changes functionally correlated with enhanced antioxidant capacity, as evidenced by increased glutathione (GSH) levels and GSH/GSSG ratio in palmitic acid (PA)-treated primary hepatocytes (Figure 2E). The antioxidant effects were further corroborated by significant reduction in malondialdehyde (MDA) levels of primary hepatocytes, a marker of lipid peroxidation [25,26] (Figure 2F). Taken together, these findings establish TAC as a potent modulator of hepatic oxidative stress in diabetic mice.

### 3.3. TAC Restores Redox Homeostasis and Attenuates Ferroptosis

**Figure 3:**
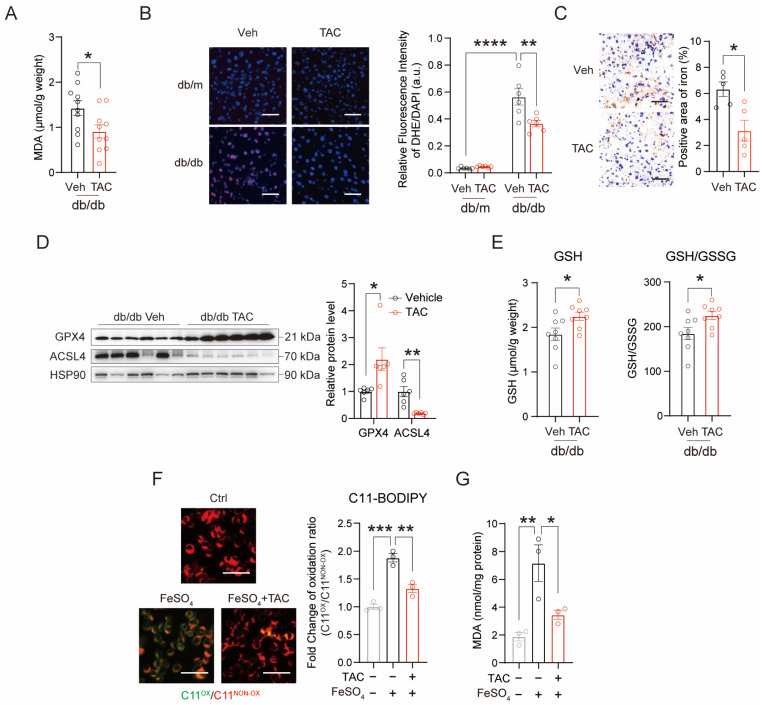
The effects of TAC on glutathione metabolism and oxidative stress. (**A**) MDA levels in the livers of TAC-treated db/db mice (*n* = 10 for each group). (**B**) Representative images and quantification of DHE staining of liver sections from db/m and db/db mice treated with TAC. (*n* = 6 for each group). Scale bar, 50 μm. (**C**) Representative Prussian Blue iron-stained (enhanced with DAB) liver sections from TAC-treated db/db and db/m mice. The quantification is shown on the right (*n* = 5 for each group). Scale bar, 50 μm. (**D**) Western blotting analysis and quantification of GPX4 and ACSL4 in the livers of db/db mice treated with or without TAC (*n* = 6 for each group). The quantification is shown in the right panel. (**E**) Measurement of GSH levels, and GSH/GSSG ratio in the livers of db/db mice treated with or without TAC (*n* = 8 for each group). (**F**) Representative C11-BODIPY staining and quantification of lipid ROS in AML12 cells treated with FeSO_4_ (500 μM) and TAC (20 μM) for 24 h, *n* = 3 biological replicates. Scale bar, 50 μm. (**G**) MDA levels in AML12 cells treated with FeSO_4_ (500 μM) and TAC (20 μM) for 24 h, *n* = 3 biological replicates. Data are shown as mean ± SEM. *p* values are calculated using two-tailed unpaired Student’s *t*-test (**A**,**C**–**E**) and one-way ANOVA (**B**,**F**,**G**); * *p* < 0.05, ** *p* < 0.01, *** *p* < 0.001, and **** *p* < 0.0001.

Building upon the transcriptomic evidence of glutathione pathway activation, we systematically evaluated TAC’s antioxidant effects at biochemical and histological levels in the livers of db/db mice. TAC treatment significantly reduced MDA levels (Figure 3A) and diminished dihydroethidium (DHE) fluorescence intensity (Figure 3B) in the livers of db/db mice, indicating suppression of oxidative stress and superoxide accumulation. Notably, Prussian Blue staining revealed a substantial reduction in hepatic iron deposition following TAC administration (Figure 3C). Meanwhile, TAC treatment significantly increased the protein level of glutathione peroxidase 4 (GPX4), a critical enzyme in glutathione-dependent antioxidant defense [27], and downregulated the expression of acyl-CoA synthetase long-chain family member 4 (ACSL4), which is involved in lipid peroxidation [28] (Figure 3D), concomitant with elevated GSH levels and GSH/GSSG ratio (Figure 3E) in db/db mice livers. These in vivo findings were corroborated in primary hepatocytes treated with PA, where TAC similarly increased GPX4 levels and suppressed ACSL4 expression (Supplementary Figure S3A), while concurrently upregulating the expression of iron metabolism genes (Supplementary Figure S3B). Furthermore, in AML12 cells treated with the ferroptosis inducer FeSO_4_, TAC markedly reduced lipid peroxidation and MDA levels (Figure 3F,G), providing direct evidence that TAC inhibits hepatocyte ferroptosis. These results demonstrate that TAC suppresses ferroptosis by enhancing GPX4-mediated antioxidant defense and reducing iron-dependent lipid peroxidation.

### 3.4. TAC Attenuates Hepatic Oxidative Stress via NRF2 Signaling

**Figure 4:**
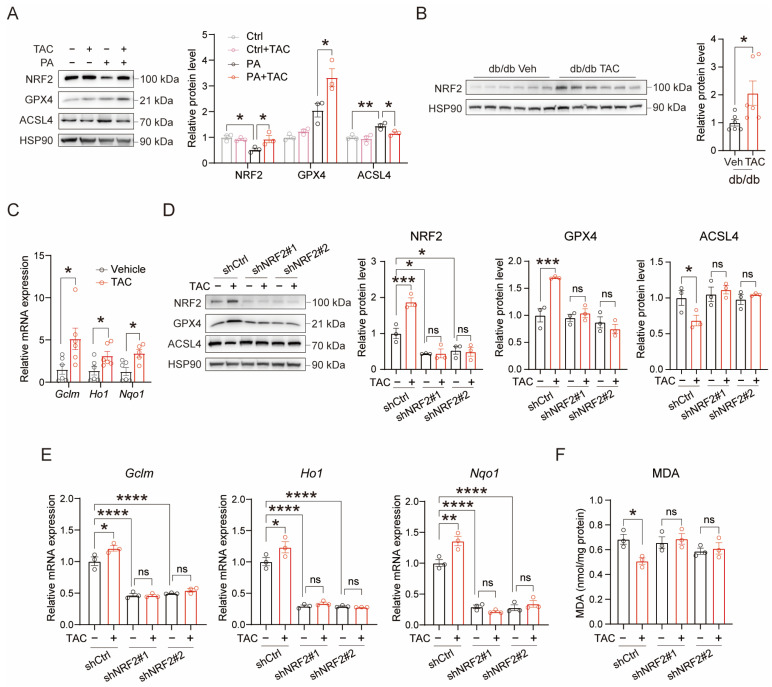
TAC alleviates hepatic lipotoxicity dependent on NRF2 signaling. (**A**) Western blotting analysis and quantification of NRF2, GPX4 and ACSL4 in AML12 cells treated with TAC (20 μM) for 24 h (*n* = 3 biological replicates). AML12 cells were pre-treated with or without PA (0.5 mM, 24 h) to induce hepatic lipotoxicity. (**B**) Western blotting analysis and quantification of NRF2 in db/db mice livers treated with or without TAC (*n* = 6 for each group). (**C**) Relative expression of target genes of NRF2 in db/db mice treated with TAC (*n* = 5–6 for each group). (**D**) Western blotting analysis and quantification of NRF2, GPX4 and ACSL4 in control and NRF2 knockdown AML12 cells treated with TAC (20 μM) for 24 h, *n* = 3 biological replicates. AML12 cells were pre-treated with PA (0.5 mM, 24 h) to induce hepatic lipotoxicity. (**E**) Relative expression of target genes of NRF2 in control and NRF2 knockdown AML12 cells treated with TAC (20 μM) for 24 h, *n* = 3 biological replicates. AML12 cells were pre-treated with PA (0.5 mM, 24 h) to induce hepatic lipotoxicity. (**F**) MDA levels in control and NRF2 knockdown AML12 cells treated with TAC (20 μM) for 24 h (*n* = 3 biological replicates). AML12 cells were pre-treated with PA (0.5 mM, 24 h) to induce hepatic lipotoxicity. Data are shown as mean ± SEM. *p* values are calculated using two-tailed unpaired Student’s *t*-test (**B**,**C**) and one-way ANOVA (**A**,**D**–**F**); * *p* < 0.05, ** *p* < 0.01, *** *p* < 0.001, and **** *p* < 0.0001.

To elucidate the mechanism of TAC’s antioxidant effects, we investigated the regulation of NRF2 signaling by TAC in both cellular and animal models. In PA-induced AML12 cells, TAC administration upregulated NRF2 while concomitantly increasing GPX4 and suppressing ACSL4 levels (Figure 4A). Meanwhile, hepatic NRF2 expression was significantly elevated by TAC treatment in db/db mice (Figure 4B), with increased expression levels of NRF2 target genes (Figure 4C). Conversely, NRF2 knockdown abolished TAC-induced NRF2 and GPX4 upregulation and ACSL4 suppression (Figure 4D). Consistent with these changes, NRF2 knockdown eliminated TAC-induced transcriptional activation of antioxidant response genes (Figure 4E). Most critically, the antioxidant capacity of TAC was severely compromised in NRF2-depleted cells, as evidenced by persistent elevation of MDA levels despite TAC treatment (Figure 4F). These studies demonstrate that NRF2 serves as an essential mediator of TAC’s protective effects against hepatic oxidative injury.

### 3.5. TAC Improves SIRT7 Activity to Enhance NRF2 Expression and Deacetylation

**Figure 5:**
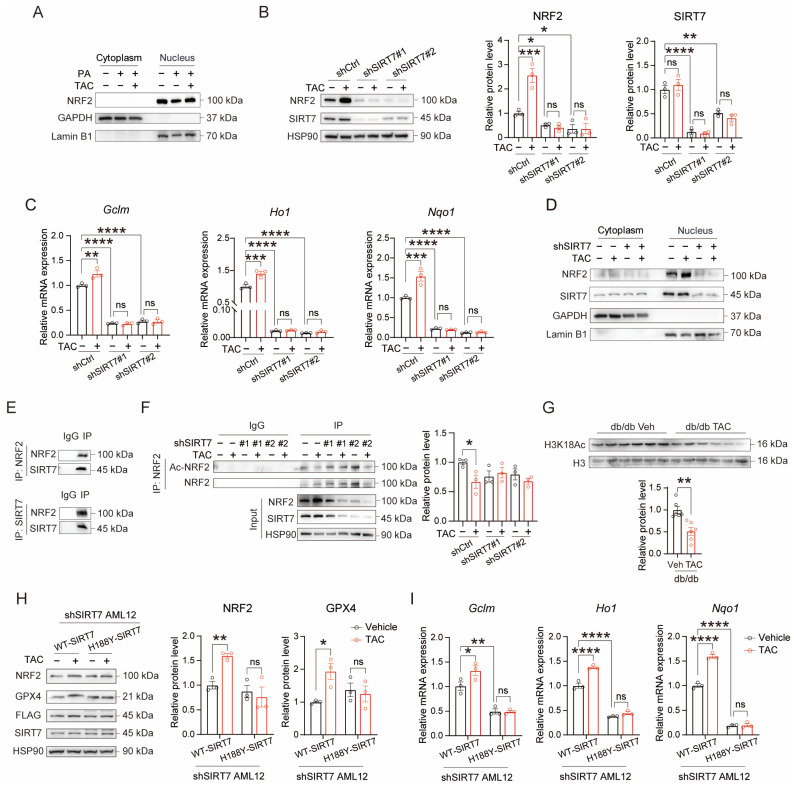
TAC activates SIRT7 to promote NRF2 expression and deacetylation in hepatic lipotoxicity. (**A**) Relative protein levels of NRF2 in the nuclear or cytoplasmic fractions were examined by Western blotting in AML12 cells treated with TAC (20 μM) for 24 h, *n* = 3 biological replicates. AML12 cells were pre-treated with PA (0.5 mM, 24 h) to induce hepatic lipotoxicity. (**B**) Western blotting analysis and quantification of NRF2 and SIRT7 in control and SIRT7 knockdown AML12 cells treated with TAC (20 μM) for 24 h, *n* = 3 biological replicates. AML12 cells were pre-treated with PA (0.5 mM, 24 h) to induce hepatic lipotoxicity. (**C**) Relative expression of target genes of NRF2 in control and SIRT7 knockdown AML12 cells treated with TAC (20 μM) for 24 h, *n* = 3 biological replicates. AML12 cells were pre-treated with PA (0.5 mM, 24 h) to induce hepatic lipotoxicity. (**D**) Relative protein levels of NRF2 and SIRT7 in nuclear or cytoplasmic fractions were examined by Western blotting in control and SIRT7 knockdown AML12 cells treated with TAC (20 μM) for 24 h, *n* = 3 biological replicates. AML12 cells were pre-treated with PA (0.5 mM, 24 h) to induce hepatic lipotoxicity. (**E**) Immunoprecipitation of SIRT7 and NRF2 in HEK293T cells, followed by Western blotting analysis to detect NRF2 and SIRT7. (**F**) Immunoprecipitation of NRF2 in control and SIRT7 knockdown AML12 cells treated with TAC (20 μM), followed by Western blotting analysis with acetyl-lysine antibody to detect acetylation status of NRF2. AML12 cells were pre-treated with PA (0.5 mM, 24 h) to induce hepatic lipotoxicity, *n* = 3 biological replicates. The quantification is shown in the right panel. (**G**) Western blotting analysis and quantification of H3K18Ac in db/db mice livers treated with or without TAC (*n* = 6 for each group). (**H**) Western blotting analysis and quantification of NRF2 and GPX4 in SIRT7-knockdown AML12 cells reconstituted with wild-type (WT) SIRT7 or catalytically inactive mutant (H188Y) SIRT7, followed by TAC (20 μM, 24 h) treatment. AML12 cells were pre-treated with PA (0.5 mM, 24 h) to induce hepatic lipotoxicity, *n* = 3 biological replicates. (**I**) Relative expression of NRF2 downstream genes in SIRT7-knockdown AML12 cells reconstituted with WT-SIRT7 or H188Y-SIRT7, followed by TAC treatment (20 μM, 24 h). AML12 cells were pre-treated with PA (0.5 mM, 24 h) to induce hepatic lipotoxicity, *n* = 3 biological replicates. Data are shown as mean ± SEM. *p* values are calculated using two-tailed unpaired Student’s *t*-test (**F**–**H**) and one-way ANOVA (**B**,**C**,**I**); * *p* < 0.05, ** *p* < 0.01, *** *p* < 0.001, and **** *p* < 0.0001.

In AML12 cells, TAC significantly promoted nuclear accumulation of NRF2 (Figure 5A). According to Yu’s study [29], SIRT7 could both enhance the expression and increase nuclear accumulation of NRF2; therefore, we next investigated whether TAC regulates SIRT7 activity to increase the expression of NRF2. The results showed that SIRT7 knockdown abolished TAC-induced upregulation of both NRF2 protein (Figure 5B) and its downstream target genes (Figure 5C), while also inhibiting nuclear accumulation of NRF2 (Figure 5D). Co-immunoprecipitation revealed a direct interaction between SIRT7 and NRF2 (Figure 5E). Importantly, TAC treatment markedly reduced NRF2 acetylation, but this effect was lost with SIRT7 depletion (Figure 5F), demonstrating that TAC regulates SIRT7 activity. The deacetylase activity of SIRT7 was evidenced by reduced H3K18 acetylation, an established target of SIRT7 [30]. TAC significantly decreased H3K18 acetylation levels in AML12 cells and in the livers of db/db mice, whereas SIRT7 knockdown completely abolished this effect (Figure 5G and Supplementary Figure S4). This evidence suggests that TAC’s effect may depend on the deacetylation activity of SIRT7. This notion is further supported by the observation that reintroduction of H188Y-SIRT7 into SIRT7-deficient AML12 cells failed to increase NRF2 and GPX4 protein levels or induce the expression of downstream antioxidant genes (Figure 5H,I). Collectively, these results demonstrate that TAC exerts its antioxidant effects through SIRT7-dependent deacetylation of NRF2.

## 4. Discussion

This study demonstrates that TAC exerts a noncanonical hepatoprotective effect by inhibiting ferroptosis in type 2 diabetic mice. We show that TAC administration improves glutathione homeostasis and suppresses ferroptosis via the SIRT7/NRF2 antioxidant pathway, leading to activation of NRF2 signaling and upregulation of key genes in glutathione metabolism. These findings provide new insights into the therapeutic potential of TAC in diabetic liver disease and highlight its potential for repurposing for metabolic disorders, leveraging established safety profiles to accelerate clinical translation.

Hepatic injury is a complex pathological process driven by multiple factors, including hepatic steatosis [31,32], inflammation [33,34] and ferroptosis [35,36]. Hepatic steatosis is widely regarded as a major driver of liver damage progression in diabetic conditions [37,38]. Our findings revealed that TAC administration markedly improves multiple parameters of hepatic injury in diabetic mice, including reductions in serum markers of hepatocyte damage, such as ALT, AST and TBA. TAC also significantly attenuated collagen deposition and caused a slight reduction in liver weight, supporting its hepatoprotective effects. While alleviating hepatic steatosis is a recognized strategy for hepatoprotection in T2DM [39], our study identified TAC as a novel candidate that mitigates diabetes-associated liver injury by suppressing ferroptosis. Notably, the clinical translation of TAC for chronic diabetic liver disease requires careful consideration of its inherent immunosuppressive properties, as long-term administration of TAC is associated with safety concerns due to increased susceptibility to infections. Although our study demonstrates hepatoprotective effects of TAC in diabetic mice, future studies are needed to optimize dosing strategies, develop liver-targeted delivery approaches, or design non-immunosuppressive TAC derivatives that retain the ability to activate the SIRT7–NRF2 axis. Such efforts may enhance the therapeutic potential of targeting this pathway in diabetic liver dysfunction.

Oxidative stress is a key contributor to T2DM pathophysiology, arising from an imbalance between ROS production and antioxidant defenses [40]. Glutathione (GSH), a major cellular antioxidant crucial for ROS neutralization, is substantially depleted in T2DM, exacerbating oxidative stress and promoting hepatic injury [41]. Our transcriptomic analysis revealed glutathione metabolism as a pathway modulated by TAC. Consistently, TAC restored hepatic glutathione redox homeostasis, alleviated lipid peroxidation, and attenuated ferroptosis in diabetic mice. This suppression of ferroptosis represents a promising therapeutic strategy targeting diabetic liver disease. TAC is a widely used immunosuppressive agent that has been extensively proven to ameliorate inflammatory responses and exert protective effects against inflammation-associated tissue injury across multiple organs. For example, TAC suppressed TNF-α-induced NF-κB activation and MCP-1 production in renal tubular cells via unfolded protein response (UPR)-mediated upregulation of C/EBP family members [42]. Although TAC is a well-established inhibitor of the calcineurin–NFAT pathway that regulates inflammation [18], our data indicate that its effects on ferroptosis are predominantly mediated through activation of the SIRT7–NRF2 axis in hepatocytes, while a contributory role of NFAT signaling cannot be excluded. Consistently, TAC also effectively reduced the expression of inflammatory mediators in the livers of diabetic mice. Therefore, the dual action of TAC, simultaneously inhibiting ferroptosis and inflammation, confers a distinct advantage over single-target agents. Given that inflammation and ferroptosis are mutually reinforcing in T2DM-related liver injury [43], the ability of TAC to counteract both pathological processes makes it a more comprehensive therapeutic candidate, with the potential to show better therapeutic effects compared to managing a single process. Notably, although the db/db genetic model is well-established for studying type 2 diabetes, evaluation of TAC in a diet-induced model would further strengthen the translational relevance of our findings and represents an important direction for future investigation.

NRF2, a master regulator of cellular antioxidant responses, has emerged as a promising target for treating T2DM complications, including diabetic nephropathy, cardiomyopathy, and neuropathy [44]. Several compounds, including MG132 [45], sulforaphane [46], and chlorogenic acid [47,48], exert renal protection in T2DM via NRF2/HO-1-mediated antioxidant defenses while suppressing NF-κB-driven inflammation. Similarly, allopurinol protects against diabetic cardiomyopathy through NRF2-mediated reductions in apoptosis and oxidative stress [49]. In diabetic neuropathy, enhancement of SIRT1-mediated NRF2 activation by polydatin confers neuroprotection [50], while fisetin [51] and resveratrol [52] act through dual mechanisms, simultaneously activating NRF2 signaling and suppressing NF-κB-mediated inflammation. Our study discovers that TAC significantly enhances hepatic NRF2 protein levels and the antioxidant system, including GSH and GPX4. Importantly, the hepatoprotective effects of TAC in T2DM are NRF2-dependent, establishing TAC as a novel modulator of NRF2. Given that NRF2 represents a promising target in diabetes-related complications, our findings extend the mechanistic understanding of TAC and suggest its therapeutic potential in other NRF2-related diseases.

SIRT7 has been implicated in cardiovascular and metabolic disease progression through NRF2-dependent mechanisms. For instance, in vascular aging and calcification models, *Ganoderma lucidum* spore powder (GLSP) upregulates SIRT7 expression, which interacts with Keap1 to promote its deacetylation, disrupt Keap1-NRF2 binding, facilitate NRF2 nuclear translocation, and activate downstream antioxidant genes, ultimately alleviating vascular oxidative stress and calcification [53]. Similarly, in calcific aortic valve disease (CAVD), the natural flavanone hesperetin exerts protective effects by directly binding to SIRT7 and upregulating its expression, enabling activation of NRF2-ARE signaling and suppression of inflammatory cytokine production and ROS accumulation [54]. These studies underscore a non-redundant role for SIRT7 in regulating NRF2-mediated antioxidant responses. Consistent with these findings, our results show that SIRT7 mediates NRF2 activation in T2DM, supporting a conserved SIRT7-NRF2 regulatory axis across diverse metabolic stress contexts. Recent advances further show that SIRT7 promotes NRF2 nuclear localization and enhances antioxidant responses [29]. We demonstrate that TAC promotes SIRT7-dependent NRF2 nuclear accumulation and deacetylation in diabetic mouse livers. Loss of TAC’s protective effects in SIRT7-knockdown cells, together with reduced H3K18 acetylation, indicates that SIRT7 deacetylase activity is required for TAC-induced inhibition of ferroptosis. Together, our study shows that TAC activates SIRT7-NRF2 axis to exert hepatoprotective effects in T2DM, linking its previously unrecognized metabolic benefits to a well-characterized antioxidant signaling network. Further in vivo genetic studies will be an important future direction to fully define its mechanistic contribution.

In summary, our findings uncover a previously unrecognized function of TAC in repressing ferroptosis through the SIRT7-NRF2 axis, expanding its biological scope beyond immunosuppression and highlighting the potential for repurposing clinically available drugs for diabetic liver dysfunction. Future studies are needed to define optimal dosing or combination strategies for TAC in patients with T2DM-related liver dysfunction, balancing metabolic benefits with potential immunosuppressive effects.

## 5. Conclusions

This study reveals that TAC ameliorates diabetic hepatic injury by inhibiting ferroptosis. Our findings demonstrate that TAC improves hepatic redox homeostasis by activating an SIRT7-NRF2 antioxidant axis, which enhances the expression and deacetylation levels of hepatic NRF2 and coordinately upregulates antioxidant defenses, thereby providing a potential therapeutic strategy for diabetes-associated liver dysfunction.

## Figures and Tables

**Table 1:** Primers used in this study.

Genes	Gene Accession Number	Forward (5′-3′)	Reverse (5′-3′)
*Acta2*	NM_007392.3	TGCTGACAGAGGCACCACTGAA	CAGTTGTACGTCCAGAGGCATAG
*Col1a1*	NM_007742.4	CCTCAGGGTATTGCTGGACAAC	CAGAAGGACCTTGTTTGCCAGG
*Tgfβ*	NM_011577.2	TGATACGCCTGAGTGGCTGTCT	CACAAGAGCAGTGAGCGCTGAA
*Col4a1*	NM_009931.2	ATGGCTTGCCTGGAGAGATAGG	TGGTTGCCCTTTGAGTCCTGGA
*Fn1*	NM_010233.2	CCCTATCTCTGATACCGTTGTCC	TGCCGCAACTACTGTGATTCGG
*Timp1*	NM_001044384.2	TCTTGGTTCCCTGGCGTACTCT	GTGAGTGTCACTCTCCAGTTTGC
*Il1α*	NM_010554.4	ACGGCTGAGTTTCAGTGAGACC	CACTCTGGTAGGTGTAAGGTGC
*Il1β*	NM_008361.4	GGATGAGGACATGAGCACCT	AGCTCATATGGGTCCGACAG
*Il18*	NM_008360.2	GACAGCCTGTGTTCGAGGATATG	TGTTCTTACAGGAGAGGGTAGAC
*Tnf-α*	NM_013693.3	GGTGCCTATGTCTCAGCCTCTT	GCCATAGAACTGATGAGAGGGAG
*Cxcl2*	NM_009140.2	CATCCAGAGCTTGAGTGTGACG	GGCTTCAGGGTCAAGGCAAACT
*Ccl2*	NM_011333.3	GCTACAAGAGGATCACCAGCAG	GTCTGGACCCATTCCTTCTTGG
*Fpn*	NM_016917.2	CCATAGTCTCTGTCAGCCTGCT	CTTGCAGCAACTGTGTCACCGT
*Fth*	NM_010239.2	CAAGTGCGCCAGAACTACCA	ACAGATAGACGTAGGAGGCATAC
*Ftl*	NM_010240.2	CCTCGAGTTTCAGAACGATCGC	CCTGATTCAGGTTCTTCTCCATG
*Actin*	NM_007393.5	CATTGCTGACAGGATGCAGAAGG	TGCTGGAAGGTGGACAGTGAGG
*Gclm*	NM_008129.4	AGGAGCTTCGGGACTGTATCC	GGGACATGGTGCATTCCAAAA
*Ho1*	NM_010442.2	CACTCTGGAGATGACACCTGAG	GTGTTCCTCTGTCAGCATCACC
*Nqo1*	NM_008706.5	GCCGAACACAAGAAGCTGGAAG	GGCAAATCCTGCTACGAGCACT
*Sod1*	NM_011434.2	GGTGAACCAGTTGTGTTGTCAGG	ATGAGGTCCTGCACTGGTACAG
*Gpx1*	NM_001329527.1	CGCTCTTTACCTTCCTGCGGAA	AGTTCCAGGCAATGTCGTTGCG
*Gstt2*	NM_001428510.1	ATGCCGACAACATCCGTGGTAC	AGCTGTTGCAGAACCAGGACCA
*Il3*	NM_010556.4	CCTGCCTACATCTGCGAATGAC	GAGGTTAGCACTGTCTCCAGATC
*Il4*	NM_021283.2	ATCATCGGCATTTTGAACGAGGTC	ACCTTGGAAGCCCTACAGACGA
*Il5*	NM_010558.1	GATGAGGCTTCCTGTCCCTACT	TGACAGGTTTTGGAATAGCATTTCC

## Data Availability

The RNA-Seq data presented in the study are openly available in the ScienceDB repository at https://doi.org/10.57760/sciencedb.31415. Further inquiries can be directed to the corresponding author.

## Supplementary Materials

The following supporting information can be downloaded at: https://www.mdpi.com/article/10.3390/antiox15050589/s1, Figure S1: TAC ameliorates obesity and hepatic inflammation in db/db mice; Figure S2: Transcriptomic analysis in the livers of TAC-treated db/db mice; Figure S3: TAC modulates ferroptosis in primary hepatocytes; Figure S4: TAC enhances SIRT7 deacetylase activity in AML12 cells; Table S1: Differentially expressed genes from RNA-seq analysis of db/db mice livers (TAC vs. Veh; *p*-value < 0.05).

## Abbreviations

The following abbreviations are used in this manuscript:

T2DM	type 2 diabetes mellitus
ROS	reactive oxygen species
PUFA	polyunsaturated fatty acid
NASH	non-alcoholic steatohepatitis
NAFLD	non-alcoholic fatty liver disease
NRF2	nuclear factor erythroid 2-related factor 2
ER	endoplasmic reticulum
TAC	tacrolimus
HCV	hepatitis C virus
ALT	alanine aminotransferase
AST	aspartate aminotransferase
TBA	total bile acid
PCA	principal component analysis
DEGs	differentially expressed genes
KEGG	Kyoto Encyclopedia of Genes and Genomes
GSEA	gene set enrichment analysis
MDA	malondialdehyde
DHE	dihydroethidium
GPX4	glutathione peroxidase 4
ACSL4	acyl-CoA synthetase long-chain family member 4
PA	palmitic acid
