# Supplementary material for: Tacrolimus Inhibits Hepatic Ferroptosis Through Modulating SIRT7-Dependent NRF2 Activation in Diabetes
Source: Antioxidants (Basel). 2026 May 6;15(5):589. doi: 10.3390/antiox15050589 (PMC13203482; doi:10.3390/antiox15050589)
Supplement: Supplementary file 1 [file antioxidants-15-00589-s001.zip › Table S1.pdf]

Table S1. Differentially expressed genes from RNA-seq analysis of db/db mice livers (TAC vs. Veh; p-value < 0.05)

	Gene_ID	Gene_Name	Veh_1	Veh_2	Veh_3	TAC_1	TAC_2	TAC_3	log2FoldChange	p-value
1	ENSMUSG00000042659	Arrdc4	1034.413	166.7794	747.4303	137.6539	121.021	126.905	-2.33566	0.000101
2	ENSMUSG00000022863	Btg3	303.4057	32.54233	210.6019	17.10952	26.24552	32.0749	-2.85153	0.000104
3	ENSMUSG00000037171	Nodal	19.17124	15.5932	119.754	3.110823	5.103295	2.091841	-3.83861	0.000116
4	ENSMUSG00000027332	Ivd	425.1013	1373.557	545.0873	2809.851	2863.677	3243.748	1.924035	0.000116
5	ENSMUSG00000020429	Igfbp1	41887.49	2084.065	28480.81	1992.482	5308.156	1635.122	-3.01933	0.000119
6	ENSMUSG00000038576	Susd4	4.16766	12.20337	0	27.2197	104.9821	79.48996	3.430641	0.000121
7	ENSMUSG00000059743	Fdps	650.9885	246.1013	487.275	13.221	27.7036	143.6398	-2.90485	0.000121
8	ENSMUSG00000064147	Rab44	10.00238	22.37285	78.45953	1.555411	2.916168	0	-4.5782	0.000125
9	ENSMUSG00000021750	Fam107a	0	0	0	20.99805	44.47157	4.183682	6.571924	0.000128
10	ENSMUSG00000021091	Serpina3n	131661.4	5277.28	80722.47	8609.202	12420.69	7725.867	-2.92015	0.00013
11	ENSMUSG00000046070	Igfals	150.8693	300.3385	214.7313	1435.645	641.5571	534.1168	1.969388	0.000133
12	ENSMUSG00000089940	Gm4117	1.667064	12.20337	74.33008	0.777706	0	0	-6.73643	0.000133
13	ENSMUSG00000039286	Fndc3b	2250.537	547.7958	1858.252	360.0777	452.7352	322.8408	-2.03463	0.000135
14	ENSMUSG00000009614	Sardh	1287.807	6749.821	2531.352	9862.863	17775.5	24043.62	2.289271	0.00014
15	ENSMUSG00000074882	Cyp2c68	333.4128	1081.354	1036.492	2840.181	3078.016	2987.846	1.864066	0.000143
16	ENSMUSG00000022843	Clcn2	125.0298	254.2369	293.1909	847.6992	637.9118	688.2157	1.706538	0.000153
17	ENSMUSG00000021109	Hif1a	3022.387	724.7447	2382.692	389.6305	573.0271	552.2461	-2.01581	0.000161
18	ENSMUSG00000039450	Dcxr	231.7219	593.2195	441.851	2459.883	917.864	1510.309	1.948544	0.000161
19	ENSMUSG00000051225	Fam83a	227.5543	17.62709	111.4951	0.777706	6.561379	19.52385	-3.72538	0.000162
20	ENSMUSG00000030483	Cyp2b10	13.33651	216.2709	276.6731	3599.222	640.828	610.1203	3.270664	0.000166
21	ENSMUSG00000005667	Mthfd2	200.8812	14.23727	313.8381	23.33117	21.14222	15.34017	-3.13013	0.000176
22	ENSMUSG00000025197	Cyp2c23	533.4605	885.4224	396.4271	1122.229	2774.734	2782.846	1.874437	0.000183
23	ENSMUSG00000025004	Cyp2c40	8.335321	116.61	103.2362	174.2061	649.5765	986.6517	3.002286	0.000188
24	ENSMUSG00000028961	Pgd	1011.074	937.6257	937.3849	372.521	274.1198	441.3785	-1.40772	0.000193
25	ENSMUSG00000004558	Ndrg2	3755.895	8994.563	5574.756	19243.55	16920.34	18530.23	1.577338	0.000206
26	ENSMUSG00000078675	Mup16	0	2.033895	0	3.888528	15.30988	113.6567	5.491392	0.000208
27	ENSMUSG00000040268	Plekha1	604.3107	785.0836	660.7118	171.0952	271.9327	286.5822	-1.49095	0.000214
28	ENSMUSG00000003541	Ier3	382.5912	7.457616	330.3559	37.32987	10.20659	15.34017	-3.51545	0.000215
29	ENSMUSG00000026189	Pecr	548.4641	1193.219	1086.045	3072.715	2892.839	2398.645	1.566354	0.000221
30	ENSMUSG00000032997	Chpf	109.1927	35.93215	177.5663	20.99805	17.49701	18.12929	-2.47923	0.000226
31	ENSMUSG00000055003	Lrtm2	10.00238	14.23727	0	67.66039	28.43264	164.5582	3.217311	0.000228

Table S1. Differentially expressed genes from RNA-seq analysis of db/db mice livers (TAC vs. Veh; p-value < 0.05)

	Gene_ID	Gene_Name	Veh_1	Veh_2	Veh_3	TAC_1	TAC_2	TAC_3	log2FoldChange	p-value
32	ENSMUSG00000032902	Slc16a1	2629.794	983.0494	2023.43	495.3985	599.2726	667.9946	-1.67636	0.000238
33	ENSMUSG00000030228	Pik3c2g	282.5674	364.7452	243.6375	118.2113	73.63325	103.8948	-1.59852	0.000244
34	ENSMUSG00000049685	Cyp2g1	6.668256	2.71186	0	41.99611	28.43264	52.29603	3.419419	0.000245
35	ENSMUSG00000085977	Gm5970	342.5817	20.33895	206.4724	19.44264	20.41318	34.16674	-2.94007	0.000252
36	ENSMUSG00000022885	St6gal1	5478.806	1158.642	3336.595	539.7277	854.4374	1022.91	-2.04478	0.000254
37	ENSMUSG00000025574	Tk1	59.18078	159.3218	57.81228	601.1665	295.2621	289.3714	2.074162	0.000254
38	ENSMUSG00000110439	Gm21320	5.834724	52.20331	0	694.4912	5421.157	170.1364	6.725326	0.000262
39	ENSMUSG00000015243	Abca1	16274.71	2233.217	9869.383	1712.508	2474.369	1981.671	-2.20168	0.000269
40	ENSMUSG00000027171	Prrg4	120.8621	12.20337	218.8608	5.44394	16.03893	16.03745	-3.20413	0.000273
41	ENSMUSG00000025479	Cyp2e1	7242.56	60330.76	24421.56	162571.6	140944.3	137457.7	2.261022	0.000281
42	ENSMUSG00000022546	Gpt	1413.67	2694.911	1230.576	7497.86	4065.868	5247.732	1.6528	0.0003
43	ENSMUSG00000032348	Gsta4	24.17243	315.2538	136.2718	766.8178	923.6964	1027.791	2.509372	0.000304
44	ENSMUSG00000097815	Gm26809	305.0727	147.7964	276.6731	61.43875	67.80092	88.55461	-1.7374	0.000306
45	ENSMUSG00000032578	Cish	378.4236	40.67791	330.3559	57.55022	16.03893	38.35042	-2.7383	0.000309
46	ENSMUSG00000034450	Gulo	687.6639	2164.743	1036.492	3176.15	5722.252	4788.922	1.813878	0.000317
47	ENSMUSG00000053644	Aldh7a1	1700.405	4008.808	2337.268	6242.643	9016.793	8885.444	1.584655	0.00032
48	ENSMUSG00000023905	Tnfrsf12a	916.8853	61.01686	1168.634	160.9851	75.82038	69.72804	-2.80487	0.000328
49	ENSMUSG00000021539	Lect2	163.3723	599.3212	623.5468	2881.399	1595.144	1193.744	2.03706	0.000343
50	ENSMUSG00000028001	Fga	493318.4	22166.07	363763.2	56657.41	48519.21	27656.23	-2.72665	0.00035
51	ENSMUSG00000017765	Slc12a4	793.5225	265.7623	635.9351	154.7634	174.9701	175.7147	-1.7433	0.000365
52	ENSMUSG00000055629	B4galnt4	40.84307	0	78.45953	1.555411	0.729042	0	-5.69979	0.000367
53	ENSMUSG00000037583	Nr0b2	53.34605	52.88128	289.0614	1456.643	436.6962	418.3682	2.586497	0.000371
54	ENSMUSG00000102752	Gm7694	1352.823	107.7965	685.4885	113.545	117.3758	153.4017	-2.48121	0.000371
55	ENSMUSG00000005677	Nr1i3	195.88	584.4059	363.3915	2325.34	844.2308	1197.23	1.931457	0.000384
56	ENSMUSG00000029273	Sult1d1	537.6282	1372.201	1366.848	5674.918	4226.986	2020.021	1.86502	0.000388
57	ENSMUSG00000001131	Timp1	29.17362	11.52541	37.16504	2.333117	1.458084	2.091841	-3.68457	0.000397
58	ENSMUSG00000032959	Pebp1	1942.963	3444.063	2225.773	7003.24	6131.973	6338.279	1.354193	0.000415
59	ENSMUSG00000030946	Lhpp	675.161	421.0163	524.44	1253.662	1345.812	1466.381	1.326535	0.000418
60	ENSMUSG00000045730	Adrb2	518.4569	139.6608	247.7669	104.9903	62.69762	48.80963	-2.06903	0.000422
61	ENSMUSG00000061048	Cdh3	88.3544	10.16948	103.2362	3.110823	0	10.45921	-3.87082	0.000426
62	ENSMUSG00000027333	Smox	480.948	67.79651	231.2491	52.10628	51.76199	55.08515	-2.29626	0.000429

Table S1. Differentially expressed genes from RNA-seq analysis of db/db mice livers (TAC vs. Veh; p-value < 0.05)

	Gene_ID	Gene_Name	Veh_1	Veh_2	Veh_3	TAC_1	TAC_2	TAC_3	log2FoldChange	p-value
63	ENSMUSG00000086332	4930480G23Rik	0	9.491512	12.38835	77.77057	72.17517	44.62594	3.325865	0.000438
64	ENSMUSG00000005413	Hmox1	1408.669	157.9659	1362.718	219.313	203.4027	160.3745	-2.32664	0.000446
65	ENSMUSG00000107296	Gm43500	0.833532	8.813546	276.6731	6.999351	2.916168	1.394561	-4.63974	0.000447
66	ENSMUSG00000002233	Rhoc	321.7434	89.49139	330.3559	59.88334	70.71708	52.29603	-2.00975	0.000452
67	ENSMUSG00000030088	Aldh1l1	4905.336	9029.817	5442.614	11699.8	19621.44	27670.87	1.605842	0.00047
68	ENSMUSG00000035356	Nfkbiz	2030.484	170.1692	1267.741	313.4154	188.0929	99.71109	-2.52808	0.000481
69	ENSMUSG00000038132	Rbm24	6.668256	27.79657	28.90614	0	0	1.394561	-5.43598	0.000481
70	ENSMUSG00000040724	Kcna2	3.334128	12.20337	8.258898	40.44069	72.90421	48.11235	2.76006	0.000491
71	ENSMUSG00000026473	Glul	2749.822	5418.975	4187.261	7861.827	15971.85	11904.67	1.532265	0.000509
72	ENSMUSG00000070644	Etnk2	1130.269	1722.709	2378.563	6152.43	4635.25	3805.756	1.482509	0.00051
73	ENSMUSG00000026950	Neb	25.00596	79.99988	8.258898	139.2093	320.7785	185.4766	2.430368	0.000515
74	ENSMUSG00000039384	Dusp10	280.9003	23.05081	256.0258	34.21905	30.61977	23.70753	-2.65294	0.00052
75	ENSMUSG00000028039	Efna3	85.02027	5.423721	16.5178	0	0	3.486402	-4.931	0.000537
76	ENSMUSG00000030087	Klf15	349.2499	893.558	867.1843	2455.994	2464.162	1599.561	1.630603	0.000539
77	ENSMUSG00000026527	Rgs7	0.833532	1.35593	57.81228	0	0	0	-7.13532	0.000562
78	ENSMUSG00000064347	mt-Ta	8.335321	3.389826	202.343	6.999351	2.187126	1.394561	-4.30455	0.000571
79	ENSMUSG00000094156	Sult2a7	7.501788	467.7959	454.2394	2148.023	1867.806	3388.085	2.997815	0.000571
80	ENSMUSG00000002384	Bmp8b	6.668256	3.389826	49.55339	0	0.729042	0	-6.14491	0.000599
81	ENSMUSG00000053303	Slc22a26	6.668256	86.77953	24.77669	114.3227	202.6737	538.9977	2.824946	0.000619
82	ENSMUSG00000026979	Psd4	287.5686	98.30494	342.7443	38.10758	60.5105	82.27908	-1.99731	0.00062
83	ENSMUSG00000115377	Gm4942	1.667064	2.71186	45.42394	0	0	0	-6.85075	0.000644
84	ENSMUSG00000027009	Itga4	290.0692	157.9659	528.5695	58.32793	118.8339	83.67365	-1.88992	0.000645
85	ENSMUSG00000049971	Glt1d1	321.7434	636.6092	330.3559	1633.96	1050.55	1052.893	1.529812	0.000647
86	ENSMUSG00000053219	Raet1e	153.3699	303.7284	256.0258	84.76992	86.02697	64.84708	-1.59532	0.000649
87	ENSMUSG00000037664	Cdkn1c	12.50298	25.08471	0	141.5424	61.96858	67.6362	2.663716	0.000658
88	ENSMUSG00000053113	Socs3	1917.957	29.1525	1779.792	241.8665	95.50452	102.5002	-3.08201	0.000677
89	ENSMUSG00000073424	Cyp4f15	165.0393	1249.49	433.5921	3476.344	2833.787	1941.926	2.155667	0.000688
90	ENSMUSG00000085914	Gm14320	5.834724	5.423721	78.45953	1.555411	1.458084	0.69728	-4.51002	0.000691
91	ENSMUSG00000072623	Zfp9	69.18316	29.83046	222.9902	19.44264	10.20659	21.61569	-2.61073	0.000701
92	ENSMUSG00000054855	Rnd1	1111.098	28.47453	1201.67	123.6552	129.7695	49.50691	-2.94825	0.000705
93	ENSMUSG00000029380	Cxcl1	8029.414	40.67791	7887.247	241.0888	90.40122	23.01025	-5.49234	0.000711

Table S1. Differentially expressed genes from RNA-seq analysis of db/db mice livers (TAC vs. Veh; p-value < 0.05)										
	Gene_ID	Gene_Name	Veh_1	Veh_2	Veh_3	TAC_1	TAC_2	TAC_3	log2FoldChange	p-value
94	ENSMUSG00000085573	Gm15418	18.33771	0.677965	24.77669	0	0	0	-6.71454	0.000756
95	ENSMUSG00000078964	Ces1b	15.00358	233.898	210.6019	617.4983	972.5422	901.5835	2.448128	0.000791
96	ENSMUSG00000022146	Osmr	404.263	91.52529	297.3203	27.9974	89.67218	59.26883	-2.16044	0.0008
97	ENSMUSG00000090217	Gm16116	0.833532	3.389826	45.42394	0	0	0	-6.85054	0.000803
98	ENSMUSG00000040270	Bach2	179.2094	73.22023	181.6958	15.55411	43.74253	43.23138	-2.06921	0.000814
99	ENSMUSG00000052560	Cpne8	914.3847	50.84738	813.5014	132.21	99.14973	68.33348	-2.56695	0.000826
100	ENSMUSG00000000078	Klf6	308.4069	279.9996	235.3786	57.55022	96.9626	126.905	-1.55371	0.00087
101	ENSMUSG00000041698	Slco1a1	9.168853	73.8982	12.38835	223.2015	286.5135	94.13285	2.597568	0.000878
102	ENSMUSG00000046688	Tifa	7859.374	63.05076	6326.316	229.4232	196.8414	168.7419	-4.58169	0.000883
103	ENSMUSG00000024526	Cidea	158.3711	107.1185	227.1197	64.54957	18.95509	7.670084	-2.42679	0.000893
104	ENSMUSG00000067279	Ppp1r3c	12.50298	174.915	115.6246	671.16	894.5347	218.2488	2.561184	0.000902
105	ENSMUSG00000074607	Tox2	27.50656	2.033895	41.29449	1.555411	0.729042	0.69728	-4.52785	0.000911
106	ENSMUSG00000040511	Pvr	350.917	92.88122	396.4271	89.43615	59.05241	68.33348	-1.94438	0.000916
107	ENSMUSG00000022091	Sorbs3	167.5399	265.7623	338.6148	508.6195	746.5391	860.444	1.467729	0.000947
108	ENSMUSG00000090213	Tmem189	1268.636	666.4397	1069.527	492.2877	330.9851	243.3509	-1.49349	0.000953
109	ENSMUSG00000022500	Litaf	5098.716	1132.88	3869.294	1076.345	990.0392	926.6856	-1.75438	0.000986
110	ENSMUSG00000057160	Gm16372	0.833532	1.35593	49.55339	0	0	0	-6.91411	0.000997
111	ENSMUSG00000028469	Npr2	155.8705	128.8134	210.6019	694.4912	384.2052	338.181	1.532783	0.000997
112	ENSMUSG00000115919	Gm31583	5.001192	17.62709	33.03559	88.65845	66.34283	101.8029	2.361183	0.001008
113	ENSMUSG00000104983	Gm42763	0.833532	0.677965	53.68284	0	0	0	-7.01417	0.001011
114	ENSMUSG00000029695	Aass	1606.216	3100.334	1936.712	4585.353	7291.15	5384.399	1.376738	0.001012
115	ENSMUSG00000022000	Zc3h13	2202.192	279.3216	1726.11	473.6228	226.0031	248.9291	-2.14837	0.00102
116	ENSMUSG00000040706	Agmat	739.3429	1475.93	1003.456	3389.241	2195.875	2754.955	1.3726	0.001047
117	ENSMUSG00000047617	Paxx	40.84307	44.06773	53.68284	232.534	86.75601	155.4935	1.797932	0.001056
118	ENSMUSG00000045790	Ccdc149	91.68853	12.88134	99.10677	9.332468	14.58084	4.880963	-2.80078	0.001075
119	ENSMUSG00000029622	Arpc1b	670.1598	386.4401	578.1228	194.4264	223.0869	234.2862	-1.32458	0.001082
120	ENSMUSG00000029445	Hpd	1757.919	9357.952	12057.99	26330	35712.13	27782.44	1.955051	0.001092
121	ENSMUSG00000020732	Rab37	19.17124	0.677965	49.55339	0.777706	0.729042	0	-5.4837	0.001092
122	ENSMUSG00000035849	Krt222	15.00358	1.35593	86.71843	0.777706	2.916168	0	-4.74581	0.001095
123	ENSMUSG00000027698	Nceh1	2378.9	1063.727	1573.32	549.8379	739.2487	613.6067	-1.39874	0.001097
124	ENSMUSG00000025203	Scd2	350.0835	151.8642	132.1424	43.55152	64.15571	77.39812	-1.78758	0.001138

Table S1. Differentially expressed genes from RNA-seq analysis of db/db mice livers (TAC vs. Veh; p-value < 0.05)

	Gene_ID	Gene_Name	Veh_1	Veh_2	Veh_3	TAC_1	TAC_2	TAC_3	log2FoldChange	p-value
125	ENSMUSG00000039457	Ppl	140.0334	96.94901	144.5307	171.0952	483.3549	649.8653	1.781762	0.00116
126	ENSMUSG00000062480	Acat3	785.1872	1229.151	908.4788	1907.712	2234.514	2815.618	1.250119	0.001196
127	ENSMUSG00000030562	Nox4	162.5388	170.8472	144.5307	349.9676	355.0435	449.7458	1.265095	0.001202
128	ENSMUSG00000049225	Pdp1	81.68614	28.47453	66.07118	6.999351	13.12276	14.64289	-2.32782	0.001223
129	ENSMUSG00000020312	Shc2	41.6766	23.72878	0	0.777706	1.458084	0.69728	-4.53889	0.001226
130	ENSMUSG00000079197	Psme2	152.5364	124.7456	49.55339	801.0368	298.1782	216.1569	1.981668	0.001244
131	ENSMUSG00000055730	Ces2a	552.6317	1503.049	941.5144	4097.731	2088.706	2690.805	1.565723	0.001253
132	ENSMUSG00000051483	Cbr1	351.7505	635.9313	520.3106	2799.74	866.102	1177.707	1.683717	0.001259
133	ENSMUSG00000030364	Clec2h	0	0	0	30.33052	17.49701	1.394561	6.07013	0.001309
134	ENSMUSG00000076441	Ass1	3595.024	10981	3997.307	16447.7	24029.23	16959.25	1.628189	0.001312
135	ENSMUSG00000042216	Sgsm1	117.528	37.96605	123.8835	24.88658	21.87126	20.22113	-2.03993	0.001318
136	ENSMUSG00000057228	Aadat	304.2392	846.1005	404.686	1327.544	1752.617	1411.993	1.525905	0.001322
137	ENSMUSG00000026819	Slc25a25	1562.873	1266.439	1676.556	3267.919	2763.07	4213.665	1.18632	0.001326
138	ENSMUSG00000021493	Pdlim7	140.8669	84.74564	247.7669	55.99481	36.45211	42.5341	-1.78402	0.001327
139	ENSMUSG00000026489	Coq8a	460.1097	1572.879	924.9966	2202.462	2895.026	4169.737	1.646787	0.001334
140	ENSMUSG00000010476	Ebf3	19.17124	7.457616	33.03559	0	0	2.091841	-4.74825	0.001357
141	ENSMUSG00000069324	Gm5096	956.8948	4254.231	1618.744	4095.398	8259.318	14510.4	1.974981	0.001374
142	ENSMUSG00000052726	Kcnt2	67.5161	8.813546	322.097	26.44199	10.93563	2.789122	-3.28965	0.00139
143	ENSMUSG00000019838	Slc16a10	261.7291	565.4229	161.0485	677.3816	1729.288	1019.424	1.782451	0.001403
144	ENSMUSG00000002910	Arrdc2	140.8669	187.7963	111.4951	766.0401	359.4178	270.5448	1.651842	0.001415
145	ENSMUSG00000046840	Hnf4aos	2.500596	19.66099	0	106.5457	40.09732	59.26883	3.001495	0.001415
146	ENSMUSG00000097482	Gm17634	0	8.813546	0	89.43615	32.80689	23.01025	3.756469	0.001418
147	ENSMUSG00000047492	Inhbe	2671.47	1714.574	1205.799	626.0531	723.2098	837.4337	-1.35677	0.001426
148	ENSMUSG00000097804	Gm16685	13.33651	2.71186	28.90614	0	0.729042	0	-5.75881	0.001438
149	ENSMUSG00000038055	Dexi	75.01788	75.25413	16.5178	376.4095	187.3638	134.5751	2.005602	0.001457
150	ENSMUSG00000004187	Kifc2	35.00835	71.18634	33.03559	295.5282	130.4985	110.1703	1.910234	0.001481
151	ENSMUSG00000108415	Gm30146	0	5.423721	45.42394	0	0	0	-6.90469	0.001519
152	ENSMUSG00000041261	Car8	118.3616	397.9655	136.2718	576.2799	753.8295	813.0289	1.701787	0.001549
153	ENSMUSG00000063628	Gm7665	14.17004	2.033895	103.2362	4.666234	0.729042	1.394561	-4.08305	0.001552
154	ENSMUSG00000027907	S100a11	630.9838	158.6438	1143.857	258.976	51.76199	70.42532	-2.33825	0.001553
155	ENSMUSG00000038745	Nlrp6	1272.803	1364.744	924.9966	6852.365	1860.515	2623.866	1.668492	0.001554

Table S1. Differentially expressed genes from RNA-seq analysis of db/db mice livers (TAC vs. Veh; p-value < 0.05)										
	Gene_ID	Gene_Name	Veh_1	Veh_2	Veh_3	TAC_1	TAC_2	TAC_3	log2FoldChange	p-value
156	ENSMUSG00000024027	Glp1r	2.500596	2.71186	33.03559	0	0	0	-6.45793	0.001556
157	ENSMUSG00000025138	Sirt7	240.0572	331.5249	198.2135	882.6959	520.5361	582.2291	1.356276	0.001582
158	ENSMUSG00000043079	Synpo	699.3334	378.9825	491.4044	185.8717	129.0405	259.3883	-1.45135	0.001586
159	ENSMUSG00000032440	Tgfbr2	1632.889	850.8462	1147.987	252.7543	506.6843	555.0352	-1.46628	0.001596
160	ENSMUSG00000061751	Kalrn	178.3759	218.3048	218.8608	580.9461	355.0435	571.7699	1.296173	0.001617
161	ENSMUSG00000042988	Notum	30.00715	146.4405	99.10677	486.8437	253.7067	290.7659	1.906771	0.001619
162	ENSMUSG00000045776	Lrtm1	95.02265	176.9489	309.7087	616.7206	426.4896	704.2532	1.612312	0.001622
163	ENSMUSG00000042292	Mkl1	392.5936	88.81343	214.7313	55.2171	57.59433	78.79268	-1.86199	0.001651
164	ENSMUSG00000030630	Fah	2229.698	4844.061	3555.456	11935.45	7994.676	7382.107	1.361459	0.001693
165	ENSMUSG00000032010	Usp2	39.17601	351.1859	152.7896	825.9234	715.9194	686.1239	2.03065	0.001722
166	ENSMUSG00000010601	Apol7a	185.8776	831.8632	218.8608	984.5754	1423.09	2312.879	1.925465	0.001724
167	ENSMUSG00000022149	C9	1186.116	1827.116	1251.223	3740.764	5384.705	2196.433	1.407662	0.00174
168	ENSMUSG00000023030	Slc11a2	1271.97	378.9825	900.2199	248.0881	352.1273	251.0209	-1.58265	0.001745
169	ENSMUSG00000090021	Gm6493	180.8765	9.491512	161.0485	17.88723	19.68414	17.43201	-2.66456	0.001753
170	ENSMUSG00000026821	Ralgds	278.3997	29.83046	148.6602	34.99676	26.97456	32.77218	-2.26946	0.001754
171	ENSMUSG00000032898	Fbxo21	300.9051	727.4566	342.7443	655.6059	1314.463	3036.656	1.864912	0.001803
172	ENSMUSG00000021190	Lgmn	4026.793	580.3381	3258.135	655.6059	815.7981	651.2599	-1.88897	0.001807
173	ENSMUSG00000034858	Fam214a	236.7231	781.6938	185.8252	2233.571	1353.102	870.2059	1.879311	0.00182
174	ENSMUSG00000075010	AW112010	323.4104	466.44	280.8025	1362.54	1070.234	541.0896	1.467662	0.001844
175	ENSMUSG00000030109	Slc6a12	465.1109	752.5413	545.0873	1753.726	1118.351	1324.833	1.249524	0.001851
176	ENSMUSG00000066361	Serpina3c	42.51013	0	16.5178	0	0	0.69728	-6.22988	0.001853
177	ENSMUSG00000106767	Gm42727	4.16766	2.033895	119.754	0.777706	2.916168	2.789122	-4.21073	0.0019
178	ENSMUSG00000072620	Slfn2	171.7076	46.77959	379.9093	60.66104	42.28444	18.82657	-2.27856	0.001903
179	ENSMUSG00000023070	Rgn	311.741	4195.248	1891.288	7012.572	12070.02	9337.282	2.150808	0.001915
180	ENSMUSG00000115391	Gm49123	15.83711	2.033895	24.77669	0	0	0.69728	-5.69788	0.001931
181	ENSMUSG00000041119	Pde9a	47.51133	86.77953	66.07118	171.873	219.4417	168.7419	1.479425	0.001932
182	ENSMUSG00000029161	Cgref1	265.8967	160.6777	247.7669	115.1004	26.24552	25.79937	-2.01164	0.002039
183	ENSMUSG00000030254	Rad18	92.52206	41.35587	94.97733	19.44264	16.03893	24.40481	-1.91353	0.002044
184	ENSMUSG00000020089	Ppa1	1100.262	1203.388	929.126	3150.486	2153.59	1971.212	1.168201	0.002096
185	ENSMUSG00000030342	Cd9	459.2762	126.7795	367.521	139.2093	73.63325	59.26883	-1.80624	0.002112
186	ENSMUSG00000034936	Arl4d	17.50417	124.0676	185.8252	519.5074	561.3624	307.5006	2.113117	0.00212

Table S1. Differentially expressed genes from RNA-seq analysis of db/db mice livers (TAC vs. Veh; p-value < 0.05)										
	Gene_ID	Gene_Name	Veh_1	Veh_2	Veh_3	TAC_1	TAC_2	TAC_3	log2FoldChange	p-value
187	ENSMUSG00000062181	Ces3b	136.6993	790.5073	400.5565	2526.766	1651.28	859.7467	1.922096	0.002137
188	ENSMUSG00000022094	Slc39a14	17150.76	3362.707	10439.25	3361.244	3398.794	2942.523	-1.6736	0.002142
189	ENSMUSG00000045282	Tmem86b	229.2213	462.3722	128.0129	854.6985	906.9284	688.913	1.563468	0.00215
190	ENSMUSG00000029449	Rhof	36.67541	4.067791	57.81228	5.44394	0.729042	2.091841	-3.53926	0.002153
191	ENSMUSG00000030122	Ptms	2250.537	3325.419	2192.737	10883.99	4728.567	5022.511	1.408671	0.00216
192	ENSMUSG00000044254	Pcsk9	3427.484	536.9484	2023.43	41.99611	281.4103	736.3281	-2.49803	0.002164
193	ENSMUSG00000010067	Rassf1	170.0405	41.35587	173.4369	24.10888	38.63923	33.46946	-1.98338	0.002167
194	ENSMUSG00000038641	Akr1d1	380.0906	2545.081	1160.375	3907.193	4755.542	5901.781	1.833015	0.002175
195	ENSMUSG00000024646	Cyb5a	4644.441	6816.261	6916.827	26144.13	12608.78	10309.29	1.41687	0.002186
196	ENSMUSG00000086241	4930483K19Rik	5.001192	0.677965	82.58898	0	1.458084	1.394561	-4.86617	0.002217
197	ENSMUSG00000033533	Acsm1	1325.316	2077.285	2134.925	3923.525	4533.184	3773.681	1.144426	0.002227
198	ENSMUSG00000103288	Gm37273	0	0.677965	0	15.55411	4.374253	28.5885	5.133089	0.002236
199	ENSMUSG00000044201	Cdc25c	0.833532	0	74.33008	0.777706	0	0	-6.51705	0.002257
200	ENSMUSG00000075552	Cyp3a41b	3.334128	2.71186	0	1.555411	22.60031	98.31653	4.071592	0.00226
201	ENSMUSG00000078945	Naip2	208.383	105.7626	194.0841	40.44069	77.27846	49.50691	-1.59559	0.00227
202	ENSMUSG00000036278	Macrod1	69.18316	292.203	165.178	836.8113	374.7276	573.1645	1.7576	0.002279
203	ENSMUSG00000041220	Elovl6	542.6294	9136.936	1284.259	175.7615	776.4298	890.427	-2.57345	0.002309
204	ENSMUSG00000039349	C130074G19Rik	966.8972	1069.829	1284.259	2277.122	2197.333	2387.488	1.049668	0.002323
205	ENSMUSG00000041135	Ripk2	301.7386	80.67785	214.7313	62.21645	67.07187	49.50691	-1.7364	0.002371
206	ENSMUSG00000079523	Tmsb10	305.9063	64.40669	557.4756	76.99286	37.18115	96.22469	-2.12827	0.002401
207	ENSMUSG00000077148	Gm22935	32.50775	14.23727	41.29449	0.777706	1.458084	6.972804	-3.21206	0.002406
208	ENSMUSG00000053613	Notumos	0	12.20337	0	63.77186	47.38774	39.74498	3.373748	0.002446
209	ENSMUSG00000053553	3110082I17Rik	78.35201	12.88134	260.1553	18.66494	12.39372	24.40481	-2.6304	0.002475
210	ENSMUSG00000017631	Abr	282.5674	189.8302	177.5663	78.54827	74.3623	101.8029	-1.35944	0.002478
211	ENSMUSG00000034640	Tiparp	981.0672	195.9319	834.1487	262.0868	158.2021	165.2555	-1.77774	0.002485
212	ENSMUSG00000047719	Ubiad1	228.3878	161.3557	301.4498	73.10433	95.50452	95.52741	-1.37273	0.002519
213	ENSMUSG00000033427	Upb1	1156.109	2432.539	1573.32	3212.702	4224.799	5286.083	1.300809	0.002521
214	ENSMUSG00000009772	Nuak2	33.34128	32.54233	4.129449	158.652	67.80092	94.13285	2.075384	0.002541
215	ENSMUSG00000029499	Pxmp2	316.7422	707.7956	276.6731	1328.321	1145.325	1018.727	1.415615	0.002575
216	ENSMUSG00000114196	Gm47547	19.17124	14.23727	66.07118	5.44394	3.645211	4.183682	-2.82144	0.002576
217	ENSMUSG00000068860	Gm128	1.667064	0	66.07118	0.777706	0	0	-6.35987	0.002582

Table S1. Differentially expressed genes from RNA-seq analysis of db/db mice livers (TAC vs. Veh; p-value < 0.05)										
	Gene_ID	Gene_Name	Veh_1	Veh_2	Veh_3	TAC_1	TAC_2	TAC_3	log2FoldChange	p-value
218	ENSMUSG00000015501	Hivep2	689.331	138.9828	858.9254	138.4316	172.0539	163.8609	-1.82456	0.002595
219	ENSMUSG00000073834	Mup11	0	0	0	9.332468	8.748505	16.73473	5.572509	0.002635
220	ENSMUSG00000030382	Slc27a5	4524.412	7549.141	3794.964	9644.328	14106.96	14234.28	1.258575	0.002657
221	ENSMUSG00000111920	Gm7370	2.500596	0	45.42394	0	0	0	-6.81572	0.002663
222	ENSMUSG00000020773	Trim47	75.85142	59.66093	136.2718	24.88658	20.41318	32.77218	-1.76212	0.002671
223	ENSMUSG00000000628	Hk2	42.51013	65.08465	86.71843	18.66494	16.76797	18.12929	-1.82896	0.002692
224	ENSMUSG00000112796	Gm40770	0.833532	5.423721	0	59.88334	18.95509	13.94561	3.563507	0.002727
225	ENSMUSG00000113029	Gm40578	4.16766	0	41.29449	0	0	0	-6.73908	0.002741
226	ENSMUSG00000025260	Hsd17b10	716.004	2239.319	1651.78	8147.245	3802.684	2665.703	1.665808	0.002755
227	ENSMUSG00000000532	Acvr1b	1838.772	187.7963	982.8088	206.8697	201.2156	345.1538	-1.99807	0.002773
228	ENSMUSG00000033831	Fgb	384302.5	20290.14	321671.7	78331.29	43623.69	25899.78	-2.2963	0.002842
229	ENSMUSG00000114638	Gm31834	4.16766	9.491512	49.55339	0.777706	2.187126	0.69728	-4.00421	0.002843
230	ENSMUSG00000073988	Ttpa	4713.624	7246.091	6400.646	11418.27	14799.55	12785.33	1.0871	0.002854
231	ENSMUSG00000028007	Snx7	639.3191	269.1521	668.9707	136.8762	225.274	210.5787	-1.45625	0.002861
232	ENSMUSG00000031391	L1cam	0.833532	4.745756	66.07118	0.777706	1.458084	0	-4.92825	0.00289
233	ENSMUSG00000078995	Zfp456	5.834724	7.457616	45.42394	0	1.458084	1.394561	-4.24469	0.002894
234	ENSMUSG00000043895	S1pr2	369.2547	117.9659	264.2847	19.44264	81.65272	93.43557	-1.947	0.002985
235	ENSMUSG00000039552	Rsph4a	14.17004	32.54233	28.90614	272.9747	39.36827	64.14979	2.330604	0.002987
236	ENSMUSG00000045087	S1pr5	57.51371	71.8643	41.29449	149.3195	198.2995	131.0887	1.450485	0.002994
237	ENSMUSG00000010095	Slc3a2	2213.028	350.508	1292.518	414.5171	388.5794	348.6402	-1.74315	0.003005
238	ENSMUSG00000061724	Gm2423	17.50417	24.40674	57.81228	3.888528	3.645211	7.670084	-2.64142	0.00301
239	ENSMUSG00000064120	Mocs1	988.569	755.9311	607.029	1538.302	1681.9	1886.143	1.114394	0.003018
240	ENSMUSG00000089873	Mup13	0	0	0	3.888528	40.09732	1.394561	5.954305	0.003055
241	ENSMUSG00000040699	Limd2	55.84665	69.15244	24.77669	187.4271	126.8533	150.6126	1.569542	0.003074
242	ENSMUSG00000024990	Rbp4	11664.45	27199.96	19936.98	80991.05	41911.17	35693.09	1.431427	0.003084
243	ENSMUSG00000003053	Cyp2c29	425.1013	6115.923	1891.288	13987.81	8119.342	14509.71	2.117927	0.003099
244	ENSMUSG00000100334	C230024C17Rik	3.334128	21.69488	45.42394	1.555411	0	2.789122	-3.95414	0.003102
245	ENSMUSG00000026270	Capn10	163.3723	183.0506	136.2718	342.1905	363.063	373.7423	1.147661	0.003117
246	ENSMUSG00000020484	Xbp1	14083.36	1109.829	10133.67	3222.812	1009.723	1458.711	-2.15376	0.003136
247	ENSMUSG00000104445	Rhbg	5.834724	23.05081	0	47.44005	61.96858	80.88452	2.518013	0.00316
248	ENSMUSG00000040570	Rundc3b	23.3389	5.423721	90.84788	2.333117	4.374253	6.275523	-3.12639	0.003166

Table S1. Differentially expressed genes from RNA-seq analysis of db/db mice livers (TAC vs. Veh; p-value < 0.05)

	Gene_ID	Gene_Name	Veh_1	Veh_2	Veh_3	TAC_1	TAC_2	TAC_3	log2FoldChange	p-value
249	ENSMUSG00000028194	Ddah1	69.18316	764.0667	210.6019	842.2552	1636.7	2015.838	2.100849	0.003183
250	ENSMUSG00000039632	Ccdc151	2.500596	34.57622	8.258898	196.7595	51.76199	57.87427	2.685211	0.00319
251	ENSMUSG00000108435	Gm45051	5.834724	12.88134	0	175.7615	16.03893	13.94561	3.271173	0.003213
252	ENSMUSG00000028542	Slc6a9	949.393	677.2871	491.4044	212.3136	327.3399	337.4837	-1.27657	0.003251
253	ENSMUSG00000057103	Nat8f1	487.6162	431.8638	825.8898	1227.22	1500.369	1264.169	1.203096	0.003257
254	ENSMUSG00000032946	Rasgrp2	146.7016	202.0336	169.3074	584.057	323.6947	357.0076	1.285757	0.003259
255	ENSMUSG00000026170	Cyp27a1	1226.959	3168.131	1829.346	7202.332	4181.056	4757.544	1.374008	0.0033
256	ENSMUSG00000020642	Rnf144a	193.3794	105.7626	140.4013	13.221	63.42666	44.62594	-1.85728	0.003389
257	ENSMUSG00000021254	Gpatch2l	416.766	166.1015	528.5695	51.32857	160.3893	124.1159	-1.71828	0.00339
258	ENSMUSG00000024899	Papss2	433.4367	1781.014	986.9383	2231.238	3247.154	4007.27	1.566305	0.003438
259	ENSMUSG00000109628	BC024386	515.1228	2019.658	1478.343	6735.709	2566.957	3374.14	1.659787	0.003449
260	ENSMUSG00000025132	Arhgdia	2674.804	512.5416	2151.443	605.8327	484.813	609.4231	-1.64998	0.003455
261	ENSMUSG00000053461	Hhipl2	116.6945	22.37285	94.97733	13.9987	9.477547	25.79937	-2.23384	0.003482
262	ENSMUSG00000034813	Grip1	6.668256	1.35593	111.4951	2.333117	3.645211	2.091841	-3.82018	0.00349
263	ENSMUSG00000048782	Insc	580.9718	103.0507	198.2135	122.8775	59.05241	33.46946	-2.03959	0.003501
264	ENSMUSG00000020255	D10Wsu102e	793.5225	206.7794	462.4983	112.7673	147.9955	216.1569	-1.6169	0.003534
265	ENSMUSG00000028059	Arhgef2	352.5841	107.7965	156.9191	69.99351	61.96858	71.1226	-1.61109	0.003633
266	ENSMUSG00000001525	Tubb5	1585.378	546.4399	1313.165	256.6429	430.1348	543.8787	-1.48365	0.003646
267	ENSMUSG00000005674	Tomm40l	46.67779	178.9828	16.5178	314.1931	276.307	410.6981	2.003861	0.003696
268	ENSMUSG00000021509	Slc25a48	45.84426	159.3218	66.07118	353.8561	224.545	273.3339	1.62939	0.003703
269	ENSMUSG00000020941	Map3k14	96.68972	44.7457	202.343	29.55282	34.26498	28.5885	-1.85755	0.003706
270	ENSMUSG00000038352	Arl5c	4.16766	4.745756	45.42394	0	0.729042	1.394561	-4.54448	0.003709
271	ENSMUSG00000001473	Tubb6	580.1383	148.4744	524.44	137.6539	110.0854	162.4663	-1.60605	0.003784
272	ENSMUSG00000053693	Mast1	37.50894	8.135581	82.58898	4.666234	7.290421	6.972804	-2.69601	0.003788
273	ENSMUSG00000003382	Etv3	682.6627	208.8133	652.4529	141.5424	200.4866	196.6331	-1.51437	0.003807
274	ENSMUSG00000032418	Me1	1889.617	9228.461	2139.055	854.6985	1347.27	1656.738	-1.78129	0.00382
275	ENSMUSG00000020122	Egfr	11068.47	928.1342	7399.972	1580.298	2320.541	668.6919	-2.08558	0.00382
276	ENSMUSG00000029776	Hibadh	643.4867	2308.471	1226.446	4500.583	3483.363	3415.279	1.446961	0.003822
277	ENSMUSG00000040612	Ildr2	890.2122	462.3722	557.4756	1469.086	1215.313	1709.034	1.199579	0.003835
278	ENSMUSG00000053175	Bcl3	1652.061	98.98291	821.7603	311.86	142.8923	86.46277	-2.24938	0.003847
279	ENSMUSG00000086255	Gm11534	2.500596	4.067791	33.03559	0.777706	0	0	-5.53605	0.003885

Table S1. Differentially expressed genes from RNA-seq analysis of db/db mice livers (TAC vs. Veh; p-value < 0.05)

	Gene_ID	Gene_Name	Veh_1	Veh_2	Veh_3	TAC_1	TAC_2	TAC_3	log2FoldChange	p-value
280	ENSMUSG00000003555	Cyp17a1	2878.186	1979.658	2064.724	1067.012	1198.545	438.5894	-1.35664	0.00391
281	ENSMUSG00000098284	A330093E20Rik	2.500596	6.779651	0	48.21775	21.87126	20.91841	2.993976	0.003963
282	ENSMUSG00000045038	Prkce	373.4224	317.9656	470.7572	163.3182	182.9896	183.3847	-1.12532	0.004046
283	ENSMUSG00000022214	Dcaf11	1206.121	1865.76	1081.916	2523.655	3166.959	3392.966	1.126453	0.004065
284	ENSMUSG00000005397	Nid1	721.8388	350.508	557.4756	27.9974	189.5509	220.3406	-1.89509	0.004076
285	ENSMUSG00000040213	Kyat3	556.7994	722.0328	540.9578	891.2507	1427.464	1957.266	1.2303	0.004077
286	ENSMUSG00000017144	Rnd3	436.7708	246.7793	491.4044	157.0965	152.3698	181.9902	-1.2494	0.004148
287	ENSMUSG00000023034	Nr4a1	142.534	27.1186	239.508	31.88593	32.80689	32.0749	-2.05472	0.004157
288	ENSMUSG00000057933	Gsta2	14.17004	256.2708	289.0614	321.9701	948.4838	1564	2.350106	0.004159
289	ENSMUSG00000021789	Sftpa1	70.01669	35.25419	57.81228	204.5366	114.4596	142.2452	1.510124	0.004171
290	ENSMUSG00000080971	Gm16128	2.500596	2.033895	37.16504	0.777706	0	0	-5.61034	0.0042
291	ENSMUSG00000056708	Ier5	149.2022	46.10163	185.8252	39.66299	39.36827	31.37762	-1.76534	0.004214
292	ENSMUSG00000073481	45353	1506.192	3612.198	2052.336	5081.529	6099.166	5723.277	1.236365	0.004242
293	ENSMUSG00000057335	Cep170	436.7708	168.1353	231.2491	59.88334	122.4791	108.7757	-1.5256	0.004247
294	ENSMUSG00000096883	Shisa8	0	2.033895	0	45.88463	18.22605	2.091841	4.491744	0.004283
295	ENSMUSG00000024235	Map3k8	275.8991	32.54233	128.0129	47.44005	29.89073	26.49665	-2.07484	0.004353
296	ENSMUSG00000022003	Slc25a30	685.9969	458.3044	858.9254	374.0764	253.7067	168.0446	-1.32762	0.004387
297	ENSMUSG00000037826	Ppm1k	302.5721	733.5582	557.4756	1053.013	1758.45	1156.091	1.31655	0.004413
298	ENSMUSG00000035873	Pawr	456.7756	105.7626	239.508	61.43875	118.8339	64.14979	-1.71584	0.004428
299	ENSMUSG00000036944	Tmem71	115.861	39.99994	247.7669	37.32987	40.09732	26.49665	-1.92621	0.004449
300	ENSMUSG00000017453	Pipox	766.016	2187.115	1296.647	3875.307	3233.302	3366.47	1.300686	0.00453
301	ENSMUSG00000036892	Prodh2	161.7052	1518.642	660.7118	4572.909	2197.333	2038.848	1.910593	0.004564
302	ENSMUSG00000018861	Fdxr	124.1963	125.4235	111.4951	540.5054	170.5959	293.555	1.471194	0.004566
303	ENSMUSG00000089782	Gm3531	29.17362	22.37285	313.8381	26.44199	19.68414	13.94561	-2.56869	0.004587
304	ENSMUSG00000022911	Arl13b	40.00954	45.42366	20.64724	106.5457	116.6467	95.52741	1.518564	0.004615
305	ENSMUSG00000000567	Sox9	385.0918	11.52541	284.932	70.77122	21.87126	23.01025	-2.55656	0.004619
306	ENSMUSG00000102919	Gm37726	0.833532	4.745756	24.77669	0	0	0	-6.12985	0.004652
307	ENSMUSG00000037953	A4gnt	2.500596	129.4913	94.97733	0	0.729042	16.03745	-3.74804	0.004658
308	ENSMUSG00000028637	Ccdc30	15.00358	26.44064	16.5178	99.54633	59.05241	51.59875	1.824608	0.004686
309	ENSMUSG00000023927	Satb1	78.35201	18.30506	45.42394	13.9987	8.019463	7.670084	-2.262	0.004686
310	ENSMUSG00000020419	Hormad2	10.83592	86.10157	45.42394	13.9987	0.729042	1.394561	-3.15528	0.004723

Table S1. Differentially expressed genes from RNA-seq analysis of db/db mice livers (TAC vs. Veh; p-value < 0.05)										
	Gene_ID	Gene_Name	Veh_1	Veh_2	Veh_3	TAC_1	TAC_2	TAC_3	log2FoldChange	p-value
311	ENSMUSG00000032207	Lipc	173.3747	892.88	289.0614	1084.122	1570.357	1651.857	1.66226	0.004739
312	ENSMUSG00000022613	Miox	42.51013	0	41.29449	0.777706	0	2.789122	-4.52416	0.004769
313	ENSMUSG00000029864	Gstk1	316.7422	1109.829	479.0161	2088.14	1767.927	1384.799	1.455954	0.004795
314	ENSMUSG00000104309	Gm5846	8.335321	8.135581	99.10677	2.333117	3.645211	6.275523	-3.14993	0.00485
315	ENSMUSG00000109764	Klkb1	284.2344	1245.422	503.7928	3124.044	1595.144	1552.146	1.621953	0.004867
316	ENSMUSG00000043681	Fam25c	14.17004	32.54233	33.03559	362.4108	69.98804	18.12929	2.518749	0.00489
317	ENSMUSG00000032878	Ccdc85a	0.833532	0	57.81228	0.777706	0	0	-6.14784	0.00491
318	ENSMUSG00000045538	Ddx28	1196.952	149.8303	664.8413	171.873	235.4806	175.7147	-1.78637	0.004951
319	ENSMUSG00000085024	C230035I16Rik	5.001192	2.71186	115.6246	3.110823	3.645211	3.486402	-3.51776	0.004969
320	ENSMUSG00000042589	Cux2	0.833532	8.135581	20.64724	88.65845	18.22605	65.54436	2.772728	0.00499
321	ENSMUSG00000047109	Cldn14	275.0656	75.25413	276.6731	90.99156	51.76199	51.59875	-1.68017	0.005055
322	ENSMUSG00000040883	Tmem205	950.2265	2406.776	1581.579	5054.309	3742.902	3144.037	1.273376	0.005076
323	ENSMUSG00000097750	Gm4673	174.2082	59.66093	227.1197	44.32922	33.53594	62.05795	-1.69987	0.005093
324	ENSMUSG00000019822	Smpd2	175.0417	322.0334	66.07118	1076.345	484.813	347.2456	1.741111	0.005119
325	ENSMUSG00000023942	Slc29a1	449.2738	1459.659	516.1811	2846.403	2278.986	1649.765	1.478342	0.005146
326	ENSMUSG00000032519	Slc25a38	119.1951	173.5591	107.3657	508.6195	239.8549	279.6094	1.347672	0.005231
327	ENSMUSG00000031845	Bco1	123.3627	84.74564	165.178	35.77446	45.20061	54.38787	-1.44024	0.005245
328	ENSMUSG00000082141	Gm11212	48.34486	0	189.9547	10.11017	1.458084	7.670084	-3.60694	0.005297
329	ENSMUSG00000093858	Gm19967	2.500596	0	49.55339	0.777706	0	0	-5.96658	0.005317
330	ENSMUSG00000030591	Psmd8	2521.434	1547.116	2609.812	928.5806	1192.713	1041.737	-1.07693	0.005375
331	ENSMUSG00000052837	Junb	1732.913	74.57616	1280.129	306.416	188.8219	198.0276	-2.15408	0.005387
332	ENSMUSG00000079563	Pglyrp2	2793.999	2009.489	1849.993	582.5015	1175.945	1148.421	-1.19541	0.005422
333	ENSMUSG00000059430	Actg2	0.833532	0	70.20063	0.777706	0	0.69728	-5.52792	0.00545
334	ENSMUSG00000025936	Gm4956	4.16766	21.01692	0	35.77446	78.73655	53.69059	2.536214	0.005463
335	ENSMUSG00000079012	Serpina3m	24556.69	5551.178	22319.67	4948.541	7725.659	5634.025	-1.5177	0.005476
336	ENSMUSG00000021922	Itih4	278698.9	16943.7	145327.7	51036.93	35677.86	21803.96	-2.02273	0.005507
337	ENSMUSG00000108105	Gm5340	43.34367	0	61.94173	1.555411	4.374253	1.394561	-3.81518	0.005526
338	ENSMUSG00000017868	Sgk2	120.0286	326.7792	82.58898	360.8554	526.3684	726.5662	1.587719	0.005566
339	ENSMUSG00000057880	Abat	766.8495	3191.182	1416.401	2277.122	7096.496	7896.003	1.683252	0.005631
340	ENSMUSG00000042770	Hebp1	469.2785	1267.795	1015.844	2536.098	2004.137	2050.702	1.260518	0.005643
341	ENSMUSG00000026773	Pfkfb3	1297.809	97.62698	1185.152	222.4238	283.5974	131.0887	-2.01615	0.00569

Table S1. Differentially expressed genes from RNA-seq analysis of db/db mice livers (TAC vs. Veh; p-value < 0.05)										
	Gene_ID	Gene_Name	Veh_1	Veh_2	Veh_3	TAC_1	TAC_2	TAC_3	log2FoldChange	p-value
342	ENSMUSG00000034201	Gas2l1	854.3704	211.5251	479.0161	171.0952	191.7381	191.7521	-1.47859	0.005692
343	ENSMUSG00000032014	Oaf	1179.448	1644.743	1445.307	2715.748	2946.788	2665.703	0.964045	0.005716
344	ENSMUSG00000013698	Pea15a	215.0513	219.6607	297.3203	46.66234	128.3114	100.4084	-1.40204	0.005751
345	ENSMUSG00000024206	Rfx2	17.50417	20.33895	82.58898	4.666234	5.103295	10.45921	-2.49131	0.005764
346	ENSMUSG00000026389	Steap3	157.5376	132.8812	194.0841	328.1918	322.2366	375.8341	1.099504	0.005795
347	ENSMUSG00000027520	Zdbf2	13.33651	4.067791	12.38835	0.777706	0	0	-5.21356	0.005871
348	ENSMUSG00000023707	Ogfod2	120.0286	161.3557	57.81228	495.3985	233.2935	238.4699	1.480878	0.006075
349	ENSMUSG00000082990	Gm14052	1.667064	0.677965	28.90614	0	0	0	-6.16171	0.006157
350	ENSMUSG00000042763	Maneal	1.667064	0	37.16504	0	0	0	-6.50437	0.006174
351	ENSMUSG00000097385	Gm26814	1.667064	0	37.16504	0	0	0	-6.50437	0.006174
352	ENSMUSG00000029059	Fam213b	118.3616	78.64395	128.0129	579.3907	237.6677	147.8234	1.580634	0.006269
353	ENSMUSG00000022550	Adck5	120.8621	92.20325	210.6019	394.2968	302.5525	301.2251	1.270867	0.006294
354	ENSMUSG00000018659	Pnpo	1127.769	1445.422	1069.527	2547.764	1983.724	2742.404	0.995998	0.006294
355	ENSMUSG00000106831	Ube2n-ps1	25.83949	11.52541	70.20063	2.333117	8.748505	5.578243	-2.61542	0.006309
356	ENSMUSG00000033860	Fgg	323022.8	16853.53	258288.8	78584.82	35840.44	20623.46	-2.14705	0.006333
357	ENSMUSG00000018599	Mief2	125.0298	164.7455	49.55339	379.5204	239.8549	306.8034	1.410952	0.006368
358	ENSMUSG00000073492	Gm10521	4.16766	2.71186	53.68284	0.777706	0.729042	2.091841	-3.95012	0.0064
359	ENSMUSG00000026012	Cd28	132.5316	0	78.45953	13.221	2.187126	0.69728	-3.71507	0.006424
360	ENSMUSG00000015709	Arnt2	5.834724	0	28.90614	0	0	0	-6.34919	0.00648
361	ENSMUSG00000028919	Arhgef19	27.50656	305.0843	24.77669	591.0563	374.7276	674.2701	2.174595	0.00649
362	ENSMUSG00000027048	Abcb11	450.9408	2397.285	1073.657	2437.33	4529.539	4890.725	1.595145	0.006492
363	ENSMUSG00000034837	Gnat1	911.0505	254.9149	681.3591	307.9714	184.4477	177.8065	-1.46132	0.006494
364	ENSMUSG00000009281	Rarres2	707.6687	1789.15	1081.916	3316.915	2811.915	2246.637	1.225652	0.006521
365	ENSMUSG00000020108	Ddit4	302.5721	181.0167	342.7443	948.0232	953.5871	315.1707	1.430897	0.006528
366	ENSMUSG00000049562	Ap5b1	194.213	38.64401	86.71843	20.22035	24.05839	41.83682	-1.89676	0.006535
367	ENSMUSG00000106619	Gm9353	3.334128	3.389826	78.45953	2.333117	2.916168	0	-3.94953	0.006571
368	ENSMUSG00000115324	AC107711.1	17.50417	0	33.03559	0	0.729042	0.69728	-5.07424	0.006577
369	ENSMUSG00000035232	Pdk3	30.84069	10.16948	66.07118	1.555411	10.20659	3.486402	-2.75191	0.006627
370	ENSMUSG00000105977	Gm43555	0	1.35593	86.71843	0.777706	0	2.091841	-4.87457	0.00676
371	ENSMUSG00000099206	Gm27942	0	2.71186	33.03559	0	0	0	-6.3816	0.006769
372	ENSMUSG00000005268	Prlr	202.5483	1311.862	466.6277	1477.641	2339.496	2457.913	1.660414	0.00679

Table S1. Differentially expressed genes from RNA-seq analysis of db/db mice livers (TAC vs. Veh; p-value < 0.05)										
	Gene_ID	Gene_Name	Veh_1	Veh_2	Veh_3	TAC_1	TAC_2	TAC_3	log2FoldChange	p-value
373	ENSMUSG00000086688	Gm11560	25.00596	17.62709	218.8608	8.554762	19.68414	16.73473	-2.48984	0.006806
374	ENSMUSG00000025815	Dhtkd1	185.8776	809.4903	247.7669	1071.678	1095.021	1470.564	1.541766	0.006826
375	ENSMUSG00000102282	A930032L01Rik	0	1.35593	74.33008	1.555411	0.729042	0	-5.00373	0.006953
376	ENSMUSG00000022445	Cyp2d26	7278.402	11492.86	6012.478	19000.9	15441.84	17444.56	1.065492	0.006982
377	ENSMUSG00000030340	Scnn1a	66.68256	146.4405	119.754	326.6364	212.1513	276.8203	1.29753	0.006989
378	ENSMUSG00000026058	Khdrbs2	0	0	49.55339	0	0	0	-6.8806	0.007049
379	ENSMUSG00000030450	Oca2	0	0	49.55339	0	0	0	-6.8806	0.007049
380	ENSMUSG00000042761	Mrap2	0	0	49.55339	0	0	0	-6.8806	0.007049
381	ENSMUSG00000072709	Olfr380	0	0	49.55339	0	0	0	-6.8806	0.007049
382	ENSMUSG00000084391	Gm13967	0	0	49.55339	0	0	0	-6.8806	0.007049
383	ENSMUSG00000084555	Gm25270	0	0	49.55339	0	0	0	-6.8806	0.007049
384	ENSMUSG00000094529	Gm21744	0	0	49.55339	0	0	0	-6.8806	0.007049
385	ENSMUSG00000102100	Gm18351	0	0	49.55339	0	0	0	-6.8806	0.007049
386	ENSMUSG00000102140	Gm38058	0	0	49.55339	0	0	0	-6.8806	0.007049
387	ENSMUSG00000104994	Gm42989	0	0	49.55339	0	0	0	-6.8806	0.007049
388	ENSMUSG00000106834	Gm10459	0	0	49.55339	0	0	0	-6.8806	0.007049
389	ENSMUSG00000066072	Cyp4a10	3100.739	17218.96	6669.06	18045.88	31189.15	31576.34	1.581989	0.007065
390	ENSMUSG00000045502	Hcar2	74.18435	10.84744	0	3.110823	0	2.789122	-3.88882	0.007077
391	ENSMUSG00000078650	G6pc	550.1312	6823.041	1668.297	14903.17	9382.043	9616.194	1.90598	0.007091
392	ENSMUSG00000041237	Pklr	1076.09	12332.86	1544.414	482.9552	754.5586	2060.464	-2.18127	0.007104
393	ENSMUSG00000030852	Tacc2	2914.028	646.1007	1123.21	595.7225	640.828	412.79	-1.50682	0.007126
394	ENSMUSG00000029267	Mtf2	230.8884	99.66087	487.275	101.8794	83.1108	84.37093	-1.58328	0.00715
395	ENSMUSG00000022025	Cnmd	0	0.677965	0	29.55282	10.93563	1.394561	4.920693	0.007174
396	ENSMUSG00000024539	Ptpn2	530.9599	137.6269	710.2652	216.2022	129.0405	89.94917	-1.65689	0.007237
397	ENSMUSG00000030359	Pzp	154892.8	53053.48	94287.71	34544.13	52706.1	38908.25	-1.26043	0.007246
398	ENSMUSG00000031445	Proz	2831.508	2913.216	1936.712	6229.422	4529.539	4685.724	1.006138	0.00732
399	ENSMUSG00000028519	Dab1	54.17958	3.389826	115.6246	6.999351	2.916168	13.94561	-2.81946	0.007338
400	ENSMUSG00000062991	Nrg1	83.35321	16.27116	49.55339	3.110823	6.561379	18.12929	-2.41673	0.007422
401	ENSMUSG00000030077	Chl1	2.500596	0	33.03559	0	0	0	-6.37354	0.007451
402	ENSMUSG00000032028	Nxpe2	27.50656	159.3218	41.29449	361.6331	328.798	142.9425	1.840667	0.007479
403	ENSMUSG00000083610	Oaz2-ps	1.667064	0.677965	57.81228	0	0.729042	1.394561	-4.71373	0.007539

Table S1. Differentially expressed genes from RNA-seq analysis of db/db mice livers (TAC vs. Veh; p-value < 0.05)										
	Gene_ID	Gene_Name	Veh_1	Veh_2	Veh_3	TAC_1	TAC_2	TAC_3	log2FoldChange	p-value
404	ENSMUSG00000036199	Ndufa13	231.7219	422.3723	433.5921	1452.754	718.1065	559.9161	1.33238	0.007597
405	ENSMUSG00000023045	Soat2	2887.355	558.6432	2560.258	1043.681	339.0046	492.9772	-1.6783	0.007622
406	ENSMUSG00000049580	Tsku	1567.874	1141.693	1081.916	7743.615	1181.777	2727.064	1.619121	0.007647
407	ENSMUSG00000086443	4933421A08Rik	61.68137	16.94913	623.5468	71.54892	18.95509	23.01025	-2.61296	0.007685
408	ENSMUSG00000034780	B3galt1	2472.256	230.5081	1697.204	488.3992	511.7876	229.4052	-1.83877	0.007728
409	ENSMUSG00000079014	Serpina3i	922.72	0.677965	978.6794	0	9.477547	0	-7.64807	0.007734
410	ENSMUSG00000027499	Pkia	6.668256	18.98302	123.8835	1.555411	8.019463	9.761925	-2.88494	0.007736
411	ENSMUSG00000086784	Isoc2a	502.6198	1305.761	487.275	2648.088	1488.704	1722.98	1.347536	0.007765
412	ENSMUSG00000026664	Phyh	6298.168	12859.64	8622.289	18315.75	20416.1	19107.57	1.057872	0.007845
413	ENSMUSG00000004885	Crabp2	0.833532	0	37.16504	0	0	0	-6.47399	0.007877
414	ENSMUSG00000029650	Slc46a3	269.2309	660.338	194.0841	930.9137	920.0511	1038.25	1.35116	0.00789
415	ENSMUSG00000019876	Pkib	12.50298	12.88134	140.4013	1.555411	10.93563	9.761925	-2.83425	0.007907
416	ENSMUSG00000027381	Bcl2l11	610.1455	109.1524	516.1811	202.9812	81.65272	87.85733	-1.72667	0.007967
417	ENSMUSG00000057899	Adgrf2	0	0.677965	37.16504	0	0	0	-6.46746	0.007974
418	ENSMUSG00000103839	Gm37607	0	7.457616	90.84788	0	2.187126	2.789122	-4.23938	0.008005
419	ENSMUSG00000030895	Hpx	491969	42076.55	348732	132809.6	63449.99	26231.69	-1.98829	0.008012
420	ENSMUSG00000022636	Alcam	735.1753	436.6095	722.6536	212.3136	198.2995	398.8444	-1.22387	0.008046
421	ENSMUSG00000055159	4930583K01Rik	0.833532	0.677965	28.90614	0	0	0	-6.12315	0.00809
422	ENSMUSG00000026608	Kctd3	717.6711	139.6608	433.5921	152.4303	139.247	156.8881	-1.52438	0.008119
423	ENSMUSG00000033429	Mcee	105.025	319.3216	251.8964	1037.459	476.0645	382.8069	1.490312	0.008151
424	ENSMUSG00000025068	Gsto1	305.9063	529.4907	520.3106	822.0349	952.129	1045.223	1.060555	0.0082
425	ENSMUSG00000020182	Ddc	231.7219	498.9823	99.10677	693.7135	713.7322	926.6856	1.475507	0.008222
426	ENSMUSG00000073535	Gm5532	0	5.423721	74.33008	1.555411	0.729042	2.091841	-4.11163	0.008447
427	ENSMUSG00000001227	Sema6b	462.6103	69.83041	235.3786	69.2158	64.88475	101.8029	-1.70316	0.008449
428	ENSMUSG00000046179	E2f8	134.1987	93.55918	173.4369	48.99546	68.52996	29.28578	-1.434	0.008471
429	ENSMUSG00000047030	Spata2	251.7267	162.0337	260.1553	98.76862	102.0659	103.8948	-1.1362	0.00848
430	ENSMUSG00000090298	Sult3a2	11.66945	39.32198	181.6958	430.8489	137.0599	400.9362	2.117882	0.008548
431	ENSMUSG00000030203	Dusp16	1799.596	581.0161	1028.233	448.7362	539.4912	429.5247	-1.26627	0.008578
432	ENSMUSG00000052062	Pard3b	119.1951	80.67785	260.1553	52.10628	48.11678	60.66339	-1.48302	0.008653
433	ENSMUSG00000027247	Arhgap1	450.1073	122.0337	433.5921	133.7654	103.524	129.6942	-1.44756	0.008655
434	ENSMUSG00000030861	Acadsb	413.4319	1075.931	458.3688	1161.115	2025.279	1589.102	1.289409	0.008703

Table S1. Differentially expressed genes from RNA-seq analysis of db/db mice livers (TAC vs. Veh; p-value < 0.05)										
	Gene_ID	Gene_Name	Veh_1	Veh_2	Veh_3	TAC_1	TAC_2	TAC_3	log2FoldChange	p-value
435	ENSMUSG00000024292	Cyp4f14	55.84665	742.3718	115.6246	541.2831	1762.095	1564	2.073578	0.008712
436	ENSMUSG00000022218	Tgm1	610.979	29.83046	338.6148	111.2119	51.76199	69.03076	-2.07725	0.008729
437	ENSMUSG00000032363	Adamts7	70.85022	54.23721	24.77669	156.3188	82.38176	215.4596	1.547377	0.008812
438	ENSMUSG00000044005	Gls2	933.5559	2661.691	1412.272	3110.045	4794.91	3936.845	1.240668	0.00882
439	ENSMUSG00000038422	Hdhd3	33.34128	58.98296	74.33008	246.5327	99.14973	119.2349	1.514937	0.008839
440	ENSMUSG00000026782	Abi2	138.3663	70.50837	128.0129	30.33052	50.30391	44.62594	-1.4162	0.008872
441	ENSMUSG00000048911	Rnf24	282.5674	154.576	227.1197	94.10239	69.98804	117.8404	-1.23437	0.008892
442	ENSMUSG00000003809	Gcdh	1721.244	5922.025	2890.614	8071.807	8921.288	8592.586	1.279756	0.008908
443	ENSMUSG00000079108	Srp54c	95.02265	104.4066	338.6148	43.55152	75.09134	55.08515	-1.60054	0.008917
444	ENSMUSG00000003355	Fkbp11	150.0358	65.76262	322.097	80.10368	44.47157	41.83682	-1.66829	0.008926
445	ENSMUSG00000038600	Atp6v0a4	2.500596	4.067791	16.5178	0	0	0	-5.7372	0.009028
446	ENSMUSG00000034108	Ccs	693.4987	974.9138	1152.116	2602.203	1652.738	1632.333	1.064692	0.009039
447	ENSMUSG00000020917	Acly	1491.189	13840.66	1639.391	209.9805	1340.708	2077.896	-2.22597	0.009064
448	ENSMUSG00000063804	Lin28b	0	0	45.42394	0	0	0	-6.75232	0.009206
449	ENSMUSG00000091908	Gm17231	0	0	45.42394	0	0	0	-6.75232	0.009206
450	ENSMUSG00000097248	Gm2694	0	0	45.42394	0	0	0	-6.75232	0.009206
451	ENSMUSG00000107439	Gm45060	0	0	45.42394	0	0	0	-6.75232	0.009206
452	ENSMUSG00000108359	C430039J01Rik	0	0	45.42394	0	0	0	-6.75232	0.009206
453	ENSMUSG00000108644	Gm44920	0	0	45.42394	0	0	0	-6.75232	0.009206
454	ENSMUSG00000110719	Gm2716	0	0	45.42394	0	0	0	-6.75232	0.009206
455	ENSMUSG00000112410	B230217J21Rik	0	0	45.42394	0	0	0	-6.75232	0.009206
456	ENSMUSG00000087330	Bloodlinc	3.334128	1.35593	45.42394	0.777706	0	1.394561	-4.41059	0.009215
457	ENSMUSG00000064796	Terc	0.833532	4.067791	28.90614	0	0	0.69728	-5.30681	0.009282
458	ENSMUSG00000053279	Aldh1a1	2896.524	22945.73	6896.18	27070.38	34040.43	42223.81	1.658062	0.009363
459	ENSMUSG00000038301	Snx10	1652.061	480.6773	1123.21	430.0712	542.4073	324.9327	-1.32701	0.009375
460	ENSMUSG00000087141	Plcxd2	183.3771	347.1181	189.9547	314.9708	1286.03	465.086	1.513993	0.009411
461	ENSMUSG00000037797	Adh4	255.0608	555.9314	450.1099	487.6215	1407.78	1322.741	1.352656	0.009429
462	ENSMUSG00000046814	Gchfr	479.2809	733.5582	615.2879	2389.89	1018.472	933.6584	1.247911	0.009658
463	ENSMUSG00000021306	Gpr137b	15.00358	43.38977	94.97733	7.777057	12.39372	13.94561	-2.1086	0.009727
464	ENSMUSG00000071064	Zfp827	3.334128	4.745756	94.97733	3.110823	0.729042	5.578243	-3.37224	0.009808
465	ENSMUSG00000093912	Mir3470b	0.833532	4.745756	74.33008	0.777706	2.916168	2.091841	-3.69609	0.009808

Table S1. Differentially expressed genes from RNA-seq analysis of db/db mice livers (TAC vs. Veh; p-value < 0.05)										
	Gene_ID	Gene_Name	Veh_1	Veh_2	Veh_3	TAC_1	TAC_2	TAC_3	log2FoldChange	p-value
466	ENSMUSG00000028470	Hint2	398.4283	548.4738	578.1228	2191.575	788.0945	791.4132	1.308252	0.009843
467	ENSMUSG00000033581	Igf2bp2	113.3604	102.3727	90.84788	28.77511	33.53594	56.47971	-1.37415	0.009849
468	ENSMUSG00000038400	Pmepa1	155.8705	20.33895	260.1553	56.77251	21.87126	24.40481	-2.06357	0.009887
469	ENSMUSG00000042364	Snx18	554.2988	295.5928	512.0517	188.9825	245.6872	197.3303	-1.10385	0.009913
470	ENSMUSG00000042349	Ikbke	1270.303	92.88122	945.6438	217.7576	215.7965	221.0379	-1.81693	0.009922
471	ENSMUSG00000047728	BC025446	560.9671	1144.405	429.4627	2763.188	1465.375	1177.009	1.335461	0.009933
472	ENSMUSG00000056204	Pgpep1	426.7684	333.5588	272.5436	706.1567	678.7382	659.6272	0.976447	0.009963
473	ENSMUSG00000027122	Arl14ep	283.4009	189.1523	693.7474	104.9903	165.4926	158.9799	-1.4261	0.009965
474	ENSMUSG00000070304	Scn2b	0	0.677965	45.42394	0.777706	0	0	-5.78824	0.009974
475	ENSMUSG00000055862	Izumo4	12.50298	40.67791	24.77669	185.0939	41.5554	70.42532	1.919118	0.010021
476	ENSMUSG00000037887	Dusp8	110.0262	29.83046	53.68284	20.22035	22.60031	5.578243	-2.00933	0.01005
477	ENSMUSG00000032561	Acpp	1091.093	73.8982	656.5824	195.9818	150.9117	159.6772	-1.84555	0.01007
478	ENSMUSG00000052974	Cyp2f2	781.8531	3255.588	1664.168	3373.687	6129.786	5423.447	1.387859	0.010089
479	ENSMUSG00000116498	Gm38563	0	2.71186	28.90614	0	0	0	-6.20075	0.010121
480	ENSMUSG00000089941	Gm16168	0.833532	4.067791	103.2362	5.44394	0	1.394561	-3.93635	0.01014
481	ENSMUSG00000030731	Syt3	15.00358	58.305	0	166.429	83.83984	96.92197	2.135116	0.010168
482	ENSMUSG00000010154	Spire2	18.33771	9.491512	24.77669	3.110823	2.187126	2.091841	-2.78462	0.0102
483	ENSMUSG00000086748	Gm13261	13.33651	4.067791	37.16504	2.333117	2.187126	0	-3.52256	0.010247
484	ENSMUSG00000002588	Pon1	1017.743	7499.65	3497.643	9115.488	14308.91	12171.03	1.566607	0.010264
485	ENSMUSG00000024845	Tmem134	347.5829	537.6263	429.4627	1280.881	725.3969	779.5595	1.082459	0.010264
486	ENSMUSG00000052133	Sema5b	20.8383	60.33889	0	107.3234	118.1048	124.8132	1.999708	0.010368
487	ENSMUSG00000023829	Slc22a1	1014.409	2998.64	1527.896	3435.126	4851.775	4637.612	1.220861	0.010372
488	ENSMUSG00000001666	Ddt	243.3914	454.9146	371.6504	1096.565	740.7068	549.4569	1.158186	0.010397
489	ENSMUSG00000022474	Pmm1	39.17601	75.93209	49.55339	195.2041	96.9626	141.5479	1.383089	0.010409
490	ENSMUSG00000097011	Gm4651	1.667064	2.033895	28.90614	0	0.729042	0	-5.24285	0.010421
491	ENSMUSG00000110960	Supt4b	3.334128	2.71186	41.29449	0.777706	0.729042	1.394561	-3.88887	0.010425
492	ENSMUSG00000078572	Ndufaf8	50.01192	46.77959	16.5178	167.2067	94.04643	83.67365	1.53353	0.010499
493	ENSMUSG00000024338	Psmb8	209.2165	96.94901	107.3657	643.9403	284.3264	207.7896	1.448167	0.010513
494	ENSMUSG00000015890	Amdhd1	967.7307	1189.829	1135.598	2746.856	1687.003	1985.157	0.963268	0.010545
495	ENSMUSG00000052557	Gan	861.0386	187.1184	532.6989	140.7647	174.2411	256.5992	-1.46683	0.010571
496	ENSMUSG00000004677	Myo9b	1141.939	559.3212	739.1714	351.523	326.6109	462.2969	-1.09856	0.010574

Table S1. Differentially expressed genes from RNA-seq analysis of db/db mice livers (TAC vs. Veh; p-value < 0.05)										
	Gene_ID	Gene_Name	Veh_1	Veh_2	Veh_3	TAC_1	TAC_2	TAC_3	log2FoldChange	p-value
497	ENSMUSG00000036832	Lpar3	24.17243	9.491512	24.77669	0.777706	0	4.880963	-3.3321	0.010596
498	ENSMUSG00000017002	Slpi	186.7112	90.84732	198.2135	74.65974	27.7036	67.6362	-1.47394	0.010611
499	ENSMUSG00000020532	Acaca	833.5321	3717.283	900.2199	227.8678	538.7621	915.5291	-1.69759	0.010696
500	ENSMUSG00000029352	Crybb3	225.0537	3.389826	189.9547	48.21775	3.645211	4.183682	-2.89789	0.01071
501	ENSMUSG00000021281	Tnfaip2	751.0124	130.8473	346.8737	146.9864	120.2919	157.5854	-1.53384	0.010722
502	ENSMUSG00000111147	Gm33699	85.02027	14.23727	70.20063	17.88723	13.8518	2.789122	-2.28496	0.010722
503	ENSMUSG00000047767	Atg16l2	629.3167	91.52529	561.6051	178.0946	130.4985	99.01381	-1.64961	0.010752
504	ENSMUSG00000031377	Bmx	0.833532	8.135581	28.90614	0	0.729042	0.69728	-4.60769	0.010764
505	ENSMUSG00000021646	Mccc2	239.2237	611.5245	280.8025	661.8275	996.6006	1014.543	1.233332	0.01081
506	ENSMUSG00000107605	Gm44117	0.833532	1.35593	0	11.66559	17.49701	9.761925	3.668599	0.010814
507	ENSMUSG00000025192	Entpd7	51.67899	18.30506	206.4724	17.88723	26.24552	18.82657	-2.08719	0.01084
508	ENSMUSG00000039438	Ttc36	321.7434	545.7619	681.3591	2068.697	804.1334	911.3455	1.29384	0.010851
509	ENSMUSG00000028464	Tpm2	5.001192	33.89826	33.03559	103.4349	58.32337	96.92197	1.895715	0.010955
510	ENSMUSG00000053965	Pde5a	60.01431	35.93215	61.94173	17.10952	16.03893	18.12929	-1.60277	0.010987
511	ENSMUSG00000028262	Clca3a2	3.334128	2.71186	70.20063	1.555411	3.645211	0	-3.79533	0.010991
512	ENSMUSG00000002881	Nab1	2138.01	887.4563	2072.983	484.5106	863.9149	852.0766	-1.21076	0.011002
513	ENSMUSG00000028737	Aldh4a1	131.6981	1292.879	355.1326	890.473	2534.879	2927.183	1.832489	0.011008
514	ENSMUSG00000105804	Gm43654	2.500596	3.389826	53.68284	0	0	2.789122	-4.30856	0.011014
515	ENSMUSG00000049353	Rd3	0	1.35593	0	17.88723	8.748505	10.45921	4.095366	0.011086
516	ENSMUSG00000040613	Apobec1	495.118	203.3895	429.4627	179.65	152.3698	161.769	-1.18752	0.011115
517	ENSMUSG00000112006	Gm48633	103.358	145.7625	189.9547	530.3953	275.5779	234.2862	1.261213	0.011174
518	ENSMUSG00000042265	Trem1	15.83711	0	12.38835	0	0	0	-6.10428	0.011212
519	ENSMUSG00000091573	Serpina3d-ps	15.83711	0	12.38835	0	0	0	-6.10428	0.011212
520	ENSMUSG00000116284	Gm3787	0	2.71186	0	6.999351	29.16168	14.64289	3.774767	0.01122
521	ENSMUSG00000031919	Tmed6	0.833532	0	33.03559	0	0	0	-6.30316	0.011228
522	ENSMUSG00000029228	Lnx1	20.00477	2.033895	53.68284	0.777706	0.729042	5.578243	-3.34004	0.011244
523	ENSMUSG00000028051	Hcn3	35.00835	1303.727	202.343	93.32468	39.36827	175.0174	-2.32752	0.011257
524	ENSMUSG00000046324	Ermp1	1365.326	1222.371	962.1616	496.1762	601.4597	732.8417	-0.95783	0.011372
525	ENSMUSG00000096415	Gm24722	0	0.677965	33.03559	0	0	0	-6.2958	0.011379
526	ENSMUSG00000022129	Dct	12.50298	62.37279	107.3657	148.5418	246.4162	183.3847	1.723369	0.01144
527	ENSMUSG00000038195	Rilp	76.68495	191.8641	57.81228	526.5067	182.9896	251.7182	1.53272	0.01151

Table S1. Differentially expressed genes from RNA-seq analysis of db/db mice livers (TAC vs. Veh; p-value < 0.05)										
	Gene_ID	Gene_Name	Veh_1	Veh_2	Veh_3	TAC_1	TAC_2	TAC_3	log2FoldChange	p-value
528	ENSMUSG00000003032	Klf4	104.1915	16.27116	132.1424	27.9974	23.32935	9.064645	-2.04168	0.011579
529	ENSMUSG00000115829	Gm48960	0	2.033895	28.90614	0	0	0	-6.16727	0.011584
530	ENSMUSG00000073682	Gm10563	1.667064	0.677965	235.3786	2.333117	1.458084	0	-5.96608	0.011624
531	ENSMUSG00000084822	Myadml2os	0	4.745756	0	31.88593	14.58084	17.43201	3.409887	0.01164
532	ENSMUSG00000030643	Rab30	1345.321	637.9652	1222.317	342.1905	592.7112	523.6576	-1.13429	0.011684
533	ENSMUSG00000018340	Anxa6	753.513	1098.303	499.6633	1202.333	1866.348	2059.766	1.119689	0.011731
534	ENSMUSG00000038526	Car14	90.85499	123.3896	90.84788	237.2002	309.8429	154.099	1.190172	0.011758
535	ENSMUSG00000015083	C8g	743.5106	1930.845	991.0677	5552.041	2879.716	1429.425	1.426544	0.011771
536	ENSMUSG00000030972	Acsm5	238.3902	1440.676	512.0517	1489.306	1710.333	3363.681	1.580175	0.011792
537	ENSMUSG00000082658	Fau-ps2	1.667064	0	61.94173	1.555411	0.729042	0	-4.74387	0.011793
538	ENSMUSG00000030827	Fgf21	132.5316	258.3047	45.42394	48.99546	51.76199	47.41507	-1.58431	0.011796
539	ENSMUSG00000028646	Rragc	979.4002	630.5076	937.3849	339.8574	429.4058	503.4364	-0.99902	0.011801
540	ENSMUSG00000037415	Ranbp10	608.4784	884.7445	392.2976	1425.534	1119.08	1448.949	1.075359	0.011851
541	ENSMUSG00000041782	Lad1	20.8383	12.88134	20.64724	29.55282	99.87877	55.78243	1.798275	0.011879
542	ENSMUSG00000000154	Slc22a18	368.4212	1137.625	677.2296	1578.743	1822.605	1586.313	1.190591	0.011957
543	ENSMUSG00000030826	Bcat2	156.704	234.5759	148.6602	601.1665	263.1842	379.3205	1.194037	0.012022
544	ENSMUSG00000020075	Ddx21	971.8984	398.6435	1048.88	253.532	392.2247	396.0553	-1.21164	0.012075
545	ENSMUSG00000056035	Cyp3a11	6073.948	30724.02	11740.02	34507.58	47172.67	47475.73	1.411794	0.012092
546	ENSMUSG00000057685	Gm8526	0	0	41.29449	0	0	0	-6.61174	0.012221
547	ENSMUSG00000106708	Gm43782	0	0	41.29449	0	0	0	-6.61174	0.012221
548	ENSMUSG00000113054	Gm47514	0	0	41.29449	0	0	0	-6.61174	0.012221
549	ENSMUSG00000114132	Gm4808	0	0	41.29449	0	0	0	-6.61174	0.012221
550	ENSMUSG00000116951	AC163720.3	0	0	41.29449	0	0	0	-6.61174	0.012221
551	ENSMUSG00000000134	Tfe3	630.1502	164.0676	528.5695	179.65	159.6602	180.5956	-1.34332	0.012236
552	ENSMUSG00000032388	Spg21	674.3274	344.4063	747.4303	266.753	282.1393	278.9122	-1.08755	0.012237
553	ENSMUSG00000037411	Serpine1	14937.73	26.44064	8729.655	671.16	11.66467	34.16674	-5.04643	0.012242
554	ENSMUSG00000038418	Egr1	1755.419	138.9828	1205.799	535.8392	108.6273	140.1534	-1.98185	0.012248
555	ENSMUSG00000039043	Arpin	229.2213	67.79651	251.8964	56.77251	59.05241	81.5818	-1.4607	0.012282
556	ENSMUSG00000050737	Ptges	141.7004	10.16948	12.38835	17.10952	3.645211	5.578243	-2.67003	0.012322
557	ENSMUSG00000027111	Itga6	103.358	115.932	136.2718	63.77186	26.97456	49.50691	-1.33339	0.012328
558	ENSMUSG00000085860	2410003L11Rik	5.834724	1.35593	41.29449	0	0	2.091841	-4.41448	0.01242

Table S1. Differentially expressed genes from RNA-seq analysis of db/db mice livers (TAC vs. Veh; p-value < 0.05)										
	Gene_ID	Gene_Name	Veh_1	Veh_2	Veh_3	TAC_1	TAC_2	TAC_3	log2FoldChange	p-value
559	ENSMUSG00000025732	Mcrip2	161.7052	469.8298	293.1909	1661.179	477.5226	498.5555	1.510402	0.012457
560	ENSMUSG00000056116	H2-T22	335.0799	388.474	173.4369	869.4749	583.2337	541.7869	1.13821	0.012468
561	ENSMUSG00000040564	Apoc1	2349.727	6701.685	8799.856	31847.05	11648.63	8096.82	1.531595	0.012481
562	ENSMUSG00000014542	Clec4f	281.7338	624.4059	602.8995	1249.773	1202.19	829.7637	1.124717	0.012481
563	ENSMUSG00000021508	Cxcl14	178.3759	21.01692	78.45953	41.2184	11.66467	13.24833	-2.07712	0.012498
564	ENSMUSG00000040152	Thbs1	52.51252	41.35587	16.5178	5.44394	7.290421	14.64289	-2.05278	0.012591
565	ENSMUSG00000107868	Gm5112	0.833532	0.677965	53.68284	1.555411	0	0.69728	-4.53462	0.012678
566	ENSMUSG00000087700	Gm15283	25.83949	8.813546	28.90614	0	3.645211	4.183682	-2.97953	0.012679
567	ENSMUSG00000029597	Sds	1407.836	702.3719	1511.378	4378.483	2341.683	1755.055	1.228617	0.012695
568	ENSMUSG00000021360	Gcnt2	897.714	343.0503	582.2523	296.3059	291.6168	229.4052	-1.15778	0.012816
569	ENSMUSG00000079173	Zan	0.833532	0	41.29449	0.777706	0	0	-5.65378	0.01285
570	ENSMUSG00000111765	Gm10635	0.833532	0	41.29449	0.777706	0	0	-5.65378	0.01285
571	ENSMUSG00000054545	Ugt1a6a	73.35082	147.7964	107.3657	474.4005	148.7246	239.1672	1.38967	0.012871
572	ENSMUSG00000078139	AK157302	4.16766	6.101686	107.3657	1.555411	9.477547	1.394561	-3.17432	0.012876
573	ENSMUSG00000049047	Armcx3	42.51013	50.16942	37.16504	3.888528	12.39372	18.12929	-1.92314	0.01293
574	ENSMUSG00000071112	Spx	1.667064	0	61.94173	1.555411	0.729042	0.69728	-4.33902	0.012966
575	ENSMUSG00000106069	Gm6135	0	12.20337	0	59.88334	32.80689	22.31297	3.004623	0.012968
576	ENSMUSG00000025555	Farp1	514.2893	162.7116	309.7087	139.2093	124.6662	146.4289	-1.26674	0.012972
577	ENSMUSG00000028886	Eya3	621.8149	306.4402	569.864	159.4297	286.5135	230.1025	-1.14448	0.013079
578	ENSMUSG00000006235	Epor	2.500596	8.813546	0	24.10888	16.76797	43.23138	2.625714	0.013194
579	ENSMUSG00000068463	B630019A10Rik	59.18078	536.2704	330.3559	779.2611	895.9928	1132.383	1.601174	0.013219
580	ENSMUSG00000011148	Adssl1	152.5364	212.881	165.178	309.5269	324.4237	450.4431	1.026272	0.013227
581	ENSMUSG00000040170	Fmo2	130.031	755.9311	309.7087	1444.199	968.1679	921.1074	1.475122	0.013257
582	ENSMUSG00000057074	Ces1g	416.766	3142.368	1445.307	2344.783	5152.141	8305.307	1.658348	0.013262
583	ENSMUSG00000030650	Tmc5	2.500596	0	0	32.66364	15.30988	2.789122	3.958083	0.013272
584	ENSMUSG00000048148	Nwd1	0	1.35593	53.68284	0	0	1.394561	-5.20091	0.013289
585	ENSMUSG00000033287	Kctd17	185.0441	91.52529	103.2362	69.99351	40.82636	38.35042	-1.35875	0.013444
586	ENSMUSG00000030378	Sult2a8	80.01908	2759.996	1300.776	3977.187	8615.82	4114.652	2.012283	0.013457
587	ENSMUSG00000024922	Ovol1	62.5149	4.067791	33.03559	9.332468	1.458084	0	-3.21639	0.013463
588	ENSMUSG00000059908	Mug1	17312.46	22285.39	23380.94	29780.68	58360.55	37525.54	0.996739	0.013487
589	ENSMUSG00000001604	Tcea3	77.51848	493.5586	161.0485	1066.234	521.9942	615.6986	1.581574	0.013514

Table S1. Differentially expressed genes from RNA-seq analysis of db/db mice livers (TAC vs. Veh; p-value < 0.05)

	Gene_ID	Gene_Name	Veh_1	Veh_2	Veh_3	TAC_1	TAC_2	TAC_3	log2FoldChange	p-value
590	ENSMUSG00000073926	Olfr653	0	1.35593	28.90614	0	0	0	-6.13383	0.01355
591	ENSMUSG00000097434	Gm16630	0	1.35593	28.90614	0	0	0	-6.13383	0.01355
592	ENSMUSG00000057322	Rpl38	109.1927	117.9659	8.258898	603.4996	212.1513	113.6567	1.94291	0.013666
593	ENSMUSG00000064254	Ethe1	180.0429	204.0675	231.2491	549.0602	352.8564	341.6674	1.022223	0.013724
594	ENSMUSG00000021456	Fbp2	18.33771	8.135581	66.07118	8.554762	2.187126	0	-3.05856	0.013739
595	ENSMUSG00000021947	Cryl1	89.18793	145.0845	123.8835	217.7576	209.2351	369.5586	1.155371	0.013784
596	ENSMUSG00000025225	Nfkb2	488.4498	101.6948	111.4951	79.32598	86.02697	69.03076	-1.5928	0.013785
597	ENSMUSG00000025036	Sfxn2	305.0727	457.6264	359.2621	900.5832	725.3969	588.5046	0.979049	0.013852
598	ENSMUSG00000032128	Robo3	1.667064	4.067791	0	31.88593	11.66467	13.94561	3.004996	0.013873
599	ENSMUSG00000050373	Snx21	97.52325	67.79651	66.07118	251.9766	141.4342	147.1262	1.209938	0.013921
600	ENSMUSG00000072487	Mroh5	0	0	49.55339	0.777706	0	0	-5.90976	0.014145
601	ENSMUSG00000112404	AC159282.1	0	0	49.55339	0.777706	0	0	-5.90976	0.014145
602	ENSMUSG00000021687	Scamp1	1611.217	700.338	1581.579	478.289	639.3699	688.913	-1.1056	0.014151
603	ENSMUSG00000020804	Aanat	0	0	49.55339	0	0	0.69728	-5.90981	0.014174
604	ENSMUSG00000021048	Mthfd1	443.4391	1734.235	747.4303	1540.635	2700.372	3241.656	1.353106	0.01418
605	ENSMUSG00000057561	Eif1a	3168.255	564.7449	2304.233	973.6875	647.3894	631.736	-1.42163	0.014201
606	ENSMUSG00000045273	Cenph	0.833532	7.457616	53.68284	0.777706	1.458084	2.789122	-3.51555	0.014282
607	ENSMUSG00000031167	Rbm3	1574.542	481.3552	1560.932	708.4899	300.3653	413.4873	-1.34463	0.014329
608	ENSMUSG00000045136	Tubb2b	101.6909	86.77953	37.16504	35.77446	0.729042	4.183682	-2.49037	0.014343
609	ENSMUSG00000044349	Snhg11	200.8812	427.118	227.1197	906.8048	342.6498	824.1854	1.271679	0.014351
610	ENSMUSG00000018604	Tbx3	109.1927	159.3218	45.42394	336.7466	164.0345	326.3272	1.361739	0.014357
611	ENSMUSG00000040562	Gstm2	150.0358	654.9143	210.6019	727.1548	1064.401	896.7026	1.396247	0.014375
612	ENSMUSG00000021067	Sav1	196.7136	141.6947	309.7087	44.32922	97.69164	113.6567	-1.32554	0.014392
613	ENSMUSG00000022364	Tbc1d31	115.861	660.338	128.0129	28.77511	104.9821	122.0241	-1.82924	0.014418
614	ENSMUSG00000015536	Mocs2	717.6711	1322.71	929.126	2358.781	1813.128	1705.548	0.983892	0.014479
615	ENSMUSG00000040147	Maob	596.8089	2294.234	978.6794	2193.13	4414.35	3139.156	1.331098	0.014485
616	ENSMUSG00000076613	Ighg2b	11.66945	2.71186	0	5.44394	46.6587	61.36067	2.788555	0.014503
617	ENSMUSG00000020072	Pbld2	174.2082	1488.811	532.6989	2546.208	2042.047	1903.575	1.561586	0.014584
618	ENSMUSG00000105315	Gm18635	0	2.033895	0	22.55346	10.20659	8.367365	3.803576	0.014644
619	ENSMUSG00000032607	Amt	165.0393	447.457	70.20063	418.4056	691.861	873.6923	1.521652	0.01471
620	ENSMUSG00000081578	Gm12611	0	5.423721	45.42394	0	1.458084	0.69728	-4.46352	0.014721

Table S1. Differentially expressed genes from RNA-seq analysis of db/db mice livers (TAC vs. Veh; p-value < 0.05)										
	Gene_ID	Gene_Name	Veh_1	Veh_2	Veh_3	TAC_1	TAC_2	TAC_3	log2FoldChange	p-value
621	ENSMUSG00000109482	Gm4756	60.84784	473.2196	49.55339	419.1834	713.7322	978.2844	1.839459	0.014732
622	ENSMUSG00000061843	Vmn1r-ps32	0	0	0	16.33182	7.290421	2.091841	5.132968	0.014783
623	ENSMUSG00000031173	Otc	127.5304	2602.03	867.1843	3168.373	5375.227	4249.227	1.829501	0.014789
624	ENSMUSG00000087026	A230103J11Rik	2.500596	2.71186	66.07118	1.555411	2.187126	2.789122	-3.33864	0.014816
625	ENSMUSG00000112654	4930455C13Rik	4.16766	5.423721	61.94173	0	2.916168	3.486402	-3.38079	0.014849
626	ENSMUSG00000020623	Map2k6	2.500596	48.13552	20.64724	112.7673	91.85931	88.55461	2.018419	0.014873
627	ENSMUSG00000049858	Suox	586.8066	1389.828	495.5339	1097.343	2575.706	2393.066	1.291003	0.01498
628	ENSMUSG00000044986	Tst	1179.448	1431.862	1449.437	3259.364	2368.658	2011.654	0.912504	0.015025
629	ENSMUSG00000094152	Slc6a16	0	20.33895	16.5178	66.88269	35.72306	91.34373	2.440369	0.015067
630	ENSMUSG00000027947	Il6ra	453.4414	820.3378	359.2621	851.5877	1697.21	1105.189	1.155602	0.015068
631	ENSMUSG00000039648	Kyat1	631.8173	452.2027	594.6406	1722.618	1146.783	702.1613	1.090524	0.015098
632	ENSMUSG00000027985	Lef1	0	2.71186	33.03559	0	0.729042	0	-5.4056	0.015147
633	ENSMUSG00000022323	Rida	475.9468	4637.281	1643.521	7202.332	7237.201	5792.308	1.581491	0.015184
634	ENSMUSG00000000340	Dbt	241.7243	704.4057	272.5436	402.8515	1602.435	1385.496	1.471119	0.015205
635	ENSMUSG00000107102	Gm42726	5.001192	1.35593	57.81228	3.110823	1.458084	0	-3.73505	0.015215
636	ENSMUSG00000032802	Srxn1	1552.037	582.372	1486.602	441.7368	369.6243	739.8145	-1.22108	0.015221
637	ENSMUSG00000032508	Myd88	986.0684	159.3218	887.8315	318.8593	191.7381	205.0004	-1.50377	0.015229
638	ENSMUSG00000073633	Fbxo36	73.35082	86.77953	41.29449	309.5269	125.3952	112.9594	1.409617	0.01524
639	ENSMUSG00000031270	4930513O06Rik	5.001192	2.71186	57.81228	1.555411	2.916168	2.091841	-3.20165	0.015252
640	ENSMUSG00000023961	Enpp4	711.8364	223.0505	557.4756	122.0998	309.8429	144.337	-1.37063	0.015362
641	ENSMUSG00000056131	Pgm3	600.1431	301.6945	247.7669	138.4316	228.9192	130.3914	-1.21525	0.015516
642	ENSMUSG00000109505	Gm44677	3.334128	0	41.29449	0.777706	0.729042	0	-4.80975	0.015533
643	ENSMUSG00000115980	Gm49437	3.334128	7.457616	4.129449	57.55022	17.49701	11.85377	2.486909	0.015554
644	ENSMUSG00000003585	Sec14l2	1547.869	5270.501	2444.634	6526.506	6305.485	8531.225	1.204891	0.015555
645	ENSMUSG00000022304	Dpys	437.6043	2675.928	1073.657	3060.272	3730.508	4471.659	1.426239	0.01556
646	ENSMUSG00000026853	Crat	2425.578	2781.013	2019.301	1743.616	949.9419	868.1141	-1.02158	0.01558
647	ENSMUSG00000032816	Igdcc4	1261.134	8.135581	896.0904	102.6571	7.290421	6.275523	-4.21992	0.015612
648	ENSMUSG00000045775	Slc16a5	166.7064	593.8974	280.8025	691.3803	723.9388	1124.016	1.281034	0.015615
649	ENSMUSG00000115882	Gm5481	2.500596	1.35593	0	31.88593	9.477547	6.972804	3.315722	0.015615
650	ENSMUSG00000110607	Gm45843	0	2.71186	24.77669	0	0	0	-5.99515	0.015671
651	ENSMUSG00000024254	Abcg8	667.6592	949.1512	512.0517	1322.1	1278.011	1543.081	0.955001	0.015696

Table S1. Differentially expressed genes from RNA-seq analysis of db/db mice livers (TAC vs. Veh; p-value < 0.05)										
	Gene_ID	Gene_Name	Veh_1	Veh_2	Veh_3	TAC_1	TAC_2	TAC_3	log2FoldChange	p-value
652	ENSMUSG00000039236	Isg20	44.1772	25.08471	218.8608	35.77446	14.58084	20.22113	-1.98453	0.0157
653	ENSMUSG00000007035	Msh5	5.001192	24.40674	4.129449	60.66104	39.36827	48.80963	2.020117	0.015768
654	ENSMUSG00000022351	Sqle	4922.007	593.2195	3068.181	78.54827	511.7876	1499.153	-2.03817	0.015851
655	ENSMUSG00000040297	Suco	699.3334	300.3385	640.0646	289.3065	252.9776	221.7352	-1.09822	0.015885
656	ENSMUSG00000025789	St8sia2	0	4.745756	45.42394	0	1.458084	0.69728	-4.44249	0.015887
657	ENSMUSG00000028456	Unc13b	85.8538	62.37279	99.10677	9.332468	29.16168	41.83682	-1.60711	0.015904
658	ENSMUSG00000040505	Abcg5	632.6508	776.948	883.7021	2391.445	1065.131	1315.768	1.059482	0.015933
659	ENSMUSG00000029406	Pitpnm2	334.2464	405.4231	289.0614	643.9403	577.4014	709.8314	0.901468	0.015936
660	ENSMUSG00000031380	Vegfd	16.67064	18.30506	136.2718	10.88788	3.645211	18.12929	-2.32701	0.01604
661	ENSMUSG00000062822	4833420G17Rik	587.6401	395.9316	838.2781	424.6273	174.2411	139.4561	-1.29861	0.016063
662	ENSMUSG00000004610	Etfb	331.7458	255.5928	371.6504	1609.851	499.3938	380.7151	1.379912	0.016083
663	ENSMUSG00000061897	Gm14292	41.6766	47.45756	53.68284	6.221645	16.76797	20.22113	-1.70991	0.01609
664	ENSMUSG00000079105	C7	11.66945	4.067791	61.94173	3.888528	1.458084	4.880963	-2.82422	0.016189
665	ENSMUSG00000010362	Rdm1	33.34128	44.7457	28.90614	96.4355	91.85931	77.39812	1.278139	0.016239
666	ENSMUSG00000035472	Slc25a21	70.85022	101.0168	49.55339	211.5359	121.75	191.7521	1.211026	0.016254
667	ENSMUSG00000029455	Aldh2	5212.076	15698.96	7928.542	17693.58	20238.21	26059.46	1.149623	0.016264
668	ENSMUSG00000075588	Hoxb2	0	7.457616	24.77669	0	0	0.69728	-5.27404	0.016299
669	ENSMUSG00000068130	Zfp442	5.001192	11.52541	103.2362	2.333117	9.477547	5.578243	-2.70474	0.016334
670	ENSMUSG00000034165	Ccnd3	155.8705	299.6606	90.84788	482.1775	443.2576	362.5858	1.213852	0.016393
671	ENSMUSG00000026499	Acbd3	637.652	275.9318	660.7118	186.6494	271.2037	257.9937	-1.13156	0.016472
672	ENSMUSG00000021071	Trim9	0	0	37.16504	0	0	0	-6.45631	0.016525
673	ENSMUSG00000031620	Iqcm	0	0	37.16504	0	0	0	-6.45631	0.016525
674	ENSMUSG00000031952	Chst5	0	0	37.16504	0	0	0	-6.45631	0.016525
675	ENSMUSG00000038094	Atp13a4	0	0	37.16504	0	0	0	-6.45631	0.016525
676	ENSMUSG00000038665	Dgki	0	0	37.16504	0	0	0	-6.45631	0.016525
677	ENSMUSG00000046934	Csl	0	0	37.16504	0	0	0	-6.45631	0.016525
678	ENSMUSG00000050641	BC048562	0	0	37.16504	0	0	0	-6.45631	0.016525
679	ENSMUSG00000075062	Olfr1271	0	0	37.16504	0	0	0	-6.45631	0.016525
680	ENSMUSG00000081490	Gm11830	0	0	37.16504	0	0	0	-6.45631	0.016525
681	ENSMUSG00000082152	Gm13655	0	0	37.16504	0	0	0	-6.45631	0.016525
682	ENSMUSG00000083016	Gm12618	0	0	37.16504	0	0	0	-6.45631	0.016525

Table S1. Differentially expressed genes from RNA-seq analysis of db/db mice livers (TAC vs. Veh; p-value < 0.05)

	Gene_ID	Gene_Name	Veh_1	Veh_2	Veh_3	TAC_1	TAC_2	TAC_3	log2FoldChange	p-value
683	ENSMUSG00000090461	Iltifb	0	0	37.16504	0	0	0	-6.45631	0.016525
684	ENSMUSG00000093859	Gm7882	0	0	37.16504	0	0	0	-6.45631	0.016525
685	ENSMUSG00000101414	Gm29101	0	0	37.16504	0	0	0	-6.45631	0.016525
686	ENSMUSG00000101968	1700027A15Rik	0	0	37.16504	0	0	0	-6.45631	0.016525
687	ENSMUSG00000103948	4930594C11Rik	0	0	37.16504	0	0	0	-6.45631	0.016525
688	ENSMUSG00000105442	Gm42614	0	0	37.16504	0	0	0	-6.45631	0.016525
689	ENSMUSG00000111144	Gm48193	0	0	37.16504	0	0	0	-6.45631	0.016525
690	ENSMUSG00000113542	Gm19154	0	0	37.16504	0	0	0	-6.45631	0.016525
691	ENSMUSG00000021025	Nfkbia	1635.39	311.186	1676.556	470.5119	483.3549	423.9465	-1.39259	0.016533
692	ENSMUSG00000044469	Tnfaip8l1	61.68137	250.8471	107.3657	419.9611	389.3085	275.4258	1.356615	0.016584
693	ENSMUSG00000026623	Lpgat1	12336.27	3819.655	8560.348	3391.574	4435.492	3323.238	-1.1483	0.016597
694	ENSMUSG00000024843	Chka	1149.441	561.3551	772.2069	559.9481	293.804	276.8203	-1.13589	0.016602
695	ENSMUSG00000021938	Pspc1	221.7195	90.16936	491.4044	92.54697	64.88475	124.1159	-1.49395	0.016622
696	ENSMUSG00000036570	Fxyd1	701.834	1130.846	1003.456	3204.925	1508.388	1397.35	1.107902	0.016684
697	ENSMUSG00000090264	Eif4ebp3	8.335321	2.71186	0	41.99611	27.7036	10.45921	2.624289	0.016686
698	ENSMUSG00000024268	Celf4	8.335321	7.457616	82.58898	4.666234	7.290421	2.789122	-2.6444	0.016705
699	ENSMUSG00000032184	Lysmd2	3.334128	6.779651	53.68284	0	3.645211	2.091841	-3.37197	0.016737
700	ENSMUSG00000108083	Mug4-ps	20.00477	19.66099	0	55.2171	70.71708	39.74498	1.888961	0.016744
701	ENSMUSG00000041920	Slc16a6	164.2058	134.9151	235.3786	73.88204	77.27846	95.52741	-1.0961	0.016816
702	ENSMUSG00000078887	Gm6710	3.334128	10.84744	37.16504	2.333117	1.458084	2.091841	-3.01403	0.016844
703	ENSMUSG00000025816	Sec61a2	134.1987	100.3388	181.6958	37.32987	53.94912	80.18724	-1.26025	0.016862
704	ENSMUSG00000047230	Cldn2	4130.151	1134.914	2333.139	299.4167	1106.686	1333.2	-1.47188	0.016905
705	ENSMUSG00000030341	Tnfrsf1a	2470.589	726.7786	1845.864	724.8217	658.325	847.8929	-1.17594	0.016926
706	ENSMUSG00000047216	Cdh19	0	0	0	10.11017	5.832337	6.972804	4.967894	0.016943
707	ENSMUSG00000010751	Tnfrsf22	41.6766	34.57622	20.64724	3.110823	9.477547	11.85377	-2.0145	0.01705
708	ENSMUSG00000022820	Ndufb4	210.8836	402.0333	289.0614	1056.902	587.6079	404.4226	1.181957	0.017057
709	ENSMUSG00000094806	Cyp2d10	3812.576	7417.616	3638.045	12185.09	8350.448	9694.289	1.023041	0.017083
710	ENSMUSG00000038702	Dsel	4.16766	8.135581	136.2718	6.221645	7.290421	8.367365	-2.69554	0.017093
711	ENSMUSG00000050541	Adra1b	256.7279	743.0498	363.3915	957.3557	825.2757	1303.914	1.174576	0.017099
712	ENSMUSG00000028964	Park7	1022.744	1439.32	821.7603	4009.073	1643.99	1678.354	1.156439	0.017104
713	ENSMUSG00000048038	Ccdc187	0	0.677965	37.16504	0.777706	0	0	-5.4927	0.01719

Table S1. Differentially expressed genes from RNA-seq analysis of db/db mice livers (TAC vs. Veh; p-value < 0.05)

	Gene_ID	Gene_Name	Veh_1	Veh_2	Veh_3	TAC_1	TAC_2	TAC_3	log2FoldChange	p-value
714	ENSMUSG00000021250	Fos	16.67064	10.84744	0	122.8775	16.76797	23.01025	2.406413	0.017245
715	ENSMUSG00000047228	A2ml1	326.7446	6.779651	553.3462	1.555411	45.92965	3.486402	-4.11839	0.017346
716	ENSMUSG00000048832	Vps37c	344.2487	105.0846	289.0614	71.54892	100.6078	124.1159	-1.31111	0.017389
717	ENSMUSG00000037606	Osbpl5	41.6766	115.2541	28.90614	142.3201	178.6153	189.6603	1.40866	0.017403
718	ENSMUSG00000089678	Agxt2	802.6914	2414.912	1123.21	4249.384	2768.173	2781.451	1.173074	0.017464
719	ENSMUSG00000082394	Gm4596	7.501788	0.677965	94.97733	1.555411	3.645211	5.578243	-3.18263	0.017511
720	ENSMUSG00000020901	Pik3r5	30.84069	25.08471	61.94173	3.888528	17.49701	6.972804	-2.00601	0.017575
721	ENSMUSG00000055833	1700034H15Rik	6.668256	13.5593	49.55339	5.44394	3.645211	0.69728	-2.75199	0.017641
722	ENSMUSG00000025911	Adhfe1	451.7744	769.4904	445.9805	909.9156	1192.713	1165.156	0.965888	0.017669
723	ENSMUSG00000094174	Ighv6-4	0	0	45.42394	0	0.729042	0	-5.78063	0.017797
724	ENSMUSG00000112797	Gm30034	0	0	45.42394	0	0.729042	0	-5.78063	0.017797
725	ENSMUSG00000064115	Cadm2	0	0	45.42394	0	0	0.69728	-5.78066	0.017812
726	ENSMUSG00000103865	Gm37416	63.34844	54.23721	181.6958	34.99676	43.74253	19.52385	-1.56439	0.017826
727	ENSMUSG00000085316	D330050G23Rik	0	2.033895	24.77669	0	0	0	-5.95603	0.017889
728	ENSMUSG00000018459	Slc13a3	393.4271	108.4744	235.3786	839.9221	546.0525	417.6709	1.290112	0.017902
729	ENSMUSG00000110781	Gm31992	3.334128	13.5593	0	24.88658	38.63923	35.5613	2.315392	0.017928
730	ENSMUSG00000055013	Agap1	405.9301	102.3727	487.275	125.9883	142.1632	113.6567	-1.37288	0.017932
731	ENSMUSG00000074336	Apoc4	1995.476	2660.335	2799.766	13728.06	3710.824	2502.539	1.41966	0.017951
732	ENSMUSG00000021794	Glud1	4251.847	9343.715	5339.377	13853.27	12989.34	10934.75	0.996123	0.017953
733	ENSMUSG00000089698	Gm2541	5.834724	8.135581	132.1424	0.777706	6.561379	11.85377	-2.86567	0.017954
734	ENSMUSG00000083813	Gm15502	55.84665	35.93215	49.55339	367.8548	73.63325	46.02051	1.790518	0.017973
735	ENSMUSG00000022763	Aifm3	13.33651	52.20331	0	101.8794	56.13624	127.6023	2.009509	0.01804
736	ENSMUSG00000041132	N4bp2l1	143.3675	717.2871	260.1553	1581.853	892.3475	614.304	1.457033	0.018073
737	ENSMUSG00000024386	Proc	2993.214	3442.707	3262.265	9941.412	4852.504	4706.643	1.007733	0.018091
738	ENSMUSG00000022235	Cmbl	863.5392	1633.896	1441.178	2274.011	2593.932	2645.482	0.932707	0.018155
739	ENSMUSG00000024842	Cabp4	0	1.35593	0	8.554762	16.03893	8.367365	3.927884	0.01825
740	ENSMUSG00000031168	Ebp	1025.244	1540.337	1007.586	2393	2596.848	1769	0.917535	0.018272
741	ENSMUSG00000026175	Vil1	0	0	0	38.10758	0	0.69728	5.726925	0.018306
742	ENSMUSG00000112850	Gm9176	0.833532	0.677965	49.55339	0.777706	1.458084	0.69728	-4.01068	0.018308
743	ENSMUSG00000021226	Acot2	550.9647	1715.252	384.0388	222.4238	360.1468	433.0111	-1.38802	0.018353
744	ENSMUSG00000048217	Nags	493.451	408.813	206.4724	913.0265	704.2547	695.1885	1.047666	0.018411

Table S1. Differentially expressed genes from RNA-seq analysis of db/db mice livers (TAC vs. Veh; p-value < 0.05)

	Gene_ID	Gene_Name	Veh_1	Veh_2	Veh_3	TAC_1	TAC_2	TAC_3	log2FoldChange	p-value
745	ENSMUSG00000020309	Chac2	77.51848	174.915	74.33008	342.9682	282.8683	168.0446	1.258173	0.01843
746	ENSMUSG00000029167	Ppargc1a	113.3604	203.3895	86.71843	223.9792	458.5675	266.3611	1.212443	0.018435
747	ENSMUSG00000034177	Rnf43	68.34963	151.1862	49.55339	142.3201	185.9057	409.3036	1.425923	0.018451
748	ENSMUSG00000028163	Nfkb1	781.0195	297.6267	656.5824	248.0881	255.8938	304.0142	-1.09988	0.018636
749	ENSMUSG00000039470	Zdhhc2	3.334128	4.067791	49.55339	2.333117	1.458084	2.091841	-3.14828	0.018662
750	ENSMUSG00000026675	Hsd17b7	4265.184	1423.049	2989.721	347.6344	784.4493	1980.276	-1.47913	0.018663
751	ENSMUSG00000041313	Slc7a1	85.8538	25.76267	0	2.333117	0.729042	11.15649	-3.00214	0.018684
752	ENSMUSG00000052605	Isoc2b	31.67422	161.3557	49.55339	275.3078	191.7381	223.827	1.482887	0.018694
753	ENSMUSG00000004099	Dnmt1	244.2249	166.1015	181.6958	66.10498	87.48505	122.7213	-1.10198	0.018755
754	ENSMUSG00000052117	D630039A03Rik	53.34605	53.55924	16.5178	215.4245	49.57486	125.5105	1.603301	0.0188
755	ENSMUSG00000030528	Blm	20.8383	73.22023	4.129449	204.5366	59.05241	120.6295	1.888159	0.018827
756	ENSMUSG00000027660	Skil	760.1812	247.4573	648.3235	227.8678	271.2037	239.8645	-1.16087	0.018875
757	ENSMUSG00000006782	Cnp	50.01192	78.64395	37.16504	118.2113	135.6018	135.9697	1.189431	0.018932
758	ENSMUSG00000039063	Echdc3	119.1951	707.1176	309.7087	803.37	1031.595	1140.053	1.385286	0.01895
759	ENSMUSG00000059027	9630013D21Rik	40.84307	84.74564	24.77669	265.1976	90.40122	96.92197	1.542317	0.018971
760	ENSMUSG00000031443	F7	565.9683	472.5417	813.5014	1171.225	1123.454	1137.264	0.897759	0.019048
761	ENSMUSG00000109644	0610005C13Rik	876.0422	1245.422	896.0904	3258.587	1498.911	1528.439	1.057416	0.019095
762	ENSMUSG00000054619	Mettl7a1	477.6139	2195.929	1065.398	2329.228	3387.859	3348.34	1.276687	0.019121
763	ENSMUSG00000029630	Cyp3a25	2598.119	12195.91	5818.394	27422.68	11291.4	14363.28	1.364401	0.019135
764	ENSMUSG00000039217	Il18	42.51013	70.50837	28.90614	155.5411	110.0854	94.83013	1.293514	0.019146
765	ENSMUSG00000052040	Klf13	355.9182	512.5416	375.7799	727.1548	804.8625	750.2737	0.871374	0.019263
766	ENSMUSG00000063558	Aox1	521.7911	1267.117	495.5339	1934.154	1116.893	2114.154	1.172683	0.019313
767	ENSMUSG00000050552	Lamtor4	172.5411	229.1522	152.7896	920.8035	314.9462	205.6977	1.371943	0.019365
768	ENSMUSG00000053134	Supt7l	140.0334	145.0845	334.4854	94.88009	90.40122	82.27908	-1.18555	0.019367
769	ENSMUSG00000025035	Arl3	67.5161	76.61006	90.84788	189.7602	169.8668	126.905	1.068468	0.019405
770	ENSMUSG00000020051	Pah	591.8078	9011.512	2560.258	8847.18	21391.55	10374.14	1.738955	0.019406
771	ENSMUSG00000027737	Slc7a11	21.67183	4.745756	4.129449	1.555411	0	0	-4.37639	0.019415
772	ENSMUSG00000030786	Itgam	29.17362	6.779651	66.07118	10.88788	1.458084	5.578243	-2.45187	0.019579
773	ENSMUSG00000031422	Morf4l2	2044.654	850.1682	1796.31	520.2851	954.3161	725.8689	-1.09097	0.019601
774	ENSMUSG00000040146	Rgl3	217.5519	212.2031	53.68284	509.3972	307.6558	383.5042	1.28391	0.019622
775	ENSMUSG00000090946	Ccdc71l	232.5554	84.74564	256.0258	84.76992	89.67218	59.96611	-1.27676	0.019698

Table S1. Differentially expressed genes from RNA-seq analysis of db/db mice livers (TAC vs. Veh; p-value < 0.05)										
	Gene_ID	Gene_Name	Veh_1	Veh_2	Veh_3	TAC_1	TAC_2	TAC_3	log2FoldChange	p-value
776	ENSMUSG00000026749	Nek6	1602.882	530.1687	1114.951	586.3901	452.0061	461.5996	-1.11418	0.019767
777	ENSMUSG00000106930	Gm6450	9.168853	0	111.4951	0.777706	0	0	-7.24491	0.019796
778	ENSMUSG00000040026	Saa3	2231.365	21.01692	2440.504	275.3078	29.16168	23.01025	-3.84088	0.019811
779	ENSMUSG00000039519	Cyp7b1	10.00238	115.932	78.45953	173.4284	291.6168	189.6603	1.686065	0.019861
780	ENSMUSG00000033411	Ctdspl2	149.2022	129.4913	317.9676	32.66364	102.7949	89.94917	-1.38435	0.019974
781	ENSMUSG00000034487	Kdelc2	93.35559	148.4744	140.4013	61.43875	55.4072	59.96611	-1.10562	0.019975
782	ENSMUSG00000061825	Ces2c	85.02027	130.8473	41.29449	24.88658	492.8325	462.2969	1.910965	0.02007
783	ENSMUSG00000037573	Tob1	623.482	1050.168	491.4044	906.8048	1990.285	1780.854	1.10703	0.020083
784	ENSMUSG00000092270	Gm7031	1.667064	0	24.77669	0	0	0	-5.93582	0.020135
785	ENSMUSG00000025155	Dus1l	556.7994	474.5756	553.3462	1832.275	733.4164	800.4779	1.087745	0.020158
786	ENSMUSG00000042377	Fam83g	72.51729	18.98302	90.84788	3.888528	18.22605	23.70753	-1.96233	0.02017
787	ENSMUSG00000069014	Gm5641	105.8586	45.42366	132.1424	17.10952	43.01348	39.0477	-1.48982	0.020181
788	ENSMUSG00000103882	Gm37452	0	0	53.68284	0.777706	0.729042	0	-5.10055	0.020182
789	ENSMUSG00000021367	Edn1	0.833532	0.677965	0	3.888528	7.290421	20.91841	3.828518	0.020225
790	ENSMUSG00000100094	1810008I18Rik	249.2261	559.9992	293.1909	107.3234	179.3444	209.1841	-1.15763	0.020239
791	ENSMUSG00000074899	Sptbn5	8.335321	0.677965	28.90614	0.777706	0	1.394561	-4.01756	0.02026
792	ENSMUSG00000028034	Fubp1	528.4593	518.6433	561.6051	181.2054	356.5016	285.885	-0.96451	0.020277
793	ENSMUSG00000042688	Mapk6	1587.045	382.3723	1106.692	381.0758	392.2247	531.3276	-1.23657	0.020294
794	ENSMUSG00000051344	Plekhm3	91.68853	63.05076	140.4013	34.99676	43.74253	41.83682	-1.25799	0.020314
795	ENSMUSG00000068874	Selenbp1	820.1955	2589.827	1069.527	2913.285	3357.968	3651.657	1.14551	0.020345
796	ENSMUSG00000079067	Hmgn2-ps1	23.3389	11.52541	45.42394	5.44394	4.374253	7.670084	-2.12268	0.020386
797	ENSMUSG00000091345	Col6a5	2.500596	1.35593	86.71843	6.221645	1.458084	0.69728	-3.36924	0.020476
798	ENSMUSG00000074340	Ovgp1	2.500596	2.71186	0	25.66429	10.93563	11.85377	2.905269	0.020543
799	ENSMUSG00000025017	Pik3ap1	1847.941	553.2195	1032.362	61.43875	460.7546	585.7155	-1.63191	0.020568
800	ENSMUSG00000042198	Chchd7	62.5149	67.11855	82.58898	244.9773	134.1437	104.5921	1.205278	0.02059
801	ENSMUSG00000084230	Gm14388	12.50298	25.76267	173.4369	5.44394	19.68414	18.82657	-2.2129	0.020649
802	ENSMUSG00000028411	Aptx	77.51848	112.5422	70.20063	200.6481	147.9955	204.3032	1.06603	0.020654
803	ENSMUSG00000025105	Bnc1	1.667064	13.5593	70.20063	0.777706	0.729042	7.670084	-3.14002	0.020661
804	ENSMUSG00000035561	Aldh1b1	300.0715	593.8974	194.0841	447.9585	797.5721	1494.272	1.323531	0.02074
805	ENSMUSG00000056145	AI504432	0	1.35593	49.55339	0.777706	0	1.394561	-4.45802	0.020767
806	ENSMUSG00000019806	Aig1	786.8543	181.0167	623.5468	268.3085	223.8159	147.1262	-1.31343	0.020841

Table S1. Differentially expressed genes from RNA-seq analysis of db/db mice livers (TAC vs. Veh; p-value < 0.05)										
	Gene_ID	Gene_Name	Veh_1	Veh_2	Veh_3	TAC_1	TAC_2	TAC_3	log2FoldChange	p-value
807	ENSMUSG00000037852	Cpe	16.67064	28.47453	70.20063	3.110823	0	14.64289	-2.65616	0.020924
808	ENSMUSG00000071103	1700029J07Rik	11.66945	14.91523	4.129449	54.4394	32.07785	31.37762	1.806691	0.020958
809	ENSMUSG00000017718	Afmid	46.67779	403.3892	45.42394	430.0712	570.84	647.7735	1.716159	0.020998
810	ENSMUSG00000026839	Upp2	30.84069	2010.167	718.5241	4112.508	4479.964	2480.226	2.003643	0.021127
811	ENSMUSG00000030681	Mvp	2840.677	582.372	1816.958	904.4717	517.6199	718.8961	-1.29104	0.021297
812	ENSMUSG00000097057	Gm17638	0.833532	0	86.71843	3.888528	0.729042	0	-4.20605	0.021324
813	ENSMUSG00000007812	Zfp655	555.1323	647.4567	830.0192	245.755	339.7336	453.9295	-0.96313	0.021325
814	ENSMUSG00000041809	Efhc1	2.500596	1.35593	86.71843	3.110823	4.374253	2.091841	-3.15909	0.02135
815	ENSMUSG00000057342	Sphk2	340.0811	362.0334	210.6019	664.9383	513.9747	575.2563	0.929349	0.021437
816	ENSMUSG00000083863	Gm13341	2.500596	3.389826	61.94173	3.110823	1.458084	2.789122	-3.09424	0.02147
817	ENSMUSG00000060275	Nrg2	0.833532	0	49.55339	1.555411	0	0	-4.97493	0.021554
818	ENSMUSG00000025757	Hspa4l	400.0954	323.3894	714.3947	104.2126	326.6109	172.2283	-1.24552	0.021695
819	ENSMUSG00000031429	Psmd10	267.5638	145.7625	268.4142	109.6565	117.3758	103.1975	-1.03664	0.021799
820	ENSMUSG00000039967	Zfp292	573.4701	317.9656	619.4173	189.7602	268.2875	288.6741	-1.01099	0.021803
821	ENSMUSG00000049922	Slc35c1	350.0835	175.593	293.1909	119.7667	153.0988	126.2077	-1.03335	0.021942
822	ENSMUSG00000031766	Slc12a3	0.833532	9.491512	78.45953	3.110823	3.645211	4.183682	-2.93444	0.02197
823	ENSMUSG00000054136	Adm2	70.85022	0	119.754	1.555411	2.916168	0	-5.41005	0.021996
824	ENSMUSG00000009145	Dqx1	83.35321	153.8981	16.5178	360.0777	178.6153	209.1841	1.514441	0.022189
825	ENSMUSG00000002985	Apoe	58618.14	147616.7	86041.2	264596.5	170954.5	167317.3	1.044497	0.022195
826	ENSMUSG00000030142	Clec4e	18.33771	6.101686	0	0	0.729042	0	-5.01896	0.022198
827	ENSMUSG00000025825	Iscu	614.3131	752.5413	384.0388	1964.485	971.8131	834.6446	1.101116	0.022312
828	ENSMUSG00000058922	Gm10052	56.68018	51.52535	355.1326	34.99676	14.58084	73.21444	-1.88674	0.022332
829	ENSMUSG00000105095	8430422M14Rik	7.501788	33.22029	0	118.989	47.38774	32.0749	2.142969	0.022346
830	ENSMUSG00000074254	Cyp2a4	9.168853	2067.794	86.71843	7.777057	55.4072	67.6362	-4.04846	0.022441
831	ENSMUSG00000071414	Gm6736	5.001192	0.677965	41.29449	2.333117	0.729042	0	-3.85507	0.022472
832	ENSMUSG00000029269	Sult1b1	101.6909	467.7959	322.097	570.0583	834.0242	734.2362	1.263364	0.02254
833	ENSMUSG00000020256	Aldh1l2	30.00715	2.033895	0	0	0.729042	0.69728	-4.53043	0.022579
834	ENSMUSG00000067889	Sptbn2	638.4856	649.4906	268.4142	748.9306	1038.156	1631.636	1.126994	0.022602
835	ENSMUSG00000003617	Cp	60982.04	14140.32	46869.25	23338.95	16762.14	10510.11	-1.26922	0.022617
836	ENSMUSG00000027613	Eif6	2575.614	644.0669	1911.935	703.0459	679.4672	875.0869	-1.18389	0.022623
837	ENSMUSG00000001630	Stk38l	435.1037	236.6098	375.7799	125.9883	236.2096	133.8778	-1.07565	0.022636

Table S1. Differentially expressed genes from RNA-seq analysis of db/db mice livers (TAC vs. Veh; p-value < 0.05)										
	Gene_ID	Gene_Name	Veh_1	Veh_2	Veh_3	TAC_1	TAC_2	TAC_3	log2FoldChange	p-value
838	ENSMUSG00000034990	Otoa	0	0	41.29449	0.777706	0	0	-5.63904	0.022698
839	ENSMUSG00000024816	Frmd8	496.7851	301.6945	330.3559	176.5392	214.3384	199.4222	-0.93893	0.022729
840	ENSMUSG00000076612	Ighg2c	14.17004	7.457616	0	0.777706	129.0405	42.5341	2.859665	0.022741
841	ENSMUSG00000109028	Gm35842	0	0	41.29449	0	0	0.69728	-5.63911	0.02275
842	ENSMUSG00000019256	Ahr	513.4557	547.7958	429.4627	954.2449	1240.83	649.168	0.928379	0.022759
843	ENSMUSG00000008874	Clec3a	0	0	33.03559	0	0	0	-6.28248	0.02282
844	ENSMUSG00000009075	Cabp7	0	0	33.03559	0	0	0	-6.28248	0.02282
845	ENSMUSG00000026882	4930568D16Rik	0	0	33.03559	0	0	0	-6.28248	0.02282
846	ENSMUSG00000033633	Clec18a	0	0	33.03559	0	0	0	-6.28248	0.02282
847	ENSMUSG00000045027	Prss22	0	0	33.03559	0	0	0	-6.28248	0.02282
848	ENSMUSG00000062365	Gm4968	0	0	33.03559	0	0	0	-6.28248	0.02282
849	ENSMUSG00000070577	Gm572	0	0	33.03559	0	0	0	-6.28248	0.02282
850	ENSMUSG00000081046	Gm12846	0	0	33.03559	0	0	0	-6.28248	0.02282
851	ENSMUSG00000086366	1700061J23Rik	0	0	33.03559	0	0	0	-6.28248	0.02282
852	ENSMUSG00000089773	Skint1	0	0	33.03559	0	0	0	-6.28248	0.02282
853	ENSMUSG00000095794	Igkv6-17	0	0	33.03559	0	0	0	-6.28248	0.02282
854	ENSMUSG00000102785	Gm2447	0	0	33.03559	0	0	0	-6.28248	0.02282
855	ENSMUSG00000104276	Gm37866	0	0	33.03559	0	0	0	-6.28248	0.02282
856	ENSMUSG00000105660	Gm42975	0	0	33.03559	0	0	0	-6.28248	0.02282
857	ENSMUSG00000110511	Gm45699	0	0	33.03559	0	0	0	-6.28248	0.02282
858	ENSMUSG00000112136	Gm40692	0	0	33.03559	0	0	0	-6.28248	0.02282
859	ENSMUSG00000116122	Gm49537	0	0	33.03559	0	0	0	-6.28248	0.02282
860	ENSMUSG00000024158	Hagh	648.4879	1370.167	1032.362	2157.356	2136.093	1612.112	0.952743	0.02285
861	ENSMUSG00000026159	Agfg1	660.1574	408.135	668.9707	179.65	327.3399	348.6402	-1.01829	0.022911
862	ENSMUSG00000031147	Magix	43.34367	30.50843	78.45953	171.0952	99.87877	89.25189	1.308978	0.022994
863	ENSMUSG00000086782	E130102H24Rik	86.68733	54.23721	441.851	75.43745	51.76199	52.99331	-1.66566	0.023008
864	ENSMUSG00000109783	Gm45338	25.00596	27.1186	28.90614	122.0998	44.47157	55.08515	1.461794	0.023042
865	ENSMUSG00000111092	Gm17875	0.833532	0	33.03559	0	0	0.69728	-5.32711	0.023064
866	ENSMUSG00000114585	Gm32401	0	0.677965	57.81228	0	0	2.091841	-4.71948	0.023109
867	ENSMUSG00000043644	0610009L18Rik	3.334128	8.813546	33.03559	0.777706	1.458084	2.789122	-3.0424	0.023181
868	ENSMUSG00000020331	Hcn2	48.34486	17.62709	70.20063	22.55346	4.374253	6.275523	-2.00454	0.02319

Table S1. Differentially expressed genes from RNA-seq analysis of db/db mice livers (TAC vs. Veh; p-value < 0.05)										
	Gene_ID	Gene_Name	Veh_1	Veh_2	Veh_3	TAC_1	TAC_2	TAC_3	log2FoldChange	p-value
869	ENSMUSG00000068263	Efcc1	3.334128	15.5932	94.97733	3.110823	2.187126	11.85377	-2.65686	0.023193
870	ENSMUSG00000092203	1110038B12Rik	128.3639	86.10157	173.4369	76.99286	34.99402	50.20419	-1.23897	0.023194
871	ENSMUSG00000039395	Mreg	146.7016	329.491	119.754	368.6325	439.6124	510.4092	1.127918	0.023194
872	ENSMUSG00000079057	Cyp4v3	2213.861	2927.453	2580.906	3348.023	6007.307	4851.677	0.879508	0.023239
873	ENSMUSG00000079224	Gm6565	23.3389	45.42366	94.97733	3.888528	23.32935	15.34017	-1.8976	0.023264
874	ENSMUSG00000088609	Gm24187	0	0.677965	33.03559	0.777706	0	0	-5.31945	0.023268
875	ENSMUSG00000063245	Zfp993	24.17243	16.27116	41.29449	8.554762	6.561379	5.578243	-1.92333	0.023341
876	ENSMUSG00000037624	Kcnk2	0.833532	0.677965	45.42394	0.777706	1.458084	0.69728	-3.8815	0.023361
877	ENSMUSG00000071757	Zhx2	282.5674	186.4404	499.6633	69.99351	170.5959	165.9527	-1.23921	0.023425
878	ENSMUSG00000031781	Ciapin1	268.3973	229.1522	144.5307	789.3713	282.8683	381.4124	1.16667	0.023426
879	ENSMUSG00000034007	Scaper	116.6945	128.1354	206.4724	27.9974	67.80092	86.46277	-1.2899	0.023428
880	ENSMUSG00000016194	Hsd11b1	2398.072	6117.279	3423.313	8042.254	8962.115	6965.134	1.005095	0.023465
881	ENSMUSG00000116993	AC135964.2	4.16766	23.72878	16.5178	118.989	40.82636	20.22113	2.029368	0.023507
882	ENSMUSG00000046572	Zfp518b	78.35201	30.50843	115.6246	11.66559	15.30988	41.13954	-1.68759	0.023648
883	ENSMUSG00000009630	Ppp2cb	770.1836	355.2537	825.8898	206.8697	357.9597	355.613	-1.07956	0.023691
884	ENSMUSG00000053746	Ptrh1	37.50894	2.033895	115.6246	10.11017	7.290421	11.15649	-2.3905	0.023725
885	ENSMUSG00000060152	Pop5	228.3878	194.576	82.58898	734.1541	296.7201	237.0753	1.30744	0.02377
886	ENSMUSG00000022865	Cxadr	1161.944	1370.845	1284.259	384.9643	872.6634	700.7668	-0.96261	0.023772
887	ENSMUSG00000050900	Gm7327	11.66945	4.745756	16.5178	0	2.187126	0	-3.86365	0.023815
888	ENSMUSG00000088106	Gm22378	40.00954	7.457616	0	0.777706	1.458084	2.789122	-3.29479	0.023824
889	ENSMUSG00000049357	4933408B17Rik	6.668256	4.067791	57.81228	5.44394	1.458084	2.091841	-2.8261	0.023846
890	ENSMUSG00000037697	Ddhd1	365.087	250.8471	351.0032	146.9864	194.6542	168.0446	-0.91967	0.023857
891	ENSMUSG00000031112	Stk26	6.668256	10.16948	61.94173	2.333117	4.374253	6.275523	-2.49045	0.023923
892	ENSMUSG00000019979	Apaf1	173.3747	56.2711	107.3657	22.55346	51.76199	52.29603	-1.41204	0.024037
893	ENSMUSG00000063714	Sp3os	0.833532	2.033895	0	23.33117	3.645211	12.55105	3.369469	0.02405
894	ENSMUSG00000034456	Uroc1	1708.741	5416.941	1664.168	6955.022	6059.069	6765.014	1.168755	0.024198
895	ENSMUSG00000082029	H3f3c	24.17243	20.33895	61.94173	7.777057	13.8518	6.275523	-1.86027	0.024202
896	ENSMUSG00000025350	Rdh5	125.8633	143.0506	111.4951	271.4193	263.1842	200.1195	0.938232	0.024241
897	ENSMUSG00000097921	Gm26576	2.500596	0.677965	45.42394	2.333117	0.729042	0	-3.90279	0.024317
898	ENSMUSG00000027597	Ahcy	18303.53	14505.06	17232.19	31154.11	30589.88	24045.71	0.777742	0.024335
899	ENSMUSG00000032115	Hyou1	2227.198	486.7789	1812.828	626.0531	660.5122	657.5354	-1.21822	0.024359

Table S1. Differentially expressed genes from RNA-seq analysis of db/db mice livers (TAC vs. Veh; p-value < 0.05)

	Gene_ID	Gene_Name	Veh_1	Veh_2	Veh_3	TAC_1	TAC_2	TAC_3	log2FoldChange	p-value
900	ENSMUSG00000070056	Mfhas1	451.7744	254.9149	454.2394	108.1011	261.7261	167.3473	-1.10714	0.024395
901	ENSMUSG00000110520	Gm45776	4.16766	0.677965	41.29449	2.333117	0	0.69728	-3.83692	0.024431
902	ENSMUSG00000032253	Phip	379.2571	233.898	470.7572	163.3182	176.4282	205.6977	-0.98048	0.024473
903	ENSMUSG00000106106	CT010467.1	1865.445	1182.371	12260.33	1969.151	1078.253	1858.252	-1.64062	0.024494
904	ENSMUSG00000015337	Endog	124.1963	120.6778	28.90614	258.1983	195.3833	220.3406	1.252226	0.024534
905	ENSMUSG00000042029	Ncapg2	26.67303	36.61012	103.2362	10.11017	26.24552	11.15649	-1.75021	0.024612
906	ENSMUSG00000040483	Xaf1	172.5411	119.9998	256.0258	90.99156	75.82038	90.64645	-1.06974	0.024741
907	ENSMUSG00000064325	Hhip	9.168853	13.5593	0	26.44199	43.74253	34.16674	1.982468	0.024743
908	ENSMUSG00000042800	Spata46	30.00715	2.033895	45.42394	0	0	6.972804	-3.42922	0.024766
909	ENSMUSG00000050234	Gja4	38.34247	44.06773	20.64724	92.54697	105.7111	63.45251	1.282891	0.024802
910	ENSMUSG00000023904	Hcfc1r1	134.1987	249.4912	99.10677	611.2767	247.8743	283.0958	1.224575	0.024805
911	ENSMUSG00000082776	Gm7061	4.16766	4.067791	49.55339	0	2.916168	2.789122	-3.22655	0.02485
912	ENSMUSG00000021210	Akr1c6	87.52087	4981.01	1581.579	5918.34	10741.71	8589.797	1.924364	0.024867
913	ENSMUSG00000000673	Haao	851.8698	3723.384	1622.873	5390.278	4655.663	4185.077	1.198193	0.024888
914	ENSMUSG00000035944	Ttc38	354.2511	392.5418	123.8835	462.7349	755.2876	718.8961	1.137113	0.024917
915	ENSMUSG00000010311	Optc	0.833532	0	24.77669	0	0	0	-5.8883	0.024944
916	ENSMUSG00000113610	Gm47132	0.833532	0	24.77669	0	0	0	-5.8883	0.024944
917	ENSMUSG00000061518	Cox5b	415.9325	655.5923	433.5921	1684.51	853.7083	645.6816	1.078021	0.024944
918	ENSMUSG00000015478	Rnf5	102.5244	271.186	86.71843	374.0764	379.1019	308.1979	1.182394	0.025032
919	ENSMUSG00000021365	Nedd9	156.704	71.8643	41.29449	11.66559	28.43264	48.80963	-1.62312	0.025043
920	ENSMUSG00000050856	Atp5k	43.34367	133.5591	49.55339	260.5314	208.506	115.7485	1.34074	0.025108
921	ENSMUSG00000025085	Ablim1	937.7236	484.0671	342.7443	191.3156	282.8683	343.7592	-1.11503	0.025139
922	ENSMUSG00000034591	Slc41a2	2724.816	102.3727	1771.534	505.5087	550.4268	308.1979	-1.75281	0.025189
923	ENSMUSG00000111993	Gm48018	0	0	0	42.77381	0	0	5.867731	0.025214
924	ENSMUSG00000104621	Gm43185	0	2.71186	20.64724	0	0	0	-5.75699	0.02522
925	ENSMUSG00000046159	Chrm3	49.17839	7.457616	115.6246	14.77641	14.58084	12.55105	-1.9865	0.025274
926	ENSMUSG00000094683	Ap3m1-ps	0.833532	6.101686	78.45953	0	5.832337	2.091841	-3.35676	0.025278
927	ENSMUSG00000037525	Bcdin3d	45.01073	37.96605	119.754	18.66494	25.51647	25.10209	-1.48942	0.025323
928	ENSMUSG00000069011	Gm10254	12.50298	10.16948	66.07118	6.221645	7.290421	4.183682	-2.22388	0.025369
929	ENSMUSG00000104973	A530041M06Rik	18.33771	6.779651	49.55339	4.666234	0.729042	6.972804	-2.51431	0.02538
930	ENSMUSG00000106139	Gm30648	0	0.677965	0	9.332468	5.832337	9.761925	4.174242	0.025405

Table S1. Differentially expressed genes from RNA-seq analysis of db/db mice livers (TAC vs. Veh; p-value < 0.05)										
	Gene_ID	Gene_Name	Veh_1	Veh_2	Veh_3	TAC_1	TAC_2	TAC_3	log2FoldChange	p-value
931	ENSMUSG00000070704	Ugt2b36	508.4546	5492.195	1767.404	7955.151	8159.439	5869.706	1.500152	0.02542
932	ENSMUSG00000000753	Serpinf1	1957.967	4877.959	1804.569	5552.818	5894.305	6568.381	1.058682	0.02543
933	ENSMUSG00000045094	Arhgef37	8.335321	69.83041	0	28.77511	207.048	154.099	2.242406	0.025511
934	ENSMUSG00000025272	Tro	0.833532	8.135581	0	11.66559	40.82636	18.82657	2.722932	0.02554
935	ENSMUSG00000067144	Slc22a7	3.334128	65.76262	0	479.8444	231.8354	122.7213	3.556252	0.025574
936	ENSMUSG00000061740	Cyp2d22	1058.586	2807.454	1193.411	4768.113	3011.673	2996.911	1.089087	0.02559
937	ENSMUSG00000031897	Psmb10	213.3842	227.1183	156.9191	538.1723	340.4627	302.6197	0.971486	0.025595
938	ENSMUSG00000028359	Orm3	586.8066	89.49139	982.8088	4632.793	683.1125	658.93	1.853154	0.025617
939	ENSMUSG00000026864	Hspa5	15458.69	3341.012	11735.89	5359.947	4026.5	3850.382	-1.2058	0.025632
940	ENSMUSG00000032437	Stt3b	3382.473	1364.744	2209.255	1283.992	1151.157	1119.135	-0.96895	0.025676
941	ENSMUSG00000041912	Tdrkh	32.50775	51.52535	107.3657	20.99805	22.60031	23.01025	-1.46747	0.025691
942	ENSMUSG00000038754	Elovl3	46.67779	300.3385	99.10677	771.484	414.0959	170.1364	1.592742	0.025706
943	ENSMUSG00000024270	Slc39a6	109.1927	44.06773	74.33008	21.77576	39.36827	26.49665	-1.37599	0.025824
944	ENSMUSG00000096056	Gm21986	0.833532	0.677965	41.29449	0.777706	1.458084	0	-4.15515	0.025845
945	ENSMUSG00000103928	Gm37893	13.33651	31.86436	144.5307	3.110823	16.76797	21.61569	-2.13873	0.025981
946	ENSMUSG00000042102	Dmgdh	2689.808	8602.021	3505.902	8566.428	9621.898	14340.27	1.135785	0.026149
947	ENSMUSG00000028393	Alad	772.6842	990.507	991.0677	2424.886	1110.331	1739.715	0.938451	0.026149
948	ENSMUSG00000092600	Gm20442	5.834724	0	16.5178	0	0	0	-5.71479	0.026153
949	ENSMUSG00000035960	Apex1	124.1963	37.96605	156.9191	24.10888	48.11678	42.5341	-1.44996	0.026239
950	ENSMUSG00000036904	Fzd8	81.68614	65.76262	4.129449	160.2074	163.3054	136.667	1.530624	0.026244
951	ENSMUSG00000022766	Serpind1	731.0076	4114.57	1135.598	4866.882	5539.991	4604.84	1.326108	0.026322
952	ENSMUSG00000094989	Rpl9-ps4	194.213	107.1185	338.6148	97.21321	98.42068	85.06821	-1.1663	0.026322
953	ENSMUSG00000058013	45546	414.2654	216.2709	367.521	79.32598	216.5255	154.7962	-1.14373	0.026343
954	ENSMUSG00000097974	Gm10605	3.334128	10.16948	99.10677	1.555411	10.93563	4.183682	-2.68033	0.026371
955	ENSMUSG00000084923	Gm15611	55.84665	5.423721	0	0	3.645211	2.091841	-3.45316	0.026372
956	ENSMUSG00000037788	Vopp1	65.0155	33.89826	136.2718	10.88788	43.01348	18.12929	-1.66771	0.026388
957	ENSMUSG00000083991	Gm11618	1.667064	0	28.90614	0.777706	0	0	-5.17219	0.026413
958	ENSMUSG00000037949	Ano10	680.1622	282.7115	441.851	217.7576	223.0869	257.9937	-1.00798	0.026446
959	ENSMUSG00000044646	Zbtb7c	39.17601	4.745756	16.5178	0.777706	0	6.275523	-3.09566	0.026464
960	ENSMUSG00000044080	S100a1	401.7624	729.4905	437.7216	1825.275	902.5541	664.5082	1.109362	0.026513
961	ENSMUSG00000045008	9030612E09Rik	0	0	165.178	0	0	0	-8.66581	0.026662

Table S1. Differentially expressed genes from RNA-seq analysis of db/db mice livers (TAC vs. Veh; p-value < 0.05)										
	Gene_ID	Gene_Name	Veh_1	Veh_2	Veh_3	TAC_1	TAC_2	TAC_3	log2FoldChange	p-value
962	ENSMUSG00000021629	Slc30a5	1322.815	387.1181	1379.236	575.5022	436.6962	357.0076	-1.17116	0.026664
963	ENSMUSG00000043467	Zbtb37	149.2022	181.0167	94.97733	383.4089	142.8923	467.8751	1.208845	0.026671
964	ENSMUSG00000048988	Elfn1	7.501788	0	74.33008	0	2.187126	4.183682	-3.6144	0.026717
965	ENSMUSG00000034471	Caskin2	1181.115	169.4913	445.9805	278.4186	217.9836	188.963	-1.39246	0.02672
966	ENSMUSG00000028619	Tceanc2	2.500596	25.08471	103.2362	7.777057	10.20659	6.275523	-2.36619	0.026731
967	ENSMUSG00000062542	Syt9	3.334128	18.30506	107.3657	0.777706	7.290421	11.85377	-2.63139	0.026743
968	ENSMUSG00000071177	Serpina1d	7265.065	12949.81	9101.305	12476.73	25561.67	18805.65	0.955215	0.026762
969	ENSMUSG00000060923	Acyp2	14.17004	27.1186	119.754	17.88723	14.58084	6.972804	-1.96315	0.026775
970	ENSMUSG00000104040	Gm37563	1.667064	0	41.29449	0	1.458084	0	-4.7933	0.026833
971	ENSMUSG00000034574	Daam1	249.2261	509.8298	194.0841	559.1704	639.3699	797.6888	1.054431	0.027031
972	ENSMUSG00000089936	Gm16199	3.334128	3.389826	45.42394	2.333117	2.187126	1.394561	-3.00814	0.027047
973	ENSMUSG00000035969	Rusc2	334.2464	292.203	206.4724	671.16	489.1873	426.7356	0.918875	0.027062
974	ENSMUSG00000030108	Slc6a13	1243.63	2167.454	1214.058	3813.869	2133.177	2989.938	0.948498	0.027107
975	ENSMUSG00000027577	Chrna4	14.17004	85.4236	0	0	6.561379	5.578243	-3.06783	0.027161
976	ENSMUSG00000078920	Ifi47	590.9742	134.9151	351.0032	181.2054	145.0794	129.6942	-1.23987	0.027196
977	ENSMUSG00000024036	Slc37a1	477.6139	12.88134	396.4271	139.987	50.30391	16.73473	-2.0966	0.027352
978	ENSMUSG00000017737	Mmp9	16.67064	8.813546	144.5307	8.554762	16.76797	0	-2.70569	0.027384
979	ENSMUSG00000085148	Mir22hg	491.7839	252.881	569.864	1437.978	491.3744	959.4578	1.141781	0.027432
980	ENSMUSG00000003354	Ccdc65	4.16766	0	41.29449	1.555411	0.729042	0.69728	-3.83178	0.027452
981	ENSMUSG00000022615	Tymp	484.2821	1463.727	532.6989	1727.284	1619.203	2015.838	1.10805	0.027491
982	ENSMUSG00000068196	Col8a1	33.34128	6.779651	4.129449	0	2.916168	2.091841	-3.18302	0.027517
983	ENSMUSG00000041361	Myzap	2.500596	23.05081	90.84788	1.555411	13.8518	2.091841	-2.6769	0.02752
984	ENSMUSG00000055471	Alk	0	1.35593	61.94173	0	2.916168	0.69728	-4.05413	0.027573
985	ENSMUSG00000027562	Car2	33.34128	146.4405	24.77669	207.6474	182.2605	190.3575	1.459302	0.027634
986	ENSMUSG00000020263	Appl2	520.124	646.1007	673.1002	375.6318	278.4941	377.2287	-0.83232	0.027698
987	ENSMUSG00000108955	Gm44775	0	3.389826	37.16504	1.555411	0	0	-4.65011	0.027702
988	ENSMUSG00000026117	Zap70	23.3389	33.89826	57.81228	90.99156	88.94314	78.0954	1.245109	0.027732
989	ENSMUSG00000004071	Cdip1	1155.275	1210.846	1197.54	3677.77	1813.857	1520.769	0.976558	0.027754
990	ENSMUSG00000112640	Gm32687	0	1.35593	4.129449	15.55411	11.66467	8.367365	3.280264	0.02781
991	ENSMUSG00000019302	Atp6v0a1	2058.824	705.7617	1296.647	620.6091	511.0585	819.3044	-1.05781	0.027854
992	ENSMUSG00000043635	Adamts3	1.667064	8.135581	57.81228	1.555411	4.374253	2.789122	-2.84664	0.027871

Table S1. Differentially expressed genes from RNA-seq analysis of db/db mice livers (TAC vs. Veh; p-value < 0.05)										
	Gene_ID	Gene_Name	Veh_1	Veh_2	Veh_3	TAC_1	TAC_2	TAC_3	log2FoldChange	p-value
993	ENSMUSG00000109901	Chmp1b	455.942	475.9315	1024.103	361.6331	333.1722	269.1502	-1.0117	0.027893
994	ENSMUSG00000032300	1700017B05Rik	525.1252	223.0505	243.6375	91.76927	197.5704	158.9799	-1.15104	0.027922
995	ENSMUSG00000091898	Tnnc1	5.834724	3.389826	4.129449	33.44134	29.16168	6.275523	2.352268	0.027943
996	ENSMUSG00000074635	3110070M22Rik	8.335321	1.35593	16.5178	0	0.729042	0.69728	-4.08608	0.027995
997	ENSMUSG00000026827	Gpd2	640.1526	2010.845	491.4044	335.9688	482.6259	515.9875	-1.23916	0.028
998	ENSMUSG00000020672	Sntg2	31.67422	48.81349	0	108.1011	101.3369	69.03076	1.67876	0.028005
999	ENSMUSG00000021565	Slc6a19	0	3.389826	0	30.33052	3.645211	13.94561	3.437059	0.028091
1000	ENSMUSG00000022108	Itm2b	5072.876	11785.07	5025.539	15026.05	14768.21	13511.2	0.984198	0.028156
1001	ENSMUSG00000020681	Ace	16.67064	25.08471	210.6019	13.221	21.87126	26.49665	-1.98242	0.02817
1002	ENSMUSG00000038704	Aspdh	179.2094	580.3381	218.8608	1055.347	676.5511	527.144	1.199113	0.028215
1003	ENSMUSG00000025533	Asl	2840.677	7178.973	4162.485	15621.77	8536.354	6321.544	1.1035	0.028218
1004	ENSMUSG00000003824	Syce2	40.84307	86.10157	70.20063	172.6507	131.2276	123.4186	1.121152	0.028223
1005	ENSMUSG00000033326	Kdm4a	706.0016	350.508	528.5695	204.5366	313.4881	293.555	-0.9652	0.028247
1006	ENSMUSG00000038764	Ptpn3	1031.913	399.9994	958.0322	301.7498	451.2771	407.909	-1.03882	0.028258
1007	ENSMUSG00000030604	Zfp626	63.34844	78.64395	161.0485	30.33052	37.18115	53.69059	-1.28293	0.028312
1008	ENSMUSG00000080972	Gm16061	4.16766	3.389826	0	52.10628	1.458084	16.73473	2.977739	0.028407
1009	ENSMUSG00000038370	Pcp4l1	4.16766	60.33889	41.29449	76.21516	63.42666	244.0481	1.869117	0.028474
1010	ENSMUSG00000029816	Gpnmb	33.34128	95.59308	0	1.555411	3.645211	16.03745	-2.63329	0.028497
1011	ENSMUSG00000074398	Gm15441	174.2082	11.52541	66.07118	34.99676	18.95509	13.94561	-1.89813	0.028623
1012	ENSMUSG00000029784	Ssmem1	0.833532	1.35593	33.03559	0	1.458084	0	-4.47557	0.028663
1013	ENSMUSG00000067219	Nipal1	180.0429	444.7451	317.9676	82.4368	171.3249	174.3201	-1.13874	0.028664
1014	ENSMUSG00000020776	Fbf1	496.7851	207.4573	239.508	1111.341	211.4222	1142.145	1.381254	0.028672
1015	ENSMUSG00000014905	Dnajb9	1138.605	545.0839	1300.776	374.0764	565.0076	550.1542	-0.99933	0.028699
1016	ENSMUSG00000040128	Pnrc1	3547.512	382.3723	2366.174	1125.34	710.087	427.4329	-1.47599	0.028792
1017	ENSMUSG00000024863	Mbl2	496.7851	2035.929	1019.974	2676.085	3204.14	2046.518	1.156675	0.028909
1018	ENSMUSG00000106229	Gm19409	5.834724	0.677965	28.90614	1.555411	0.729042	0	-3.85884	0.028942
1019	ENSMUSG00000025372	Baiap2	70.85022	444.0671	123.8835	1144.783	292.3459	442.773	1.547757	0.029006
1020	ENSMUSG00000046861	Hectd3	373.4224	532.2026	202.343	916.915	572.2981	779.5595	1.021641	0.029006
1021	ENSMUSG00000032840	2410131K14Rik	62.5149	52.88128	20.64724	106.5457	121.75	100.4084	1.20412	0.029036
1022	ENSMUSG00000030157	Clec2d	35.84188	195.9319	132.1424	402.8515	407.5345	155.4935	1.410461	0.029157
1023	ENSMUSG00000064927	Gm22043	0	1.35593	0	32.66364	2.916168	1.394561	4.098652	0.029233

Table S1. Differentially expressed genes from RNA-seq analysis of db/db mice livers (TAC vs. Veh; p-value < 0.05)										
	Gene_ID	Gene_Name	Veh_1	Veh_2	Veh_3	TAC_1	TAC_2	TAC_3	log2FoldChange	p-value
1024	ENSMUSG00000116506	5730414N17Rik	11.66945	24.40674	16.5178	87.88074	30.61977	42.5341	1.598461	0.029242
1025	ENSMUSG00000013707	Tnfaip8l2	25.00596	13.5593	99.10677	12.44329	16.03893	4.183682	-2.00623	0.029245
1026	ENSMUSG00000026032	Ndufb3	162.5388	249.4912	189.9547	597.278	368.8953	260.7829	1.023951	0.029314
1027	ENSMUSG00000092919	Gm24080	0	0	37.16504	0.777706	0	0	-5.48238	0.029403
1028	ENSMUSG00000108059	Gm44369	0	0	37.16504	0.777706	0	0	-5.48238	0.029403
1029	ENSMUSG00000066861	Oas1g	1.667064	3.389826	0	27.9974	5.832337	13.24833	2.908106	0.029423
1030	ENSMUSG00000041534	Rbp3	0	0	37.16504	0	0.729042	0	-5.48244	0.029446
1031	ENSMUSG00000105542	Gm34248	0	0	37.16504	0	0	0.69728	-5.48247	0.029474
1032	ENSMUSG00000116628	AC098883.2	0	0	37.16504	0	0	0.69728	-5.48247	0.029474
1033	ENSMUSG00000081121	Gm12791	0.833532	1.35593	16.5178	0	0	0	-5.40097	0.029491
1034	ENSMUSG00000036473	Tbc1d24	629.3167	421.0163	495.5339	232.534	291.6168	327.7218	-0.86027	0.029676
1035	ENSMUSG00000041440	Gk5	7.501788	6.779651	53.68284	0	0.729042	6.972804	-3.05165	0.0297
1036	ENSMUSG00000023991	Foxp4	871.041	275.2538	561.6051	363.9663	185.9057	225.2216	-1.13984	0.029721
1037	ENSMUSG00000063754	Gm10136	2.500596	2.033895	94.97733	6.999351	2.187126	3.486402	-2.89788	0.029728
1038	ENSMUSG00000021606	Ndufs6	267.5638	378.9825	351.0032	967.4659	566.4657	430.9193	0.979308	0.029756
1039	ENSMUSG00000025481	Urah	881.0434	853.5581	887.8315	2983.279	1216.042	1142.843	1.026631	0.029778
1040	ENSMUSG00000021838	Samd4	77.51848	62.37279	206.4724	23.33117	32.80689	67.6362	-1.4481	0.029809
1041	ENSMUSG00000091721	Gimd1	105.8586	48.13552	111.4951	35.77446	40.09732	35.5613	-1.2313	0.029834
1042	ENSMUSG00000023952	Gtpbp2	1011.074	463.7281	1114.951	342.1905	407.5345	538.3005	-1.00381	0.030037
1043	ENSMUSG00000022663	Atg3	391.7601	767.4565	466.6277	1429.423	877.7667	868.1141	0.962438	0.030082
1044	ENSMUSG00000025198	Erlin1	851.0362	446.779	830.0192	258.1983	447.6319	383.5042	-0.96287	0.030222
1045	ENSMUSG00000055312	Them7	202.5483	328.1351	123.8835	410.6286	513.2456	414.1845	1.011906	0.030227
1046	ENSMUSG00000031099	Smarca1	0	2.033895	61.94173	0	2.187126	2.091841	-3.81855	0.030248
1047	ENSMUSG00000093651	Gm5873	77.51848	49.49145	103.2362	0.777706	16.03893	39.74498	-2.0099	0.030264
1048	ENSMUSG00000038286	Bphl	260.062	1453.557	528.5695	1643.292	1775.947	1950.991	1.257171	0.030295
1049	ENSMUSG00000109141	Gm30692	8.335321	74.57616	16.5178	4.666234	11.66467	6.275523	-2.17128	0.030318
1050	ENSMUSG00000067869	Tcea1-ps1	67.5161	47.45756	144.5307	13.9987	40.82636	36.95586	-1.45908	0.030386
1051	ENSMUSG00000030237	Slco1a4	137.5328	1854.235	388.1682	2291.121	3097.7	1764.119	1.584669	0.030409
1052	ENSMUSG00000039783	Kmo	2270.541	3375.588	2597.423	3940.635	6495.036	4399.839	0.847382	0.030411
1053	ENSMUSG00000003849	Nqo1	100.8574	134.9151	78.45953	181.2054	165.4926	316.5653	1.057372	0.030523
1054	ENSMUSG00000026074	Map4k4	442.6055	239.3217	408.8154	110.4342	178.6153	240.5617	-1.03817	0.030525

Table S1. Differentially expressed genes from RNA-seq analysis of db/db mice livers (TAC vs. Veh; p-value < 0.05)										
	Gene_ID	Gene_Name	Veh_1	Veh_2	Veh_3	TAC_1	TAC_2	TAC_3	log2FoldChange	p-value
1055	ENSMUSG00000006784	Ttc25	6.668256	56.94907	0	3.888528	1.458084	3.486402	-2.90945	0.030539
1056	ENSMUSG00000106664	Gm17936	2.500596	4.745756	0	24.10888	19.68414	9.064645	2.574814	0.030682
1057	ENSMUSG00000027187	Cat	4849.489	24636.57	7581.668	24661.82	31531.07	31595.87	1.243642	0.030751
1058	ENSMUSG00000031711	Zfp330	246.7255	187.1184	317.9676	115.1004	116.6467	156.1908	-0.94157	0.030804
1059	ENSMUSG00000031824	6430548M08Rik	123.3627	86.77953	169.3074	34.21905	80.19463	49.50691	-1.19055	0.030887
1060	ENSMUSG00000032643	Fhl3	196.7136	33.22029	82.58898	20.22035	24.78743	57.87427	-1.60786	0.030902
1061	ENSMUSG00000024810	Il33	61.68137	69.15244	37.16504	10.88788	29.16168	23.01025	-1.43988	0.030925
1062	ENSMUSG00000024414	Mrpl27	282.5674	270.5081	161.0485	959.6888	341.1917	318.6571	1.171258	0.030957
1063	ENSMUSG00000028238	Atp6v0d2	0	52.20331	16.5178	54.4394	56.86528	240.5617	2.331224	0.030983
1064	ENSMUSG00000115431	Gm3219	5.001192	20.33895	61.94173	253.532	62.69762	22.31297	2.049489	0.030999
1065	ENSMUSG00000069922	Ces3a	818.5285	7339.65	1639.391	11910.56	11537.09	4629.244	1.518131	0.031061
1066	ENSMUSG00000039496	Cdnf	30.84069	15.5932	41.29449	4.666234	10.93563	8.367365	-1.82389	0.031125
1067	ENSMUSG00000032842	Abcc10	180.0429	202.0336	99.10677	531.9507	199.0285	325.6299	1.115931	0.031128
1068	ENSMUSG00000002059	Rab34	217.5519	132.2032	417.0743	164.8736	60.5105	92.04101	-1.25653	0.031162
1069	ENSMUSG00000022261	Sdc2	912.7176	1202.032	751.5597	1697.731	1728.559	1560.513	0.795184	0.031164
1070	ENSMUSG00000001435	Col18a1	5795.548	7503.718	4988.374	10565.13	9441.095	11132.08	0.767277	0.031236
1071	ENSMUSG00000019590	Cyb561	916.8853	77.96599	573.9934	272.9747	163.3054	90.64645	-1.57324	0.031257
1072	ENSMUSG00000025151	Maged1	2259.705	1401.354	1296.647	822.0349	1173.758	559.2189	-0.95777	0.031333
1073	ENSMUSG00000022126	Acod1	21.67183	13.5593	0	0	2.187126	0	-4.06345	0.031369
1074	ENSMUSG00000095193	Gm20939	20.00477	12.88134	16.5178	1.555411	3.645211	4.880963	-2.28441	0.031373
1075	ENSMUSG00000039989	Cbx4	103.358	84.74564	111.4951	202.2035	164.0345	195.2385	0.919399	0.031498
1076	ENSMUSG00000040124	Gorab	75.85142	65.08465	74.33008	17.88723	34.26498	38.35042	-1.24472	0.031652
1077	ENSMUSG00000007833	Aldh16a1	502.6198	479.9993	561.6051	1095.01	716.6484	877.1787	0.80283	0.031694
1078	ENSMUSG00000021133	Susd6	2474.757	1053.558	1944.97	897.4723	1137.306	820.699	-0.93814	0.031755
1079	ENSMUSG00000026142	Rhbdd1	660.9909	234.5759	293.1909	157.8743	201.2156	204.3032	-1.08221	0.031762
1080	ENSMUSG00000104266	Gm37748	0.833532	0	28.90614	0	0.729042	0	-5.13204	0.031863
1081	ENSMUSG00000028076	Cd1d1	2508.931	3735.588	2960.815	5957.225	4182.515	5928.975	0.803481	0.031928
1082	ENSMUSG00000022408	Fam83f	46.67779	23.05081	8.258898	136.0985	27.7036	96.92197	1.666575	0.031929
1083	ENSMUSG00000085776	5430402O13Rik	0.833532	4.745756	0	29.55282	8.748505	11.85377	2.858164	0.031945
1084	ENSMUSG00000027954	Efna1	1677.066	185.7624	2262.938	878.8074	188.0929	214.7624	-1.68503	0.031946
1085	ENSMUSG00000028070	Naxe	310.9075	467.118	322.097	692.9357	616.7696	641.4979	0.82142	0.03195

Table S1. Differentially expressed genes from RNA-seq analysis of db/db mice livers (TAC vs. Veh; p-value < 0.05)										
	Gene_ID	Gene_Name	Veh_1	Veh_2	Veh_3	TAC_1	TAC_2	TAC_3	log2FoldChange	p-value
1086	ENSMUSG00000011114	Tbrg1	1016.909	355.9317	714.3947	408.2955	281.4103	333.3	-1.02818	0.032109
1087	ENSMUSG00000110750	Gm48702	0	1.35593	0	21.77576	6.561379	2.789122	3.847753	0.032122
1088	ENSMUSG00000035293	G2e3	56.68018	107.1185	136.2718	38.88528	51.76199	39.0477	-1.18522	0.032124
1089	ENSMUSG00000109498	Gm45222	8.335321	7.457616	99.10677	12.44329	0	3.486402	-2.79718	0.032134
1090	ENSMUSG00000067389	Gm17080	0.833532	21.69488	0	66.10498	25.51647	40.44226	2.377889	0.032159
1091	ENSMUSG00000020841	Cpd	1077.757	541.0162	863.0548	403.6292	470.9612	471.3615	-0.88179	0.032178
1092	ENSMUSG00000056529	Ptafr	5.834724	18.98302	53.68284	0.777706	8.748505	4.183682	-2.43956	0.032215
1093	ENSMUSG00000052435	Cebpe	54.17958	75.25413	53.68284	123.6552	114.4596	136.667	1.015752	0.032242
1094	ENSMUSG00000004939	Nmrk2	0	0	28.90614	0	0	0	-6.08541	0.032261
1095	ENSMUSG00000027517	Ankrd60	0	0	28.90614	0	0	0	-6.08541	0.032261
1096	ENSMUSG00000028226	Mmp16	0	0	28.90614	0	0	0	-6.08541	0.032261
1097	ENSMUSG00000034818	Celf5	0	0	28.90614	0	0	0	-6.08541	0.032261
1098	ENSMUSG00000041255	Tmco5b	0	0	28.90614	0	0	0	-6.08541	0.032261
1099	ENSMUSG00000047021	Cfap65	0	0	28.90614	0	0	0	-6.08541	0.032261
1100	ENSMUSG00000047678	Gpr82	0	0	28.90614	0	0	0	-6.08541	0.032261
1101	ENSMUSG00000064394	Gm23622	0	0	28.90614	0	0	0	-6.08541	0.032261
1102	ENSMUSG00000068117	Mei1	0	0	28.90614	0	0	0	-6.08541	0.032261
1103	ENSMUSG00000087055	Gm11948	0	0	28.90614	0	0	0	-6.08541	0.032261
1104	ENSMUSG00000087540	Gm11412	0	0	28.90614	0	0	0	-6.08541	0.032261
1105	ENSMUSG00000096030	Gm24424	0	0	28.90614	0	0	0	-6.08541	0.032261
1106	ENSMUSG00000097672	Gm26609	0	0	28.90614	0	0	0	-6.08541	0.032261
1107	ENSMUSG00000097986	Gm26953	0	0	28.90614	0	0	0	-6.08541	0.032261
1108	ENSMUSG00000099922	Gm29016	0	0	28.90614	0	0	0	-6.08541	0.032261
1109	ENSMUSG00000100359	Gm21766	0	0	28.90614	0	0	0	-6.08541	0.032261
1110	ENSMUSG00000100596	Gm29502	0	0	28.90614	0	0	0	-6.08541	0.032261
1111	ENSMUSG00000101528	Gm29110	0	0	28.90614	0	0	0	-6.08541	0.032261
1112	ENSMUSG00000103391	Gm38302	0	0	28.90614	0	0	0	-6.08541	0.032261
1113	ENSMUSG00000104976	Gm43060	0	0	28.90614	0	0	0	-6.08541	0.032261
1114	ENSMUSG00000106801	Gm42852	0	0	28.90614	0	0	0	-6.08541	0.032261
1115	ENSMUSG00000107334	Gm7538	0	0	28.90614	0	0	0	-6.08541	0.032261
1116	ENSMUSG00000108056	Gm43941	0	0	28.90614	0	0	0	-6.08541	0.032261

Table S1. Differentially expressed genes from RNA-seq analysis of db/db mice livers (TAC vs. Veh; p-value < 0.05)

	Gene_ID	Gene_Name	Veh_1	Veh_2	Veh_3	TAC_1	TAC_2	TAC_3	log2FoldChange	p-value
1117	ENSMUSG00000109363	Gm44668	0	0	28.90614	0	0	0	-6.08541	0.032261
1118	ENSMUSG00000109370	Gm44675	0	0	28.90614	0	0	0	-6.08541	0.032261
1119	ENSMUSG00000111378	Gm47230	0	0	28.90614	0	0	0	-6.08541	0.032261
1120	ENSMUSG00000111781	AC073947.1	0	0	28.90614	0	0	0	-6.08541	0.032261
1121	ENSMUSG00000115117	Gm18094	0	0	28.90614	0	0	0	-6.08541	0.032261
1122	ENSMUSG00000115613	Gm48982	0	0	28.90614	0	0	0	-6.08541	0.032261
1123	ENSMUSG00000116056	Gm4544	0	0	28.90614	0	0	0	-6.08541	0.032261
1124	ENSMUSG00000116348	Gm18722	0	0	28.90614	0	0	0	-6.08541	0.032261
1125	ENSMUSG00000020149	Rab1a	2693.976	2521.352	2601.553	1109.008	1813.857	1601.653	-0.78878	0.032298
1126	ENSMUSG00000061119	Prcp	315.0751	104.4066	235.3786	121.3221	102.0659	69.72804	-1.15668	0.032336
1127	ENSMUSG00000006517	Mvd	671.8268	210.8471	590.5112	64.54957	61.96858	376.5314	-1.54767	0.032357
1128	ENSMUSG00000083097	Gm14494	119.1951	65.08465	74.33008	23.33117	34.99402	50.90147	-1.25049	0.032359
1129	ENSMUSG00000108206	Gm44427	21.67183	4.745756	24.77669	4.666234	1.458084	2.789122	-2.47707	0.032408
1130	ENSMUSG00000067212	H2-T23	761.8483	1235.93	1015.844	2169.799	1461	1739.017	0.833347	0.032416
1131	ENSMUSG00000022070	Bora	10.83592	14.23727	0	59.10563	19.68414	32.77218	1.958275	0.03243
1132	ENSMUSG00000079293	Clec7a	9.168853	132.8812	156.9191	4.666234	18.95509	47.41507	-2.05787	0.032432
1133	ENSMUSG00000015533	Itga2	196.7136	13.5593	272.5436	87.10303	18.95509	6.275523	-2.0948	0.032507
1134	ENSMUSG00000040048	Ndufb10	441.772	901.0156	450.1099	1727.284	972.5422	924.5938	1.011028	0.032559
1135	ENSMUSG00000024019	Cmtr1	13.33651	20.33895	0	66.10498	32.80689	35.5613	1.818756	0.032758
1136	ENSMUSG00000038692	Hoxb4	4.16766	12.88134	198.2135	23.33117	10.20659	6.275523	-2.39083	0.032763
1137	ENSMUSG00000069495	Epc2	264.2297	184.4065	445.9805	129.0991	142.1632	168.0446	-1.00904	0.032782
1138	ENSMUSG00000057835	Zfp119a	72.51729	60.33889	152.7896	13.9987	47.38774	43.23138	-1.41492	0.032819
1139	ENSMUSG00000023393	Slc17a9	137.5328	77.28802	260.1553	75.43745	73.63325	53.69059	-1.19922	0.03291
1140	ENSMUSG00000081179	Gm13136	5.834724	11.52541	78.45953	0	2.916168	10.45921	-2.76728	0.033036
1141	ENSMUSG00000031481	Tpte	2.500596	4.067791	0	14.77641	32.07785	5.578243	2.712791	0.033097
1142	ENSMUSG00000032575	Manf	1927.126	217.6268	2366.174	986.9085	196.1123	237.7726	-1.66541	0.033164
1143	ENSMUSG00000068697	Myoz1	0	1.35593	20.64724	0	0	0	-5.66268	0.033249
1144	ENSMUSG00000073985	Gm10602	2.500596	7.457616	103.2362	3.888528	10.93563	2.789122	-2.60947	0.033276
1145	ENSMUSG00000050627	Gpd1l	172.5411	349.152	177.5663	377.1872	593.4403	426.7356	0.988017	0.033305
1146	ENSMUSG00000103662	Gm34294	3.334128	2.71186	45.42394	1.555411	0.729042	3.486402	-3.02432	0.033312
1147	ENSMUSG00000033938	Ndufb7	333.4128	500.3383	379.9093	1820.609	548.9687	447.654	1.213593	0.033407

Table S1. Differentially expressed genes from RNA-seq analysis of db/db mice livers (TAC vs. Veh; p-value < 0.05)										
	Gene_ID	Gene_Name	Veh_1	Veh_2	Veh_3	TAC_1	TAC_2	TAC_3	log2FoldChange	p-value
1148	ENSMUSG00000038530	Rgs4	21.67183	39.32198	20.64724	69.99351	82.38176	53.69059	1.291238	0.033415
1149	ENSMUSG00000008348	Ubc	743.5106	999.3206	689.618	1974.595	996.6006	1534.714	0.886918	0.033493
1150	ENSMUSG00000003068	Stk11	716.8376	566.1009	503.7928	1556.967	821.6305	941.3285	0.890647	0.03359
1151	ENSMUSG00000102153	Gm37474	0	0	243.6375	0.777706	0	0	-8.30599	0.033592
1152	ENSMUSG00000022877	Hrg	13902.48	8424.394	9625.745	17880.23	17128.12	20143.04	0.787335	0.033623
1153	ENSMUSG00000028124	Gclm	1539.534	2275.929	1482.472	3337.913	2574.248	3321.844	0.800012	0.033669
1154	ENSMUSG00000024535	Snx24	220.886	125.4235	384.0388	122.8775	71.44613	131.0887	-1.14785	0.0338
1155	ENSMUSG00000051934	Spats2	200.0477	98.30494	235.3786	66.10498	99.14973	84.37093	-1.0799	0.03382
1156	ENSMUSG00000029470	P2rx4	1386.164	694.9142	1127.34	640.0518	551.1558	569.6781	-0.86473	0.033861
1157	ENSMUSG00000031613	Hpgd	100.0238	720.6769	487.275	608.9435	1520.053	1243.251	1.367442	0.033911
1158	ENSMUSG00000064340	mt-Tl1	70.01669	28.47453	1015.844	171.0952	38.63923	48.80963	-2.09731	0.033941
1159	ENSMUSG00000064294	Aox3	10.00238	370.8469	99.10677	991.5747	333.1722	400.9362	1.838244	0.034081
1160	ENSMUSG00000020656	Grhl1	27.50656	10.84744	33.03559	3.110823	5.832337	8.367365	-1.99595	0.034135
1161	ENSMUSG00000089682	Bcl2l2	354.2511	250.8471	479.0161	131.4323	196.1123	226.6161	-0.95779	0.034218
1162	ENSMUSG00000056812	St8sia3	31.67422	14.23727	33.03559	4.666234	8.748505	8.367365	-1.82432	0.034304
1163	ENSMUSG00000026452	Syt2	0	2.033895	24.77669	0	0.729042	0	-4.9758	0.034315
1164	ENSMUSG00000079419	Ms4a6c	0	33.22029	152.7896	5.44394	16.76797	6.972804	-2.63623	0.034327
1165	ENSMUSG00000022244	Amacr	985.2349	1064.405	912.6082	1538.302	1938.523	1480.326	0.741411	0.034516
1166	ENSMUSG00000032051	Fdx1	271.7314	448.1349	425.3332	1120.674	617.4987	511.1065	0.976484	0.034517
1167	ENSMUSG00000071551	Akr1c19	109.1927	757.965	355.1326	479.0667	1195.629	1557.027	1.400723	0.034576
1168	ENSMUSG00000025403	Shmt2	1655.395	2227.115	920.8671	3330.913	2606.326	3114.751	0.910945	0.034629
1169	ENSMUSG00000034748	Sirt6	85.02027	44.7457	173.4369	55.2171	26.97456	32.0749	-1.37049	0.03468
1170	ENSMUSG00000032204	Aqp9	2497.262	3983.045	3039.274	4561.244	5849.834	6041.237	0.789063	0.034718
1171	ENSMUSG00000028461	Ccdc107	188.3782	191.1862	483.1455	1469.864	384.9342	338.181	1.358536	0.034732
1172	ENSMUSG00000003500	Impdh1	95.85619	31.1864	111.4951	26.44199	21.87126	40.44226	-1.39858	0.034753
1173	ENSMUSG00000005220	Corin	8.335321	123.3896	0	7.777057	3.645211	11.15649	-2.57903	0.03477
1174	ENSMUSG00000106891	Gm43460	2.500596	2.71186	41.29449	0	1.458084	2.789122	-3.32244	0.034933
1175	ENSMUSG00000035184	Fam124a	175.8753	339.6605	165.178	123.6552	115.1887	105.2893	-0.99652	0.034986
1176	ENSMUSG00000026956	Uap1l1	265.0632	162.7116	194.0841	125.2106	95.50452	106.6839	-0.9285	0.035032
1177	ENSMUSG00000116004	Gm49539	4.16766	2.033895	90.84788	6.221645	5.103295	0	-3.03815	0.035083
1178	ENSMUSG00000032372	Plscr2	95.02265	508.4738	70.20063	503.1756	742.1649	582.2291	1.424376	0.035122

Table S1. Differentially expressed genes from RNA-seq analysis of db/db mice livers (TAC vs. Veh; p-value < 0.05)										
	Gene_ID	Gene_Name	Veh_1	Veh_2	Veh_3	TAC_1	TAC_2	TAC_3	log2FoldChange	p-value
1179	ENSMUSG00000083695	Rnf138rt1	25.00596	0	0	0	0	0	-5.98248	0.035215
1180	ENSMUSG00000054612	Mgmt	79.18554	184.4065	16.5178	315.7485	212.1513	237.7726	1.409356	0.03527
1181	ENSMUSG00000108486	Gm44836	4.16766	1.35593	41.29449	0	2.187126	2.091841	-3.32708	0.035355
1182	ENSMUSG00000031931	Ankrd49	143.3675	107.7965	276.6731	86.32533	72.90421	86.46277	-1.07527	0.035376
1183	ENSMUSG00000071356	Reg3b	11.66945	0	12.38835	0.777706	0	0	-4.89187	0.035493
1184	ENSMUSG00000070394	Tmem256	245.892	148.4744	214.7313	1054.569	283.5974	191.0548	1.329678	0.035494
1185	ENSMUSG00000000724	Cryba1	0	0	49.55339	1.555411	0	0	-4.95855	0.035611
1186	ENSMUSG00000002791	Pdxk-ps	0	0	49.55339	1.555411	0	0	-4.95855	0.035611
1187	ENSMUSG00000022751	Nit2	271.7314	957.9647	569.864	1527.414	1227.707	1011.057	1.064314	0.03565
1188	ENSMUSG00000107451	Gm44421	0	18.98302	0	55.99481	36.45211	31.37762	2.530413	0.03581
1189	ENSMUSG00000027879	Sec22b	1162.777	729.4905	830.0192	581.7238	522.7232	449.7458	-0.80995	0.035824
1190	ENSMUSG00000086447	Gm13522	5.001192	2.033895	0	16.33182	20.41318	11.85377	2.520679	0.035932
1191	ENSMUSG00000066154	Mup3	1727.912	4972.874	3353.113	9722.099	12228.22	2355.413	1.273497	0.035975
1192	ENSMUSG00000021192	Golga5	795.1896	387.1181	1387.495	514.0634	372.5405	319.3544	-1.08542	0.036003
1193	ENSMUSG00000085375	Gm12506	0	0	119.754	0	0	0	-8.19964	0.03603
1194	ENSMUSG00000032289	Thsd4	9.168853	31.86436	128.0129	6.221645	11.66467	23.01025	-1.98619	0.036036
1195	ENSMUSG00000085707	Gm12212	16.67064	3.389826	20.64724	0	2.187126	2.789122	-2.97004	0.03623
1196	ENSMUSG00000096942	Rps19-ps6	57.51371	23.05081	289.0614	37.32987	34.26498	40.44226	-1.68289	0.036484
1197	ENSMUSG00000049878	Rlf	1030.246	216.9488	788.7247	349.1898	267.5585	287.9768	-1.16818	0.03651
1198	ENSMUSG00000027902	Chil6	1.667064	0	37.16504	1.555411	0	0	-4.58325	0.036579
1199	ENSMUSG00000103260	Gm8146	0.833532	0.677965	16.5178	0	0	0	-5.34417	0.036628
1200	ENSMUSG00000021497	Txndc15	445.1061	677.2871	417.0743	1071.678	1019.201	702.1613	0.854316	0.036642
1201	ENSMUSG00000068264	Ap5s1	506.7875	237.2878	355.1326	241.8665	155.286	165.9527	-0.96564	0.03665
1202	ENSMUSG00000027805	Pfn2	50.01192	33.89826	20.64724	6.221645	10.20659	17.43201	-1.65972	0.036677
1203	ENSMUSG00000062937	Mtap	223.3866	334.9148	235.3786	503.9533	487.7292	418.3682	0.822853	0.036793
1204	ENSMUSG00000021485	Mxd3	35.00835	4.067791	12.38835	5.44394	0	1.394561	-2.93818	0.037026
1205	ENSMUSG00000027359	Slc27a2	7609.314	17519.3	8861.797	23687.36	22542.71	17786.23	0.913065	0.037067
1206	ENSMUSG00000026568	Mpc2	777.6854	1412.879	1094.304	3320.025	1798.547	1353.421	0.97819	0.037133
1207	ENSMUSG00000019737	Syne4	6.668256	0	4.129449	17.88723	18.22605	25.79937	2.582661	0.037137
1208	ENSMUSG00000020424	Castor1	64.18197	23.05081	53.68284	10.11017	8.748505	26.49665	-1.61983	0.03715
1209	ENSMUSG00000054514	Atad3aos	0	8.135581	115.6246	0	6.561379	6.275523	-3.2214	0.037193

Table S1. Differentially expressed genes from RNA-seq analysis of db/db mice livers (TAC vs. Veh; p-value < 0.05)

	Gene_ID	Gene_Name	Veh_1	Veh_2	Veh_3	TAC_1	TAC_2	TAC_3	log2FoldChange	p-value
1210	ENSMUSG00000062647	Rpl7a	374.2559	377.6266	70.20063	1181.335	651.7636	308.1979	1.367337	0.037219
1211	ENSMUSG00000038011	Dnah10	3.334128	3.389826	41.29449	3.888528	0	0	-3.5572	0.037245
1212	ENSMUSG00000020682	Mmp28	40.84307	2.033895	0	0	1.458084	2.091841	-3.63172	0.03755
1213	ENSMUSG00000037110	Ralgapa2	995.2373	637.2872	978.6794	283.8626	634.2666	450.4431	-0.93019	0.037602
1214	ENSMUSG00000051517	Arhgef39	0.833532	0.677965	0	9.332468	4.374253	11.85377	3.498093	0.037632
1215	ENSMUSG00000059734	Ndufs8	310.9075	553.2195	392.2976	1165.003	776.4298	509.712	0.962125	0.037653
1216	ENSMUSG00000067149	Jchain	2.500596	12.20337	0	18.66494	32.80689	27.89122	2.196208	0.037663
1217	ENSMUSG00000037580	Gch1	1136.938	837.2869	1127.34	1462.864	1755.533	2045.821	0.7648	0.037705
1218	ENSMUSG00000068036	Afdn	1819.6	770.1684	1449.437	611.2767	766.9523	775.3758	-0.90644	0.037768
1219	ENSMUSG00000025086	Trub1	86.68733	157.9659	45.42394	283.8626	124.6662	274.7285	1.20187	0.037899
1220	ENSMUSG00000049427	Gm9837	34.17481	2.71186	49.55339	10.88788	1.458084	3.486402	-2.40926	0.037919
1221	ENSMUSG00000085001	Rapgef4os2	5.001192	9.491512	0	73.10433	11.66467	6.972804	2.45952	0.037927
1222	ENSMUSG00000035864	Syt1	9.168853	38.64401	103.2362	42.77381	202.6737	225.9188	1.70953	0.037977
1223	ENSMUSG00000080845	Gm9115	5.834724	2.71186	16.5178	0	0	1.394561	-4.03996	0.038099
1224	ENSMUSG00000026701	Prdx6	2911.527	5709.144	3307.689	6696.046	7911.565	6726.664	0.838143	0.038181
1225	ENSMUSG00000034412	Tbc1d10a	86.68733	79.99988	49.55339	168.7621	108.6273	170.8337	1.017957	0.038197
1226	ENSMUSG00000036327	Qsox2	343.4152	111.1863	194.0841	109.6565	78.00751	113.6567	-1.10889	0.038278
1227	ENSMUSG00000044139	Prss53	0	2.71186	0	20.99805	8.748505	6.972804	3.317051	0.038309
1228	ENSMUSG00000067787	Blcap	561.8006	386.4401	487.275	290.0842	269.0165	274.0312	-0.78412	0.038364
1229	ENSMUSG00000025991	Cps1	2041.32	21712.51	4715.831	11617.37	42776.55	28014.63	1.533135	0.038744
1230	ENSMUSG00000052798	Nup107	96.68972	48.13552	136.2718	33.44134	20.41318	56.47971	-1.32113	0.038745
1231	ENSMUSG00000021143	Pacs2	199.2142	253.559	82.58898	175.7615	783.7203	356.3103	1.280509	0.038776
1232	ENSMUSG00000104520	Gm37336	5.834724	0	0	17.88723	14.58084	19.52385	2.923368	0.038814
1233	ENSMUSG00000104205	Gm37396	0	0	33.03559	0	0.729042	0	-5.30715	0.038859
1234	ENSMUSG00000086771	1700080G11Rik	1.667064	19.66099	8.258898	76.21516	21.87126	26.49665	2.03396	0.038977
1235	ENSMUSG00000046687	Gm5424	898.5476	2542.369	1581.579	1887.492	5002.687	3674.668	1.072427	0.03903
1236	ENSMUSG00000041439	Mfsd6	225.8872	85.4236	78.45953	41.99611	69.98804	57.87427	-1.21526	0.039042
1237	ENSMUSG00000028033	Kcnq5	0	1.35593	24.77669	0	0	0.69728	-4.93602	0.039042
1238	ENSMUSG00000020744	Slc25a19	118.3616	148.4744	206.4724	489.9546	334.6303	162.4663	1.075279	0.03919
1239	ENSMUSG00000018500	Adora2b	2.500596	1.35593	33.03559	2.333117	0	0	-3.90102	0.039203
1240	ENSMUSG00000031024	St5	756.8471	784.4056	322.097	221.6461	341.9207	380.7151	-0.98788	0.039211

Table S1. Differentially expressed genes from RNA-seq analysis of db/db mice livers (TAC vs. Veh; p-value < 0.05)										
	Gene_ID	Gene_Name	Veh_1	Veh_2	Veh_3	TAC_1	TAC_2	TAC_3	log2FoldChange	p-value
1241	ENSMUSG00000032278	Paqr5	0.833532	0	20.64724	0	0	0	-5.62763	0.039288
1242	ENSMUSG00000094488	Olfr393	0.833532	0	20.64724	0	0	0	-5.62763	0.039288
1243	ENSMUSG00000019874	Fabp7	41.6766	31.1864	276.6731	34.21905	50.30391	20.91841	-1.68866	0.039292
1244	ENSMUSG00000041881	Ndufa7	439.2714	662.3719	747.4303	2016.591	947.0257	748.1818	1.008287	0.039333
1245	ENSMUSG00000095524	Gm15682	15.83711	12.20337	70.20063	12.44329	7.290421	3.486402	-1.99587	0.039426
1246	ENSMUSG00000020087	Tysnd1	457.6091	717.9651	136.2718	929.3583	1052.008	927.3829	1.13731	0.039434
1247	ENSMUSG00000050069	Grem2	152.5364	153.8981	264.2847	104.9903	109.3563	73.21444	-0.96901	0.039502
1248	ENSMUSG00000027293	Ehd4	221.7195	142.3727	111.4951	58.32793	78.00751	99.01381	-1.02935	0.039538
1249	ENSMUSG00000051910	Sox6	424.2678	172.2031	656.5824	184.3162	183.7186	218.2488	-1.08436	0.039624
1250	ENSMUSG00000020186	Csrp2	135.8657	149.8303	119.754	370.1879	292.3459	149.9153	0.994802	0.039672
1251	ENSMUSG00000032384	Csnk1g1	134.1987	77.28802	342.7443	41.2184	86.02697	92.73829	-1.30621	0.039684
1252	ENSMUSG00000027227	Sord	1619.553	17425.06	5157.682	13350.87	23685.85	27603.24	1.417049	0.03984
1253	ENSMUSG00000055371	Stam2	325.911	235.9319	619.4173	161.7628	202.6737	223.827	-0.99147	0.039856
1254	ENSMUSG00000021913	Ogdhl	0	0.677965	20.64724	0	0	0	-5.61582	0.039991
1255	ENSMUSG00000113067	Gm47166	0	0.677965	20.64724	0	0	0	-5.61582	0.039991
1256	ENSMUSG00000097099	Gm9917	50.84546	43.38977	33.03559	10.88788	17.49701	20.91841	-1.38857	0.039998
1257	ENSMUSG00000074812	Spdye4c	10.00238	16.27116	0	0	0	1.394561	-4.27415	0.040001
1258	ENSMUSG00000044938	Klhl31	0.833532	3.389826	33.03559	1.555411	0	1.394561	-3.5352	0.040017
1259	ENSMUSG00000105156	Gm9057	10.83592	6.101686	61.94173	6.221645	0.729042	6.972804	-2.40975	0.040099
1260	ENSMUSG00000082271	Gm12217	0.833532	6.779651	0	22.55346	13.12276	18.82657	2.553212	0.040113
1261	ENSMUSG00000109104	Gm6108	3.334128	0.677965	24.77669	0	0	1.394561	-4.21613	0.040351
1262	ENSMUSG00000079259	Trim71	0	0.677965	41.29449	0.777706	1.458084	0	-4.14137	0.040365
1263	ENSMUSG00000003546	Klc4	326.7446	645.4228	441.851	977.576	902.5541	695.8858	0.863226	0.040414
1264	ENSMUSG00000042195	Slc35f2	82.51967	71.8643	57.81228	25.66429	37.18115	35.5613	-1.12473	0.04042
1265	ENSMUSG00000027506	Tpd52	500.9528	200.6777	619.4173	269.0862	209.2351	152.0071	-1.05975	0.040469
1266	ENSMUSG00000041460	Cacna2d4	0.833532	0.677965	0	7.777057	2.187126	16.73473	3.561509	0.040473
1267	ENSMUSG00000024987	Cyp26a1	1.667064	92.88122	28.90614	873.3635	47.38774	192.4494	3.164841	0.040516
1268	ENSMUSG00000111424	Gm48478	7.501788	0	74.33008	6.221645	0.729042	2.091841	-3.11549	0.040584
1269	ENSMUSG00000070690	5830473C10Rik	260.8955	578.3042	251.8964	853.1431	550.4268	746.09	0.969587	0.040625
1270	ENSMUSG00000028189	Ctbs	374.2559	180.3387	487.275	139.2093	160.3893	214.0651	-1.009	0.040646
1271	ENSMUSG00000035941	Ibtk	2868.184	720.6769	2362.045	619.0537	1050.55	1095.427	-1.10492	0.040679

Table S1. Differentially expressed genes from RNA-seq analysis of db/db mice livers (TAC vs. Veh; p-value < 0.05)										
	Gene_ID	Gene_Name	Veh_1	Veh_2	Veh_3	TAC_1	TAC_2	TAC_3	log2FoldChange	p-value
1272	ENSMUSG00000081284	Gm13673	28.34009	0	0	0	0	0.69728	-5.20617	0.040813
1273	ENSMUSG00000029761	Cald1	3700.882	2946.436	3064.051	864.8087	1967.685	2287.777	-0.92355	0.040839
1274	ENSMUSG00000071713	Csf2rb	277.5662	132.2032	247.7669	47.44005	126.1243	126.2077	-1.1276	0.040857
1275	ENSMUSG00000022762	Ncam2	7.501788	18.98302	0	18.66494	32.07785	66.24164	1.962366	0.040876
1276	ENSMUSG00000054757	Akr1c20	47.51133	891.5241	227.1197	829.0342	1259.056	1342.962	1.552304	0.040977
1277	ENSMUSG00000016495	Plgrkt	48.34486	70.50837	82.58898	233.3117	83.1108	124.1159	1.14952	0.041064
1278	ENSMUSG00000025207	Sema4g	2548.107	3514.571	2011.042	5639.921	3575.223	5032.273	0.818039	0.041215
1279	ENSMUSG00000030409	Dmpk	195.0465	209.4912	148.6602	429.2935	263.1842	320.749	0.860635	0.041315
1280	ENSMUSG00000020163	Uqcr11	378.4236	627.1177	689.618	1993.26	796.114	709.1341	1.04834	0.041344
1281	ENSMUSG00000022237	Ankrd33b	160.0382	232.542	41.29449	234.8671	544.5945	278.2149	1.258016	0.041383
1282	ENSMUSG00000060803	Gstp1	2640.63	2058.302	3538.938	1462.864	1668.777	1699.97	-0.76806	0.041499
1283	ENSMUSG00000016758	Bik	0	0	0	6.221645	6.561379	4.880963	4.592682	0.041569
1284	ENSMUSG00000075705	Msrb1	2155.514	2724.064	2044.077	5809.461	3589.074	2944.615	0.833382	0.041573
1285	ENSMUSG00000078864	Gm14322	31.67422	14.91523	57.81228	4.666234	8.748505	16.03745	-1.76254	0.041597
1286	ENSMUSG00000028874	Fgr	89.18793	30.50843	111.4951	33.44134	30.61977	27.19393	-1.30912	0.041656
1287	ENSMUSG00000022565	Plec	3081.568	795.2531	1854.123	679.7148	946.2967	1117.043	-1.06294	0.041752
1288	ENSMUSG00000040675	Mthfd1l	179.2094	92.20325	115.6246	66.88269	67.07187	59.26883	-1.00812	0.041767
1289	ENSMUSG00000031150	Ccdc120	418.4331	64.40669	317.9676	150.8749	65.61379	97.61925	-1.34627	0.041771
1290	ENSMUSG00000022969	Il10rb	130.031	90.16936	140.4013	69.99351	40.82636	64.84708	-1.02539	0.041805
1291	ENSMUSG00000044042	Fmn1	56.68018	49.49145	86.71843	10.11017	29.16168	34.16674	-1.36404	0.041845
1292	ENSMUSG00000051557	Pusl1	46.67779	51.52535	8.258898	138.4316	63.42666	89.25189	1.36545	0.04185
1293	ENSMUSG00000002006	Pdzd4	9.168853	4.067791	57.81228	1.555411	3.645211	6.972804	-2.43244	0.041883
1294	ENSMUSG00000058934	Igf1os	25.00596	6.779651	28.90614	174.2061	27.7036	17.43201	1.904857	0.042027
1295	ENSMUSG00000083708	Gm13123	4.16766	2.033895	0	10.88788	24.05839	9.761925	2.577929	0.042168
1296	ENSMUSG00000036875	Dna2	119.1951	96.27105	342.7443	80.10368	66.34283	94.13285	-1.18395	0.042214
1297	ENSMUSG00000046862	Pramef8	138.3663	49.49145	367.521	45.88463	98.42068	60.66339	-1.41253	0.042265
1298	ENSMUSG00000062818	Vmn1r51	2.500596	3.389826	8.258898	18.66494	29.16168	8.367365	2.218905	0.042353
1299	ENSMUSG00000042726	Trafd1	440.9385	372.8808	301.4498	692.9357	499.3938	764.2193	0.804173	0.042479
1300	ENSMUSG00000111947	Gm47438	0.833532	2.033895	74.33008	6.221645	0	0	-3.58724	0.042532
1301	ENSMUSG00000020400	Tnip1	345.9158	151.1862	322.097	119.7667	111.5434	177.1092	-0.99727	0.042535
1302	ENSMUSG00000112129	Pbld1	582.6389	3157.284	1011.715	3470.123	3451.285	3835.739	1.17713	0.042539

Table S1. Differentially expressed genes from RNA-seq analysis of db/db mice livers (TAC vs. Veh; p-value < 0.05)										
	Gene_ID	Gene_Name	Veh_1	Veh_2	Veh_3	TAC_1	TAC_2	TAC_3	log2FoldChange	p-value
1303	ENSMUSG00000030004	Nat8	249.2261	57.62703	165.178	771.484	204.8608	229.4052	1.355224	0.042563
1304	ENSMUSG00000018819	Lsp1	10.83592	18.98302	74.33008	9.332468	10.20659	7.670084	-1.84276	0.042624
1305	ENSMUSG00000029189	Sel1l3	270.0644	194.576	37.16504	56.77251	72.90421	75.30628	-1.31476	0.042681
1306	ENSMUSG00000034023	Fancd2	0.833532	11.52541	28.90614	0	2.187126	2.091841	-3.17613	0.042764
1307	ENSMUSG00000004552	Ctse	23.3389	36.61012	0	0.777706	6.561379	2.789122	-2.62605	0.042774
1308	ENSMUSG00000040726	Hesx1	15.00358	2.033895	0	0	0	0	-5.44997	0.042909
1309	ENSMUSG00000069862	Rps12-ps9	5.834724	1.35593	86.71843	6.999351	4.374253	3.486402	-2.57576	0.042916
1310	ENSMUSG00000032594	Ip6k1	457.6091	455.5926	429.4627	771.484	652.4927	778.8622	0.712704	0.043118
1311	ENSMUSG00000075014	Gm10800	1.667064	0	144.5307	12.44329	2.187126	0.69728	-3.22074	0.043151
1312	ENSMUSG00000116831	CT027991.1	24.17243	10.16948	0	87.88074	13.8518	46.02051	1.965414	0.043204
1313	ENSMUSG00000024423	Impact	305.9063	312.5419	297.3203	207.6474	169.1378	155.4935	-0.78435	0.043221
1314	ENSMUSG00000114321	Gm8971	0.833532	0	0	20.99805	2.187126	2.091841	4.093214	0.043269
1315	ENSMUSG00000059824	Dbp	131.6981	185.7624	128.0129	251.1989	173.512	500.6473	1.047183	0.043383
1316	ENSMUSG00000032066	Bco2	31.67422	122.0337	4.129449	133.7654	158.9312	196.6331	1.573282	0.043409
1317	ENSMUSG00000006412	Pfdn2	163.3723	162.0337	210.6019	664.9383	258.0809	206.395	1.082442	0.043411
1318	ENSMUSG00000030110	Ret	0	0	45.42394	1.555411	0	0	-4.82892	0.043414
1319	ENSMUSG00000030834	Abcc6	512.6222	2104.404	627.6762	1926.377	2407.297	2787.727	1.131111	0.043458
1320	ENSMUSG00000032741	Tpcn1	414.2654	591.8635	561.6051	1126.896	745.081	830.4609	0.78733	0.043685
1321	ENSMUSG00000114818	Gm35164	0	0	0	6.221645	10.93563	1.394561	4.663011	0.04384
1322	ENSMUSG00000035671	Zswim4	345.0823	56.2711	445.9805	66.10498	118.1048	140.8506	-1.37147	0.043896
1323	ENSMUSG00000042010	Acacb	961.0625	5698.975	594.6406	430.0712	1232.081	931.5666	-1.48521	0.043926
1324	ENSMUSG00000021451	Sema4d	3.334128	40.67791	57.81228	4.666234	5.103295	13.24833	-2.09984	0.043928
1325	ENSMUSG00000005354	Txn2	923.5535	1231.185	1143.857	2430.33	1647.635	1545.173	0.76994	0.043991
1326	ENSMUSG00000091144	Phf11c	65.0155	88.13546	8.258898	321.9701	87.48505	82.97637	1.556199	0.044046
1327	ENSMUSG00000047181	Samd14	6.668256	12.88134	0	46.66234	16.03893	26.49665	1.977647	0.04405
1328	ENSMUSG00000102649	Gm38021	0	0	0	14.77641	2.187126	2.091841	4.700133	0.044095
1329	ENSMUSG00000059089	Fcgr4	125.0298	63.72872	202.343	59.88334	73.63325	16.73473	-1.35982	0.044109
1330	ENSMUSG00000061079	Zfp143	90.85499	46.77959	330.3559	59.88334	43.01348	69.03076	-1.4111	0.04424
1331	ENSMUSG00000033544	Angptl1	3.334128	11.52541	45.42394	5.44394	3.645211	1.394561	-2.42245	0.044253
1332	ENSMUSG00000068011	Mkrn2os	359.2523	323.3894	342.7443	241.8665	186.6348	163.8609	-0.79175	0.044385
1333	ENSMUSG00000000938	Hoxa10	0	0	94.97733	0	0	0	-7.8631	0.044434

Table S1. Differentially expressed genes from RNA-seq analysis of db/db mice livers (TAC vs. Veh; p-value < 0.05)										
	Gene_ID	Gene_Name	Veh_1	Veh_2	Veh_3	TAC_1	TAC_2	TAC_3	log2FoldChange	p-value
1334	ENSMUSG00000085310	Gm11491	0	0	94.97733	0	0	0	-7.8631	0.044434
1335	ENSMUSG00000028572	Hook1	1285.306	1096.27	1350.33	400.5184	860.2697	800.4779	-0.85536	0.044503
1336	ENSMUSG00000107200	Gm43039	11.66945	10.84744	28.90614	6.221645	2.916168	1.394561	-2.21829	0.044905
1337	ENSMUSG00000028256	Odf2l	114.1939	101.0168	57.81228	228.6455	136.3309	184.082	0.975266	0.044947
1338	ENSMUSG00000023883	Phf10	465.1109	209.4912	503.7928	251.1989	147.9955	198.0276	-0.97351	0.045097
1339	ENSMUSG00000028333	Anp32b	710.1693	416.9485	1135.598	442.5145	377.6438	366.0722	-0.92455	0.045116
1340	ENSMUSG00000024055	Cyp4f13	461.7768	573.5585	338.6148	958.1334	694.7771	747.4846	0.796953	0.045186
1341	ENSMUSG00000051435	Fhad1	6.668256	12.88134	0	53.66169	19.68414	17.43201	2.007775	0.04522
1342	ENSMUSG00000025229	Pitx3	1.667064	0.677965	0	20.99805	8.019463	2.091841	3.302336	0.045269
1343	ENSMUSG00000005681	Apoa2	17802.58	56811.44	31970.19	126824.4	58495.42	43828.26	1.104261	0.045365
1344	ENSMUSG00000025494	Sigirr	78.35201	171.5252	119.754	259.7537	195.3833	260.0856	0.948707	0.045394
1345	ENSMUSG00000037622	Wdtc1	896.8805	1052.202	681.3591	1088.788	1693.565	1815.718	0.802229	0.045399
1346	ENSMUSG00000020130	Tbc1d15	1206.954	744.4057	1395.754	451.0693	724.6679	681.2429	-0.84694	0.045411
1347	ENSMUSG00000020275	Rel	333.4128	48.13552	198.2135	53.66169	104.253	70.42532	-1.34248	0.045444
1348	ENSMUSG00000018405	Mrm1	16.67064	13.5593	0	48.21775	26.97456	37.65314	1.715989	0.045474
1349	ENSMUSG00000086432	B430119L08Rik	27.50656	12.20337	28.90614	115.8781	59.05241	20.91841	1.552036	0.045616
1350	ENSMUSG00000046380	Jrk	26.67303	23.72878	86.71843	14.77641	6.561379	20.91841	-1.62893	0.045617
1351	ENSMUSG00000023947	Nfkbie	83.35321	23.72878	70.20063	33.44134	13.8518	16.73473	-1.45637	0.045632
1352	ENSMUSG00000055912	Tmem150a	865.2063	833.8971	479.0161	2574.206	1063.672	840.9201	1.036107	0.045802
1353	ENSMUSG00000098747	Gm27216	87.52087	89.49139	12.38835	330.5249	119.5629	85.76549	1.452142	0.045808
1354	ENSMUSG00000026435	Slc45a3	242.5578	756.6091	384.0388	795.5929	1159.906	809.5425	0.995587	0.045838
1355	ENSMUSG00000115768	Dpep2nb	0	0.677965	24.77669	0.777706	0	0	-4.89748	0.045863
1356	ENSMUSG00000046223	Plaur	20.8383	8.135581	49.55339	3.110823	9.477547	6.275523	-1.97108	0.045886
1357	ENSMUSG00000035783	Acta2	5.001192	43.38977	45.42394	10.11017	2.916168	9.761925	-2.0123	0.045966
1358	ENSMUSG00000108808	Gm45204	0	0.677965	24.77669	0	0	0.69728	-4.89769	0.046003
1359	ENSMUSG00000027006	Dnajc10	802.6914	372.8808	846.537	362.4108	389.3085	341.6674	-0.88249	0.046068
1360	ENSMUSG00000058486	Wdr91	143.3675	242.0335	82.58898	341.4128	274.1198	322.8408	0.977033	0.046129
1361	ENSMUSG00000048391	Olfr843	0	0	90.84788	0	0	0	-7.79851	0.046225
1362	ENSMUSG00000080818	Gm9434	0	0	90.84788	0	0	0	-7.79851	0.046225
1363	ENSMUSG00000032883	Acsl3	698.4999	494.9145	553.3462	91.76927	247.8743	458.8105	-1.12946	0.046228
1364	ENSMUSG00000044719	E230025N22Rik	4.16766	0	28.90614	1.555411	0	0	-4.34984	0.046246

Table S1. Differentially expressed genes from RNA-seq analysis of db/db mice livers (TAC vs. Veh; p-value < 0.05)										
	Gene_ID	Gene_Name	Veh_1	Veh_2	Veh_3	TAC_1	TAC_2	TAC_3	log2FoldChange	p-value
1365	ENSMUSG00000097246	Gm26890	7.501788	8.135581	20.64724	0	3.645211	0	-3.25684	0.046255
1366	ENSMUSG00000097336	Fendrr	27.50656	55.59314	8.258898	158.652	26.24552	104.5921	1.592172	0.046268
1367	ENSMUSG00000006373	Pgrmc1	8834.606	13453.54	8837.021	16263.38	17844.76	17319.75	0.724211	0.046311
1368	ENSMUSG00000103146	Gm37745	1.667064	0	41.29449	0	2.187126	0	-4.21221	0.046342
1369	ENSMUSG00000040813	Tex264	729.3405	863.0496	640.0646	1618.405	1099.396	1075.904	0.762007	0.046362
1370	ENSMUSG00000103492	Gm37412	0	0	37.16504	0	0.729042	0.69728	-4.60174	0.046384
1371	ENSMUSG00000108320	Gm44877	0.833532	1.35593	0	20.22035	6.561379	2.789122	3.280862	0.046471
1372	ENSMUSG00000085088	4931413K12Rik	3.334128	0.677965	20.64724	0	0.729042	0.69728	-3.94537	0.046597
1373	ENSMUSG00000021743	Fezf2	0	0	24.77669	0	0	0	-5.85788	0.046886
1374	ENSMUSG00000047225	Olfr684	0	0	24.77669	0	0	0	-5.85788	0.046886
1375	ENSMUSG00000065220	Gm25794	0	0	24.77669	0	0	0	-5.85788	0.046886
1376	ENSMUSG00000068806	Olfr1259	0	0	24.77669	0	0	0	-5.85788	0.046886
1377	ENSMUSG00000083007	Gm13623	0	0	24.77669	0	0	0	-5.85788	0.046886
1378	ENSMUSG00000083032	Gm8087	0	0	24.77669	0	0	0	-5.85788	0.046886
1379	ENSMUSG00000083280	Gm11838	0	0	24.77669	0	0	0	-5.85788	0.046886
1380	ENSMUSG00000085224	Gm13425	0	0	24.77669	0	0	0	-5.85788	0.046886
1381	ENSMUSG00000085290	Gm16129	0	0	24.77669	0	0	0	-5.85788	0.046886
1382	ENSMUSG00000086027	Gm11250	0	0	24.77669	0	0	0	-5.85788	0.046886
1383	ENSMUSG00000087133	Gm27192	0	0	24.77669	0	0	0	-5.85788	0.046886
1384	ENSMUSG00000093759	Scarletltr	0	0	24.77669	0	0	0	-5.85788	0.046886
1385	ENSMUSG00000095638	Gm8888	0	0	24.77669	0	0	0	-5.85788	0.046886
1386	ENSMUSG00000097734	B930082K07Rik	0	0	24.77669	0	0	0	-5.85788	0.046886
1387	ENSMUSG00000099337	Gm29486	0	0	24.77669	0	0	0	-5.85788	0.046886
1388	ENSMUSG00000099801	Gm29011	0	0	24.77669	0	0	0	-5.85788	0.046886
1389	ENSMUSG00000102529	Gm29740	0	0	24.77669	0	0	0	-5.85788	0.046886
1390	ENSMUSG00000103420	Gm37537	0	0	24.77669	0	0	0	-5.85788	0.046886
1391	ENSMUSG00000104963	Gm42974	0	0	24.77669	0	0	0	-5.85788	0.046886
1392	ENSMUSG00000108602	Klk1b10-ps	0	0	24.77669	0	0	0	-5.85788	0.046886
1393	ENSMUSG00000109333	Gm30790	0	0	24.77669	0	0	0	-5.85788	0.046886
1394	ENSMUSG00000109565	Gm6579	0	0	24.77669	0	0	0	-5.85788	0.046886
1395	ENSMUSG00000110759	Gm6607	0	0	24.77669	0	0	0	-5.85788	0.046886

Table S1. Differentially expressed genes from RNA-seq analysis of db/db mice livers (TAC vs. Veh; p-value < 0.05)										
	Gene_ID	Gene_Name	Veh_1	Veh_2	Veh_3	TAC_1	TAC_2	TAC_3	log2FoldChange	p-value
1396	ENSMUSG00000114098	Gm40660	0	0	24.77669	0	0	0	-5.85788	0.046886
1397	ENSMUSG00000115432	D130009I18Rik	0	0	24.77669	0	0	0	-5.85788	0.046886
1398	ENSMUSG00000116406	Gm49489	0	0	24.77669	0	0	0	-5.85788	0.046886
1399	ENSMUSG00000043964	Orai3	325.0775	197.9658	231.2491	94.10239	158.9312	150.6126	-0.9044	0.046901
1400	ENSMUSG00000026576	Atp1b1	313.4081	487.4569	144.5307	565.392	873.3924	500.6473	1.022342	0.046959
1401	ENSMUSG00000071633	Gm4952	349.2499	1056.27	573.9934	1028.127	1514.95	1322.044	0.963177	0.04696
1402	ENSMUSG00000044330	Gm9790	2.500596	5.423721	0	29.55282	14.58084	7.670084	2.434018	0.046994
1403	ENSMUSG00000039660	Spout1	53.34605	63.72872	0	125.9883	102.0659	124.1159	1.505801	0.047023
1404	ENSMUSG00000033819	Ppp1r16a	270.0644	232.542	37.16504	552.171	312.7591	427.4329	1.23559	0.047053
1405	ENSMUSG00000024782	Ak3	3499.168	3884.74	3443.96	6128.321	5455.422	5463.889	0.654542	0.047116
1406	ENSMUSG00000025158	Rfng	115.861	113.2202	49.55339	241.8665	145.8084	185.4766	1.002032	0.047151
1407	ENSMUSG00000078619	Smarcd2	345.0823	578.9822	470.7572	1084.122	778.617	624.7632	0.834519	0.047177
1408	ENSMUSG00000049799	Lrrc19	0	0	0	0	5.832337	17.43201	4.991744	0.0472
1409	ENSMUSG00000060681	Slc9a6	503.4534	208.1353	590.5112	195.2041	180.8024	276.123	-0.98966	0.047272
1410	ENSMUSG00000013539	Tango2	243.3914	465.0841	251.8964	772.2617	737.0616	364.6776	0.957681	0.047282
1411	ENSMUSG00000031592	Pcm1	1452.013	275.9318	1693.074	741.9312	281.4103	398.1471	-1.26467	0.047289
1412	ENSMUSG00000023262	Acy1	68.34963	248.1352	214.7313	455.7355	451.2771	237.0753	1.113958	0.047396
1413	ENSMUSG00000030847	Bag3	615.1467	276.6098	392.2976	223.9792	250.0614	225.2216	-0.87846	0.047471
1414	ENSMUSG00000039316	Rftn1	6.668256	25.08471	90.84788	0.777706	14.58084	10.45921	-2.18695	0.047569
1415	ENSMUSG00000019891	Dcbld1	150.8693	70.50837	123.8835	48.99546	65.61379	52.99331	-1.036	0.047672
1416	ENSMUSG00000020884	Asgr1	3355.8	4273.892	2985.592	9801.425	4791.265	4712.221	0.862265	0.047697
1417	ENSMUSG00000067847	Romo1	185.0441	140.3388	251.8964	882.6959	272.6617	177.8065	1.216737	0.047736
1418	ENSMUSG00000025371	Chmp6	244.2249	136.949	425.3332	80.88139	132.6857	164.5582	-1.07565	0.047811
1419	ENSMUSG00000032773	Chrm1	0	3.389826	8.258898	31.10823	8.748505	15.34017	2.573993	0.047857
1420	ENSMUSG00000039960	Rhou	2406.407	1223.727	1309.035	614.3875	831.837	1201.414	-0.9007	0.047882
1421	ENSMUSG00000087605	Gm15742	0	1.35593	66.07118	0.777706	4.374253	1.394561	-3.28317	0.047896
1422	ENSMUSG00000039653	Baat	1263.635	1989.15	1515.508	2476.993	2752.134	2627.352	0.719812	0.048
1423	ENSMUSG00000021033	Gstz1	3898.429	8726.767	5719.287	13667.4	10022.14	9320.547	0.84739	0.048059
1424	ENSMUSG00000078683	Mup1	0	0	0	5.44394	7.290421	4.183682	4.530483	0.048083
1425	ENSMUSG00000027654	Fam83d	25.83949	0.677965	0	0.777706	0.729042	0	-4.20989	0.048085
1426	ENSMUSG00000112400	Gm47725	0	2.033895	0	39.66299	0	4.880963	3.946491	0.048119

Table S1. Differentially expressed genes from RNA-seq analysis of db/db mice livers (TAC vs. Veh; p-value < 0.05)

	Gene_ID	Gene_Name	Veh_1	Veh_2	Veh_3	TAC_1	TAC_2	TAC_3	log2FoldChange	p-value
1427	ENSMUSG00000006356	Crip2	403.4295	228.4742	322.097	678.937	493.5615	509.0147	0.818656	0.048127
1428	ENSMUSG00000103696	Gm37531	0	0	86.71843	0	0	0	-7.73088	0.048164
1429	ENSMUSG00000109115	Gm44669	1.667064	29.1525	41.29449	3.110823	5.832337	5.578243	-2.25878	0.048173
1430	ENSMUSG00000097729	2310015A10Rik	79.18554	47.45756	173.4369	52.88399	32.07785	39.0477	-1.23264	0.048223
1431	ENSMUSG00000110243	Gm45631	0	2.033895	16.5178	0	0	0	-5.41624	0.048299
1432	ENSMUSG00000040660	Cyp2b9	387.5924	5397.28	813.5014	4883.214	7700.872	5924.094	1.486756	0.048317
1433	ENSMUSG00000027860	Vangl1	230.0548	75.25413	227.1197	90.99156	76.54942	82.27908	-1.0793	0.048408
1434	ENSMUSG00000051648	Kctd19	0	0	41.29449	0	0	1.394561	-4.79573	0.048718
1435	ENSMUSG00000090015	Gm15446	15.83711	21.69488	0	63.77186	51.76199	22.31297	1.715768	0.048721
1436	ENSMUSG00000040466	Blvrb	791.0219	912.541	904.3493	1938.82	1293.321	1166.55	0.754751	0.048743
1437	ENSMUSG00000021877	Arf4	2022.149	1515.93	2238.161	1346.986	1158.448	967.8252	-0.73248	0.048868
1438	ENSMUSG00000035673	Sbno2	2078.829	340.3385	1498.99	710.0453	454.9223	579.44	-1.1666	0.048933
1439	ENSMUSG00000061815	Rufy4	7.501788	71.8643	309.7087	59.10563	10.20659	30.68034	-1.93581	0.049001
1440	ENSMUSG00000028991	Mtor	1292.808	2131.522	1053.009	536.6169	904.0122	991.5327	-0.88259	0.049024
1441	ENSMUSG00000084143	Gm13400	0	2.033895	82.58898	0	0	0	-7.69574	0.049199
1442	ENSMUSG00000061947	Serpina10	7710.171	1627.116	5938.148	3407.129	2143.384	1329.016	-1.15061	0.049201
1443	ENSMUSG00000098188	Sowahc	234.2225	122.0337	309.7087	83.99221	156.7441	77.39812	-1.05152	0.049234
1444	ENSMUSG00000038954	Supt3	45.84426	47.45756	12.38835	129.8768	59.05241	79.48996	1.265949	0.049259
1445	ENSMUSG00000031570	Plpp5	661.8244	221.6946	652.4529	335.9688	211.4222	207.0923	-1.02104	0.049282
1446	ENSMUSG00000072999	Gm15401	0.833532	3.389826	41.29449	1.555411	2.916168	0	-3.24225	0.049327
1447	ENSMUSG00000021930	Spryd7	399.2619	202.0336	330.3559	196.7595	169.8668	139.4561	-0.87788	0.049427
1448	ENSMUSG00000033676	Gabrb3	1.667064	4.745756	0	35.77446	7.290421	6.275523	2.664588	0.049485
1449	ENSMUSG00000048371	Pdp2	523.4581	366.1012	437.7216	852.3654	605.834	777.4676	0.752136	0.049613
1450	ENSMUSG00000087380	2210408F21Rik	58.34724	74.57616	20.64724	111.2119	114.4596	119.2349	1.099303	0.049649
1451	ENSMUSG00000091955	Gm9844	9.168853	4.745756	16.5178	0.777706	2.187126	1.394561	-2.71635	0.049649
1452	ENSMUSG00000034353	Ramp1	5.001192	30.50843	4.129449	62.99416	43.74253	34.86402	1.719386	0.049733
1453	ENSMUSG00000027510	Rbm38	38.34247	21.01692	173.4369	14.77641	34.99402	23.70753	-1.60766	0.049737
1454	ENSMUSG00000028062	Lamtor2	181.71	304.4063	152.7896	470.5119	398.057	317.2626	0.877511	0.049782
1455	ENSMUSG00000109459	Gm39526	0	0	0	9.332468	5.832337	2.091841	4.557838	0.049862
1456	ENSMUSG00000031302	Nlgn3	1.667064	0	41.29449	2.333117	0	0	-4.14483	0.049918
1457	ENSMUSG00000112433	Gm30122	20.00477	51.52535	0	163.3182	27.7036	75.30628	1.803251	0.049948
