# Supplementary material for: Tacrolimus Inhibits Hepatic Ferroptosis Through Modulating SIRT7-Dependent NRF2 Activation in Diabetes
Source: Antioxidants (Basel). 2026 May 6;15(5):589. doi: 10.3390/antiox15050589 (PMC13203482; doi:10.3390/antiox15050589)
Supplement: Supplementary file 1 [file antioxidants-15-00589-s001.zip › Figures S1–S4.pdf]

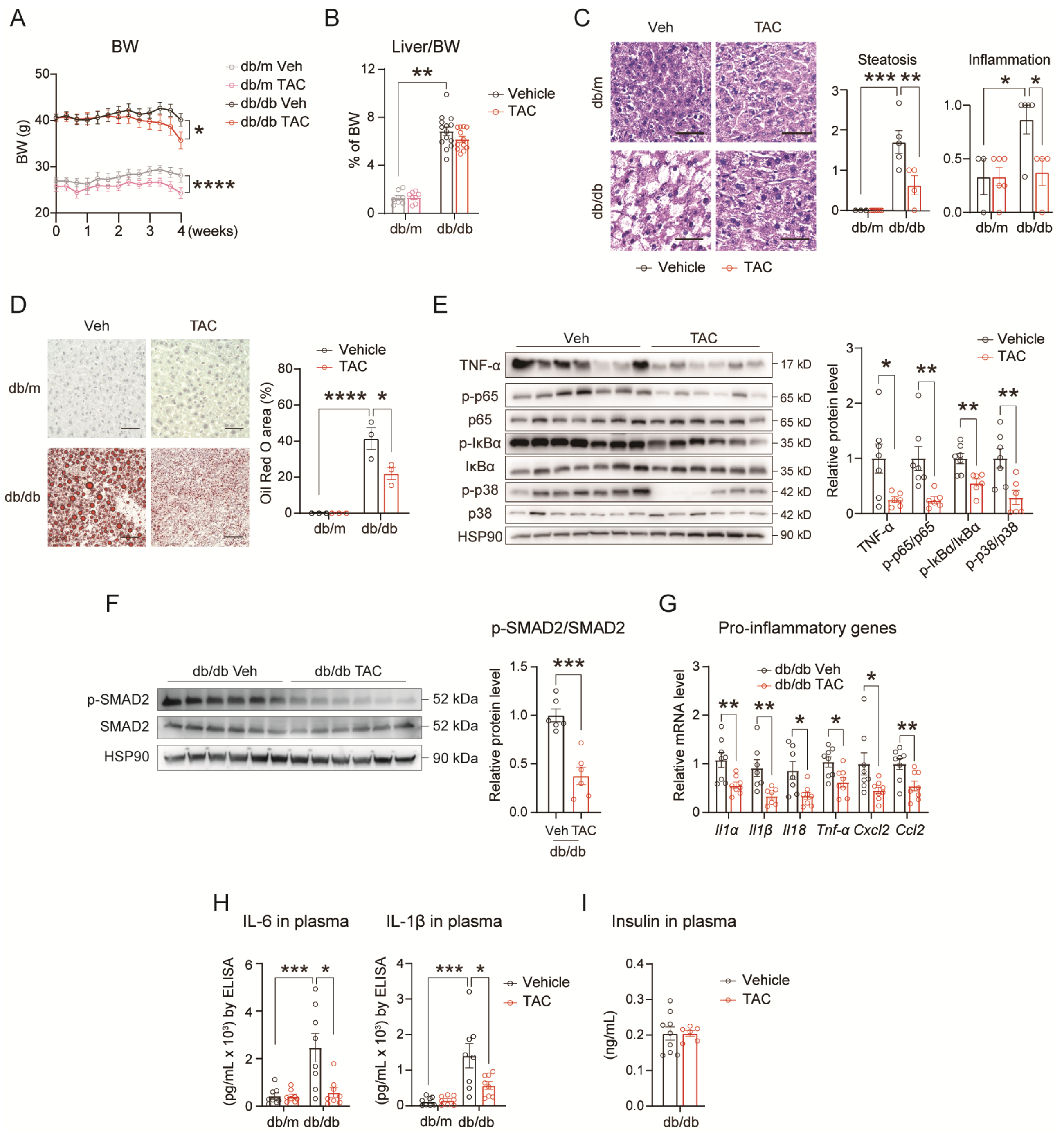

Figure S1. TAC ameliorates obesity and hepatic inflammation in db/db mice. (**A**) Body weights of db/m and db/db mice during TAC treatment. (db/m, n = 8/group; db/db, n = 12–14/group). (**B**) Liver weights (normalized to body weight) in db/m and db/db mice treated with TAC (db/m: n = 8; db/db: n = 12–14). (**C**) Representative H&E-stained liver sections from TAC-treated db/db and db/m mice. The quantifications for steatosis score and inflammation score are shown in the right (n = 3–6 for each group). Scale bar, 50 μm. (**D**) Representative images of Oil Red O staining of liver sections of db/m and db/db mice treated with TAC. The quantification is shown in the right (n = 3 for each group). Scale bar, 50 μm. (**E**) Western blotting analysis and quantification of TNF-α, p-p65, p65, p-IκBα, IκBα, p-p38, and p38 in db/db mice livers treated with or without TAC (n = 6–7 for each group). (**F**) Western blotting analysis and quantification of p-SMAD2 (Ser 255) and SMAD2 in db/db mice livers treated with or without TAC (n = 6 for each group). (**G**) Relative expressions of pro-inflammatory genes in TAC-treated db/db mice livers (n = 8 for each group), tested by qPCR. (**H**) ELISA analysis of IL-6 and IL-1β levels in plasma of TAC-treated db/m and db/db mice (n = 8 for each group). (**I**) Insulin levels in plasma of TAC-treated db/db mice, measured by ELISA (n = 6–9 for each group). Data are shown as mean ± SEM. *p* values are calculated using two-tailed unpaired Student's t test (**E**–**G**), one-way ANOVA (**B**–**D**,**H**) and two-way ANOVA (**A**), *$p < 0.05$, **$p < 0.01$, ***$p < 0.001$, and ****$p < 0.0001$.

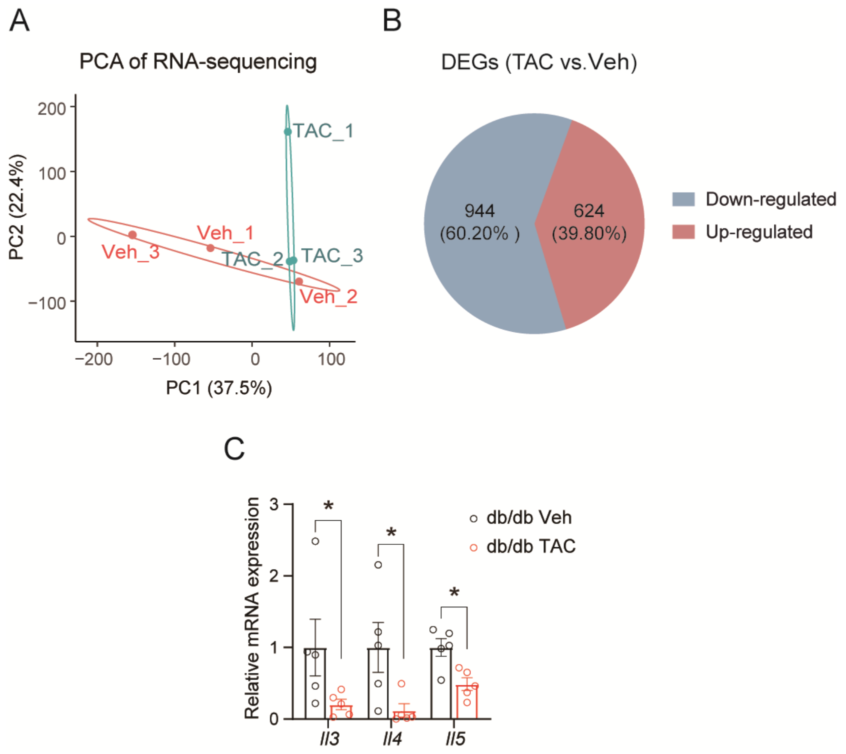

Figure S2. Transcriptomic analysis in TAC-treated db/db mice liver. (**A**) Principal Component Analysis (PCA) of RNA-sequencing analysis of TAC-treated db/db mice livers. Three independent biological replicates per group were randomly selected (n = 3 for each group). (**B**) The percentages of significantly up-regulated and down-regulated differentially expressed genes (DEGs) from RNA sequencing analysis in (**B**). The normalization and differential expression analysis were conducted using the DESeq2 package in R, with its standard median-of-ratios normalization. The criteria for identifying differentially expressed genes (DEGs) were set at $\log_2$ (fold change) ≥ 1.0 and adjusted p-value (FDR) < 0.05. (**C**) Relative expressions of *Il3*, *Il4* and *Il5* in TAC-treated db/db mice livers (n = 5 for each group), tested by qPCR. Data are shown as mean ± SEM. *p* values are calculated using two-tailed unpaired Student's t test (**C**), * $p < 0.05$.

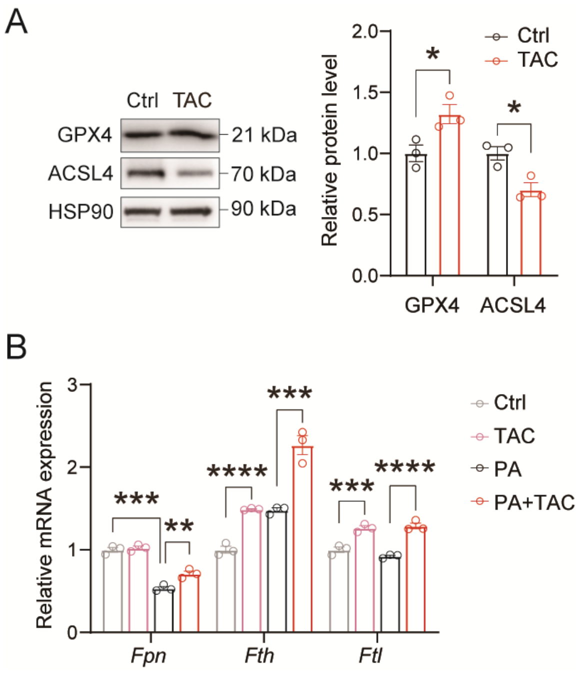

Figure S3. TAC modulates ferroptosis in primary hepatocytes. (**A**) Western blotting analysis and quantification of GPX4 and ACSL4 in primary hepatocytes treated with TAC (20 μM) for 24 h (n = 3 biological replicates). Hepatocytes were pre-treated with palmitic acid (PA, 0.5 mM, 24 h) to induce hepatic lipotoxicity. The quantification is shown in the right panel. (**B**) Relative expressions of ferroptosis-related genes in primary hepatocytes treated with TAC (20 μM) for 24 h (n = 3 biological replicates), tested by qPCR. Hepatocytes were pre-treated with or without palmitic acid (PA, 0.5 mM, 24 h) to induce hepatic lipotoxicity. Data are shown as mean ± SEM. p values are calculated using two-tailed unpaired Student's t test (**A**) and one-way ANOVA (**B**), * $p < 0.05$, ** $p < 0.01$, *** $p < 0.001$, and **** $p < 0.0001$.

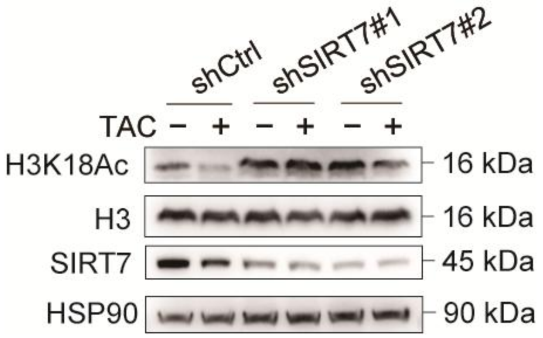

Figure S4. TAC enhances SIRT7 deacetylase activity in AML12 cells. Western blotting analysis and quantification of H3K18Ac in control and SIRT7 knock-down AML12 cells treated with TAC (20 μM) for 24 h, n = 3 biological replicates. AML12 cells were pre-treated with PA (0.5 mM, 24 h) to induce hepatic lipotoxicity.
